# Dihydroxyacetone suppresses mTOR nutrient signaling and induces mitochondrial stress in liver cells

**DOI:** 10.1371/journal.pone.0278516

**Published:** 2022-12-06

**Authors:** Arlet Hernandez, Manoj Sonavane, Kelly R. Smith, Jensyn Seiger, Marie E. Migaud, Natalie R. Gassman

**Affiliations:** 1 Department of Pharmacology and Toxicology, The University of Alabama at Birmingham, Birmingham AL, United States of America; 2 University of South Alabama Mitchell Cancer Institute, Mobile, Alabama, United States of America; 3 Department of Pharmacology, University of South Alabama Whiddon College of Medicine, Mobile, AL, United States of America; University of Nebraska-Lincoln, UNITED STATES

## Abstract

Dihydroxyacetone (DHA) is the active ingredient in sunless tanning products and a combustion product from e-juices in electronic cigarettes (e-cigarettes). DHA is rapidly absorbed in cells and tissues and incorporated into several metabolic pathways through its conversion to dihydroxyacetone phosphate (DHAP). Previous studies have shown DHA induces cell cycle arrest, reactive oxygen species, and mitochondrial dysfunction, though the extent of these effects is highly cell-type specific. Here, we investigate DHA exposure effects in the metabolically active, HepG3 (C3A) cell line. Metabolic and mitochondrial changes were evaluated by characterizing the effects of DHA in metabolic pathways and nutrient-sensing mechanisms through mTOR-specific signaling. We also examined cytotoxicity and investigated the cell death mechanism induced by DHA exposure in HepG3 cells. Millimolar doses of DHA were cytotoxic and suppressed glycolysis and oxidative phosphorylation pathways. Nutrient sensing through mTOR was altered at both short and long time points. Increased mitochondrial reactive oxygen species (ROS) and mitochondrial-specific injury induced cell cycle arrest and cell death through a non-classical apoptotic mechanism. Despite its carbohydrate nature, millimolar doses of DHA are toxic to liver cells and may pose a significant health risk when higher concentrations are absorbed through e-cigarettes or spray tanning.

## Introduction

Dihydroxyacetone (DHA) is a simple achiral triose that is the active ingredient in sunless tanning products (STPs) and is also a combustion production of propylene glycol and glycerol in electronic cigarette (e-cigarette) vapor. DHA is rapidly absorbed in cells and tissues through an unknown transporter [1–3]. Upon conversion to dihydroxyacetone phosphate (DHAP) by triose kinase/FMN cyclase (TKFC), it can be incorporated into several metabolic pathways, including glycolysis, and serves as a precursor to triglycerides [4]. The FDA approved DHA for topical use in sunless tanning lotions and cosmetics at concentrations up to 20% in the late 1970s [5]. However, the advent of spray tanning in the late 1990s increased the risk of internal exposure through inhalation and absorption through mucous membranes. The FDA has warned against these exposures and recommended protective equipment to reduce these exposures [6]. More recently, DHA was discovered in the vapor from e-cigarettes which has renewed concerns about inhalation exposure to DHA [1].

While it is unknown how much DHA may be inhaled or absorbed by spray tanning, Vreeke et al. have demonstrated e-cigarettes can release up to 2.33 μg DHA/puff depending on the electronic nicotine delivery system and its combustion characteristics [7]. Like traditional cigarettes, smoking and inhalation behaviors can vary significantly, but e-cigarette puff volumes are estimated at 58 ml, leading to a DHA concentration per puff of 10 nM to 42.5 μM. Each vaping session can result in exposure ranging from 0.14 to 600 μM, with most smokers engaging in multiple vaping sessions per day [7,8]. Depending on the frequency of vaping, it can be estimated that high micromolar to low millimolar doses of DHA are inhaled and systemically distributed in vapers. The European Commission on Scientific Committee on Consumer Safety estimates exposure levels from 0.04 mg to 0.61 mg of DHA for spray tanning applications, depending on the spray cabin style and protective measures used [9]. While spray tanning is more sporadic than vaping, the FDA has warned against inhalation exposures of DHA during spray tanning [6]. More quantitative studies are needed for vaping behaviors and spray tanning to understand exposure levels through inhalation and absorption.

With internal exposures now a significant risk, exposure effects of DHA on cells and tissues are needed to understand human health risks. Most DHA exposure effect studies have focused on skin models with DHA shown to induce reactive oxygen species [10], DNA damage, and replication stress in human immortalized keratinocytes, melanoma cell line A375P, and *ex vivo* skin models [11–13]. We have extended these studies to the human embryonic kidney cell lines HEK293T, where DHA exposure induced metabolic and mitochondrial stress [14]. Interestingly, all of the DHA exposure studies have identified cell-specific differences in the sensitivity of the cells to DHA and the induced cell death mechanism [12–14].

Exposure to 5 mM DHA was cytotoxic (IC_90_) in both A375P and HEK293T, but in the melanoma cell line, DHA rapidly produced ROS and cell cycle arrest within 24 h, followed by apoptotic cell death [13]. In the HEK293T cells, DHA displayed a delay in cell cycle arrest (72 h), produced little ROS, and cell death occurred through autophagy [14]. Changes in mitochondrial membrane potential were observed in both cell lines. A metabolic flux analysis on the HEK293T cells showed DHA reduced glycolysis and oxidative-phosphorylation, reducing the overall ATP and lactate production [14]. These findings are consistent with a recent study by Orozco et al. demonstrating that dihydroxyacetone phosphate (DHAP) can be used for glucose-sensing by regulating mTOR signaling [15]. In that work, glycolytic signaling was manipulated through CRISPR knockouts to identify critical signaling nodes in metabolism. DHAP and its conversion to glyceraldehyde-3-phosphate (GAP) dictated the cell’s metabolic signaling and, ultimately, metabolic fate [15]. DHAP levels were manipulated in that study by converting DHA to DHAP. The extent of this conversion is altered between cells and tissues, which have differing levels of TKFC [16]. This rate-limiting conversion step is also confirmed by hyperpolarized [2-^13^C]-DHA studies which demonstrated DHA was more readily metabolized in the liver than in the kidney [3].

Given the different metabolism observed between mouse kidney and liver tissues, we sought to examine the cytotoxic and metabolic impact of DHA exposure on the hepatoma cell line model HepG3 (HepG2/C3A). HepG3 cells are more differentiated and have a more metabolically active hepatic phenotype than their parental HepG2 cell line [17,18]. We examined the cytotoxic and metabolic effects of DHA with this cell line to acquire insight into systemic exposure effects.

## Material and methods

### Chemicals

Dihydroxyacetone (DHA) (CAS 26776–70–5, PHR1430) was purchased from Sigma-Aldrich (St. Louis, MO, USA) every 2 months and prepared fresh for each experiment. Chloroquine diphosphate salt (CAS 50-63-5, C6628) and rapamycin (CAS 53123-88-9 R0395) were also purchased from Sigma-Aldrich.

### Cell culture

The human hepatocellular carcinoma cell line (HepG2/C3A, also known as HepG3) was purchased from ATCC (CRL-10741 Manassas, VA). HepG3 cells were cultured with Dulbecco’s modified Eagle’s medium (DMEM, Hyclone, Logan, UT, USA; 4.5 g/L glucose) supplemented with 10% fetal bovine serum (FBS, Atlanta Biologicals, Flowery Branch, GA, USA), 2% Glutamax (Gibco, Carlsbad, CA, USA) and 1% sodium pyruvate (Gibco, Carlsbad, CA), and grown at 37°C in a 5% CO_2_ incubator. The cells were periodically tested using the Lonza MycoAlert kit (Walkersville, MD, USA) and found to be free of mycoplasma contamination.

### Cell viability studies

Cell viability was first confirmed in HepG3 cells using a growth inhibition assay [19]. Cells were plated in 6-well plates at a density of 60,000 cells per well and incubated in a 5% CO_2_ incubator at 37°C overnight (ON) to attach. The following day, cells were dosed in triplicate with increasing DHA concentrations (0.5, 1, 2.5, 5, 6.25, 7, and 10 mM) for 5 days. At the end of the exposure period, the culture medium was aspirated, and the cells were detached with 0.25% trypsin-EDTA (Gibco, Carlsbad, CA, USA) for 30 sec and resuspended in 1X phosphate-buffered saline (PBS, VWR Life Sciences, Radnor, PA, USA). Cell counts were determined by a Bio-Rad TC 20 automated cell counter (Bio-Rad, Hercules, CA, USA), and results were expressed as a percentage of survival of cells ± standard error of the mean (SEM). IC_50_ and IC_90_ values were calculated from the percentage growth relative to control using GraphPad Prism software.

Cell viability was also confirmed using a clonogenic assay. HepG3 cells were seeded at 15,000 cells per well in 12-well plates and incubated ON in a 5% CO_2_ incubator at 37°C. The following day, the cells were exposed to increasing concentrations of DHA (0.5, 1, 2.5, 5, 6.25, 7, and 10 mM) for 5 days. The plates were then placed on ice, and the culture medium was aspirated. Cells were washed twice with ice-cold 1X PBS before fixation with ice-cold methanol for 10 min. After the fixation, plates were stained with 0.5% crystal violet solution (prepared in 25% methanol, Thermo Fisher Scientific, Waltham, MA, USA) for 10 min at room temperature (RT, ~ 21°C). The solution was removed from the wells, and the excess stain was rinsed twice with double-distilled water (ddH_2_O). The plates were allowed to dry ON at RT. Stained plates were imaged with a Bio-Rad ChemiDoc XRS Imaging system (Bio-Rad, Hercules, CA, USA). Each image was quantified as the percentage of area fraction of the crystal violet staining in each well using the NIS-Elements software (Nikon, Melville, NY, USA). The experiment was performed in technical triplicates and averaged over three biological replicates with the final cell density percentage reported relative to control ± SEM. Using GraphPad Prism, IC_50_ and IC_90_ values were calculated from the average cell density percentage relative to the control.

Cell viability was also tested with chemical inhibitors, chloroquine, and rapamycin, in combination with DHA, using the same growth inhibition assay described above. HepG3 cells were dosed in triplicates with DHA (1.25 or 2.5 mM), chloroquine (1.25 or 2.5 μM), or rapamycin (1 or 10 nM) alone, and with combinations of DHA and chloroquine or DHA and rapamycin at indicated doses. Treatments and co-treatments were done continuously with no change in the growth medium.

### Cell cycle analysis

The cell cycle of HepG3 cells treated with DHA was performed using propidium iodide (PI), as previously described [14]. Briefly, four 100-mm dishes were seeded at 10^6^ cells and adhered ON at 37°C in a 5% CO_2_ incubator. The following day, cells were left untreated or treated with an IC_90_ dose of 7 mM DHA for 24, 48, and 72 h. After the exposure period, the culture medium was aspirated and stored in a 15 mL conical vial, and the cells were washed once with PBS. Cells were detached with 0.25% Trypsin-EDTA and resuspended in PBS. Cell number was determined using the Countess II Fl (Thermo Fisher Scientific, Waltham, MA, USA), and 10^6^ cells were collected for each exposure condition in a 15 mL conical tube. Cells were pelleted by centrifugation at 250 x g for 7 min, and the supernatant was decanted. The pellet was washed once with 2 mL PBS before being resuspended in 1 mL ice-cold PBS. Cells were fixed by dropping the cell suspension into 9 mL of 70% ethanol with continuous vortexing at 1,000 rpm to ensure thorough mixing and then stored at 4°C ON. The next day, cells were pelleted, washed, and pelleted again before being resuspended in PBS containing RNAse A stock solution (10 mg/mL) (Thermo Fisher Scientific). The cell suspension was transferred to a flow tube and incubated with RNAse A at 37°C for 10 min to digest RNA from the samples. At the end of the incubation period, 5 μL of propidium iodide (20 μg/mL) was added, followed by 15 min incubation at RT in the dark. Samples were run on a BD FACSCanto II (BD Biosciences, Franklin Lakes, New Jersey, USA) instrument and analyzed using FACSDiva software. Staining intensity was used to discriminate the cell cycle phases, as shown in representative figures. The values presented are the average percentage in each cycle ± SEM of the three biological replicates.

### Cell death assay

Cell death was measured using the Annexin/PI Apoptosis detection kit (Leinco Technologies, St. Louis, MO). Six 100-mm dishes were plated at a density of 0.75 x10^6^ cells and allowed to adhere ON at 37°C in a 5% CO_2_ incubator. The following day, cells were left untreated, treated with 7 mM DHA for 24, 48, 72, and 96 h, or 1 μM of camptothecin (CPT) (TCI Chemicals, Portland, OR, USA) for 24 h. Per the kit’s instructions, the culture medium was collected in 15 mL conical tubes at the end of exposure time. Cells were washed with PBS once, and 0.05% Trypsin (Gibco, Carlsbad, CA, USA) was added for 30 sec to detach the cells for re-suspension in the collected culture medium. The cells were pelleted at 250 x g centrifugation for 5 min at 4°C. The supernatant was aspirated, and the cell pellet was resuspended in ice-cold PBS. Cell number was obtained using the Bio-Rad TC-20 cell counter, and 250,000 cells per sample were added to flow tubes. The cells were pelleted, and 300 μL of binding buffer along with 5 μL of annexin and 5 μL of PI were added to the tubes and incubated at RT in the dark for 15 min. Samples were analyzed by flow cytometry using the BD FACSCanto II. Cells in each sample were categorized by staining levels of annexin and PI. Annexin positive and PI negative were classified as early-stage apoptotic cells, annexin positive and PI positive were classified as late apoptotic cells, while PI-stained cells were classified as necrotic cells. Data are presented as the average percentage of cells undergoing apoptosis and necrosis ± SEM.

### Immunofluorescence

The prevalence of strand breaks in HepG3 cells following exposure to DHA was determined by immunofluorescent staining of γH2AX. Cells were plated in an 8-well chamber at a density of 14,000 cells per chamber and allowed to adhere ON at 37°C in a 5% CO_2_ incubator. The following day, cells were left untreated or treated with 7 mM DHA for 24, 48, 72, and 96 h. After the exposure period, cells were fixed in 3.7% formaldehyde (Thermo Fisher Scientific, Waltham, MA, USA) in PBS for 10 min at RT, then washed three times with PBS. Cells were permeabilized with 1 mL of permeabilization buffer (Biotum, Fremont, CA, USA) for 10 min at RT, followed by washing three times with PBS. Cells were then blocked with 2% BSA in PBS for 30 min at RT. The phosphoSer139-Histone γH2AX (1:750, 07–164 Millipore Corp, Burlington, MA, USA) was added to the cells in solution and incubated for 1 h at RT. The excess antibody was removed by washing with PBS three times. Anti-mouse Alexa Fluor 546 (1:2000, A-11035, Thermo Fisher) was then added for 1 h at RT. Nuclear DNA was stained using DAPI (1:800, Thermo Fisher) for 10 min before the end of the incubation. The cells were washed three times in PBS and then imaged.

Imaging was conducted with a Nikon A1rsi confocal microscope with a 20X C-Apochromat (NA 0.75) air immersion objective. A minimum of 500 cells were imaged over three biological replicates for each time point. Nikon Elements software was used to create a region of interest around the nucleus, and the staining intensity of γH2AX was measured within the nucleus of each cell. Mean fluorescent intensity was calculated for each treatment condition, and the results are expressed as mean nuclear intensity ± SEM over all cells. A comparison between treatment conditions was conducted by one-way analysis of variance with Dunnett’s post hoc test using GraphPad Prism Software.

Cytochrome c release was evaluated using the immunofluorescence protocol as described above. At the end of the exposure period, the cell medium was removed and incubated for 15 min with 20 nM of Mitotracker CMXROS diluted in cell medium. The dye was removed and left to recover for 2 h in cell medium at 37°C in a 5% CO_2_ incubator. Following recovery, cells were fixed, permeabilized, and blocked. Cells were incubated with an anti-cytochrome c antibody (1:200, ab133504, Abcam, Cambridge, UK) for 1 h at RT. The excess antibody was removed by washing cells three times with PBS followed by anti-rabbit Alexa Flour 647 (1:400, Thermo Fisher) incubation for 1 h at RT. Nuclear DNA was stained by DAPI and incubated for 10 min before the end of the secondary antibody incubation. The cells were washed three times in PBS and imaged using the all-in-one fluorescence Keyence (BZ-X810) (Keyence, Osaka, Japan) with a 40X objective. A minimum of 50 cells for each time point were imaged and analyzed using the Nikon Elements software. A region of interest around the nucleus was created, and the staining intensity of cytochrome c was measured in the cytoplasm. Mean fluorescent intensity was calculated for each condition, and the results were presented as mean cytoplasmic intensity ± SEM over all cells. Using Graph Pad Prism, statistical analysis was performed using a student’s t-test over two biological repeats.

### Immunoblotting

HepG3 cells were seeded in 60-mm dishes at 10^6^ cells per dish and allowed to attach in a 5% CO_2_ incubator at 37°C ON. Following attachment, cells were left untreated or treated with 7 mM DHA for 1, 4, and 24 h. For 24, 48, 72, and 96 h time points, 0.75 x 10^6^ cells per dish were seeded in 100-mm dishes and left ON at 37°C in a 5% CO_2_ incubator. Cells were also dosed with chemical inhibitors chloroquine (2.5 μM) and rapamycin (10 nM) and co-exposed with DHA at 7 mM for 24, 48,72 h. Cells were also exposed to 1 μM CPT for 24 h. After their respective exposure time points, the culture medium was aspirated, and cells were scraped, washed with PBS, and pelleted. All samples were collected and stored at -80°C ON. The cells were lysed with ice-cold lysis buffer containing β-glycerophosphate, Tris-HCl, NaCl, 0.2% Triton X-100, 0.3% NP-40, plus Halt protease and phosphatase inhibitor (Pierce, Waltham, MA, USA). Cells were incubated on ice for 30 min, then centrifuged at 11,800 rpm for 15 min at 4°C, and the supernatant fraction was retained. Protein concentration was determined via the Bradford QuickStart protein assay kit (Bio-Rad, Hercules, CA, USA). 30 μg of protein was separated on 4–15% Mini-Protein TGX precast gel (Bio-Rad) ran at 120V for 1 h. The gel was then transferred to a nitrocellulose membrane (Bio-Rad) and blocked for 1 h at RT with 5% skim milk in Tris-buffered saline (TBS, VWR Life Sciences, Radnor, PA, USA) containing 0.1% Tween 20 (TBST). The membranes were then probed with antibodies detailed in Table 1.

**Table 1:** Antibodies used for immunoblotting experiments.

Antibodies by dilution	Source
**1:5000**	** **
α-Tubulin (T9026)	Millipore Sigma, St. Louis, MO, USA
**1:1000**	** **
4E-BP1 (53H11) (9644)	Cell Signaling, Danvers, MA, USA
AMPK⍺ (2532)	Cell Signaling
AKT (C67E7) (4691)	Cell Signaling
Cathepsin B (D1C7Y) (31718)	Cell Signaling
Cathepsin L (71298)	Cell Signaling
Caspase-1 (D7F10) (3866)	Cell Signaling
Caspase-2 (C2) (2224)	Cell Signaling
Caspase-3 (GTX13585)	GeneTex, Irvine, CA, USA
Cleaved Caspase 3 (Asp175) (5A1E) (9662)	Cell Signaling
Cleaved PARP (Asp214) (D64E10) (5625)	Cell Signaling
LAMP-1 (D2D11) (9091)	Cell Signaling
LC3B (PA1-46286)	Life Technologies, Carlsbad, CA, USA
mTOR (2972)	Cell Signaling
p-4E-BP1 (Thr 37/46)(236B4) (2855)	Cell Signaling
p-AKT (Ser473)(D9E) (4060)	Cell signaling
p-AMPK⍺ (2535)	Cell signaling
p-mTOR (Ser2448) (2972)	Cell Signaling
PARP-1 (556494)	BD Biosciences, Franklin Lakes, NJ, USA
RAPTOR (24C12) (2280)	Cell Signaling
RICTOR (53A2) (2114)	Cell Signaling
ULK1 (D8H5) (8054)	Cell Signaling
**1:500**	** **
Cyclin B1 (12231)	Cell Signaling
Cyclin D1 (8396)	Santa Cruz, Dallas, Texas, USA
p21 (397)	Santa Cruz
p-ULK1 Ser 555 (D1H4) (5869)	Cell Signaling

The next day, the membranes were washed three times with TBST, and an HRP-conjugated secondary antibody (Cell Signaling, Danvers, MA, USA) was added for 1 h. Membranes were then washed three times with TBST. Protein band intensity was measured using the Bio-Rad ChemiDoc XRS Imaging system and enhanced chemiluminescence (Advansta, San Jose, CA, USA). Detected bands were quantified using Image Lab software, and the band intensities were quantified relative to the loading control, α-tubulin on each gel. Unless otherwise stated, results are presented as the percentage of band intensity relative to control, untreated cells ± SEM over three biological replicates.

### XF Seahorse experiments

Mitochondrial respiratory function and glycolytic function in DHA-exposed HepG3 cells were measured using a Seahorse XFe96 cellular flux analyzer (Agilent Technologies, Santa Clara, CA, USA) according to the manufacturer’s recommendations. The cells were plated in an XFe96 cell culture microplate at a density of 15,000 cells/ 100 μl of growth medium and placed ON in a 5% CO_2_ incubator at 37°C. The next day, the cells were treated with medium only or treated with 7 mM of DHA for 24 h. The sensor cartridge was hydrated using autoclaved H_2_O, placed in a CO2-free incubator, and the calibrant solution added. On the day of the assay, cells were washed with warmed freshly prepared Seahorse serum-free XF medium (XF base medium supplemented with 25 mM glucose, 2 mM glutamine, and 1 mM sodium pyruvate; pH 7.4) and then replaced with 0.5 mL of fresh Seahorse assay medium. A direct cell count of each well was obtained with a Celigo Image cytometer (Nexcelom, Lawrence, MA, USA) and incubated immediately in a CO_2_-free incubator at 37°C for at least 45 min before running the plate in the Seahorse instrument. For the Mito Stress test assays, pre-warmed Oligomycin (Sigma-Aldrich, St. Louis, MO, USA), Carbonyl cyanide-4 (trifluoromethoxy) phenylhydrazone (FCCP) (Cayman Chemical, Ann Arbor, MI, USA), and Rotenone & Antimycin A (Sigma-Aldrich) were prepared and loaded into injector ports A, B, and C, respectively. The final concentrations of injections in the well were 1.5 μM oligomycin, 2 μM FCCP, 0.5 μM of rotenone, and antimycin A. The sensor cartridge plate loaded with the injections was incubated in a CO_2_-free incubator for at least 10 min before being placed on the XF96 analyzer for calibration. After calibration, the sensor cartridge was replaced with the culture plate. The assay measured the oxygen consumption rate (OCR) and extracellular acidification rate (ECAR) under basal conditions. A value was obtained for each well. The values were normalized to their direct cell count. Individual parameters were calculated for the Mito Stress test assay, such as basal respiration, ATP production, proton leak, maximal respiration, spare respiratory capacity, and non-mitochondrial respiration.

The same procedure was used for the Glycolysis Stress test with minor differences. The Seahorse serum-free XF medium was supplemented with only 2 mM of glutamine. The injections prepared were glucose (Thermo Fisher Scientific), oligomycin, and 2-deoxy-glucose (2-DG) (Sigma-Aldrich) and added to injector ports A, B, and C, respectively. The final concentrations injected into each well were 10 mM glucose, 1 μM oligomycin, and 50 mM 2-DG. The Glycolysis Stress test assay measured the OCR and ECAR under the basal conditions. Four parameters were calculated in this experiment: glycolysis, glycolytic capacity, glycolytic reserve, and non-glycolytic acidification. Assay graphs and parameters are presented as mean values relative to control ± SEM for both tests from three biological replicates.

### Total intracellular ATP levels

Cell Titer Glo assay (Promega, Fitchburg, WI, USA) measured total cellular ATP levels. Cells were seeded at 30,000 cells per well in a 6-well plate and incubated ON to attach in a 5% CO_2_ incubator at 37°C. The following day, cells were dosed with 7 mM DHA for 1, 4, and 24 h or left untreated. After completion of exposure times, the cell medium was aspirated and detached with 0.25% of Trypsin and resuspended in PBS. Cells were counted using the Countess II FI, and 10,000 cells per well were pipetted in triplicates into a white 96-well clear bottom plate. The Cell Titer Glo reagent was added in equal volume, mixed for 2 min, and then incubated for 10 min at RT. The plate was read in the Tecan Infinite M1000 plate reader. Results were expressed as the average percentage of the luminescent signal relative to the control ± SEM of three biological replicates.

### MitoSOX

To determine mitochondrial superoxide and other reactive oxygen species formed by DHA-exposed HepG3 cells, MitoSOX live imaging experiments are performed using the mitochondrial-targeted superoxide-sensitive fluorogenic probe MitoSOX^TM^ Red (Life Technologies, Carlsbad, CA, USA) as previously described [20]. Briefly, cells were plated at 20,000 cells per well in an 8-well chamber dish and left ON to attach at 37°C in a 5% CO_2_ incubator. The next day, cells were left untreated or dosed with 7 mM DHA for 24 and 48 h. At the end of the exposure period, the culture medium was aspirated, and 1 μM of MitoSOX^TM^ Red reagent (diluted in medium) was added to the plate and incubated for 10 min at 37°C. The concentration of MitoSox was optimized for the cell line and consistent with the method in [21]. After the incubation, the dye was removed, the cells were washed twice with PBS, and the cell culture medium was replaced with a phenol-free medium for imaging. Live cell imaging of stained cells was performed using a Nikon A1rsi confocal microscope with a 20X C-Apochromat (NA 0.75) air immersion objective. MitoSOX^TM^ Red stain images were obtained using the 561 nm laser at a resolution of 1024x1024. For analysis, a minimum of five images were taken for each time point. Binary masks were generated from each image, and the binary intensity of MitoSOX Red staining per image was calculated using the Nikon Elements software. At least 50 cells were measured for each time point, and the values were reported as fluorescence intensity of the mean binary intensity ± SEM for three biological replicates.

### Statistical analysis

Unless stated otherwise, data is represented by ± SEM of three biological replicates. Statistical analysis was performed using a one-way analysis of variance (ANOVA) with multiple comparisons and Dunnett’s post-hoc test and student’s t-test to compare between two groups in GraphPad Prism software. Statistical significance was defined by *p < 0.05, **p < 0.01, ***p < 0.001, ****p < 0.0001.

## Results

### DHA decreased cell viability and genotoxic effects in HepG3 cells

**Fig 1:**
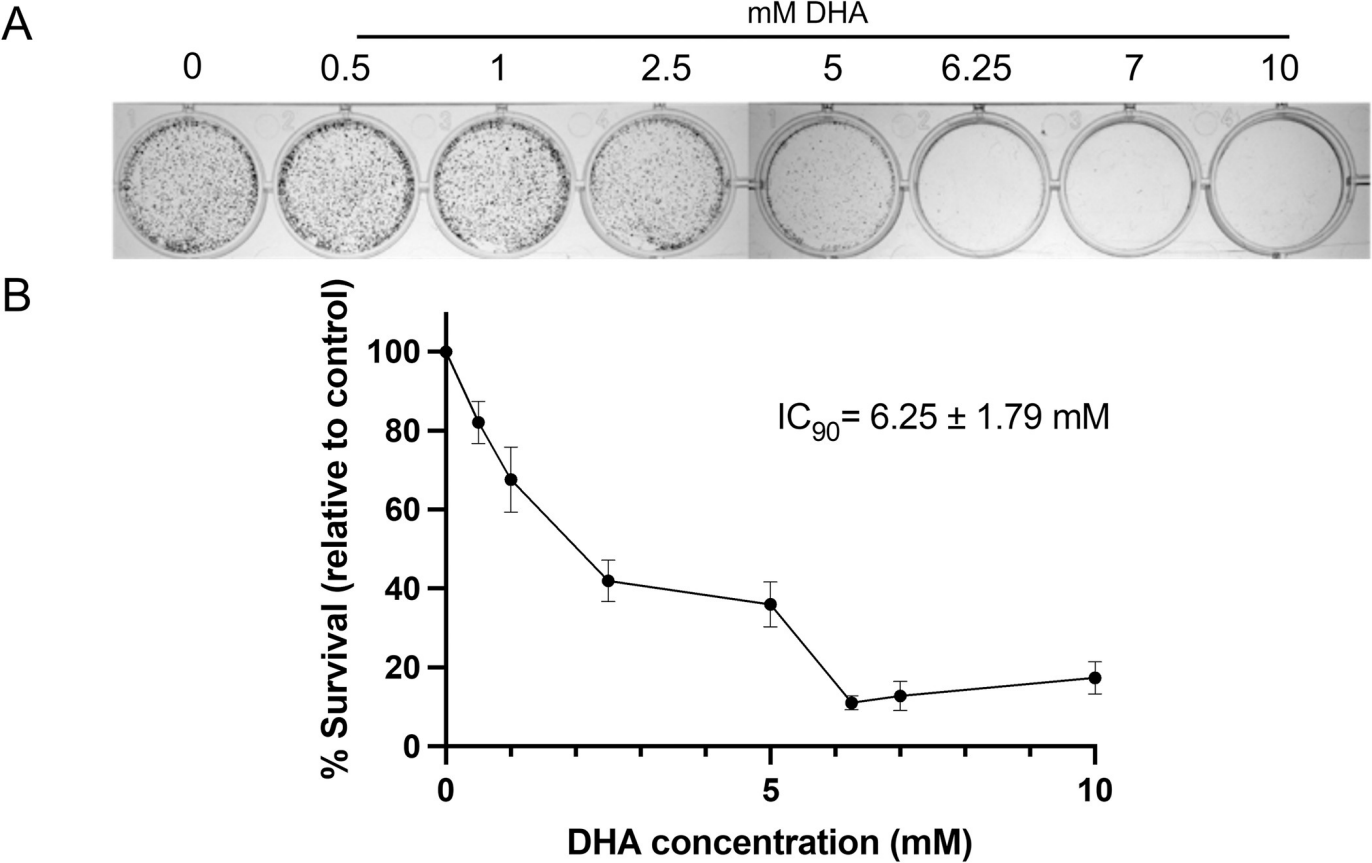
DHA decreased cell viability in HepG3 cells. (A) HepG3 cells were exposed to DHA doses ranging from 0–10 mM for 5 days. Cells were counted for each of the concentrations. (B) The survival percentage was calculated relative to the control and is inversely proportional to growth inhibition. The results are displayed using the mean ± SEM of each concentration. An IC_90_ of 6.25 ± 1.79 mM was calculated.

The effect of DHA on cell viability was assessed using a clonogenic assay. The cells were exposed to increasing concentrations of DHA, and clonal growth (viability) was assessed (Fig 1A). DHA reduced clonal growth at low millimolar concentrations with an IC_50_ and IC_90_ of 1.95 mM ± 0.98 and 6.25 ± 1.79 mM (mean survival ± SEM), respectively (Fig 1B). Cell growth and viability were also confirmed using a growth inhibition assay with similar IC_50_ and IC_90_ values (1.5 ± 0.98 mM and 4.6 ± 0.72 mM) calculated (S1 Fig).

**Fig 2:**
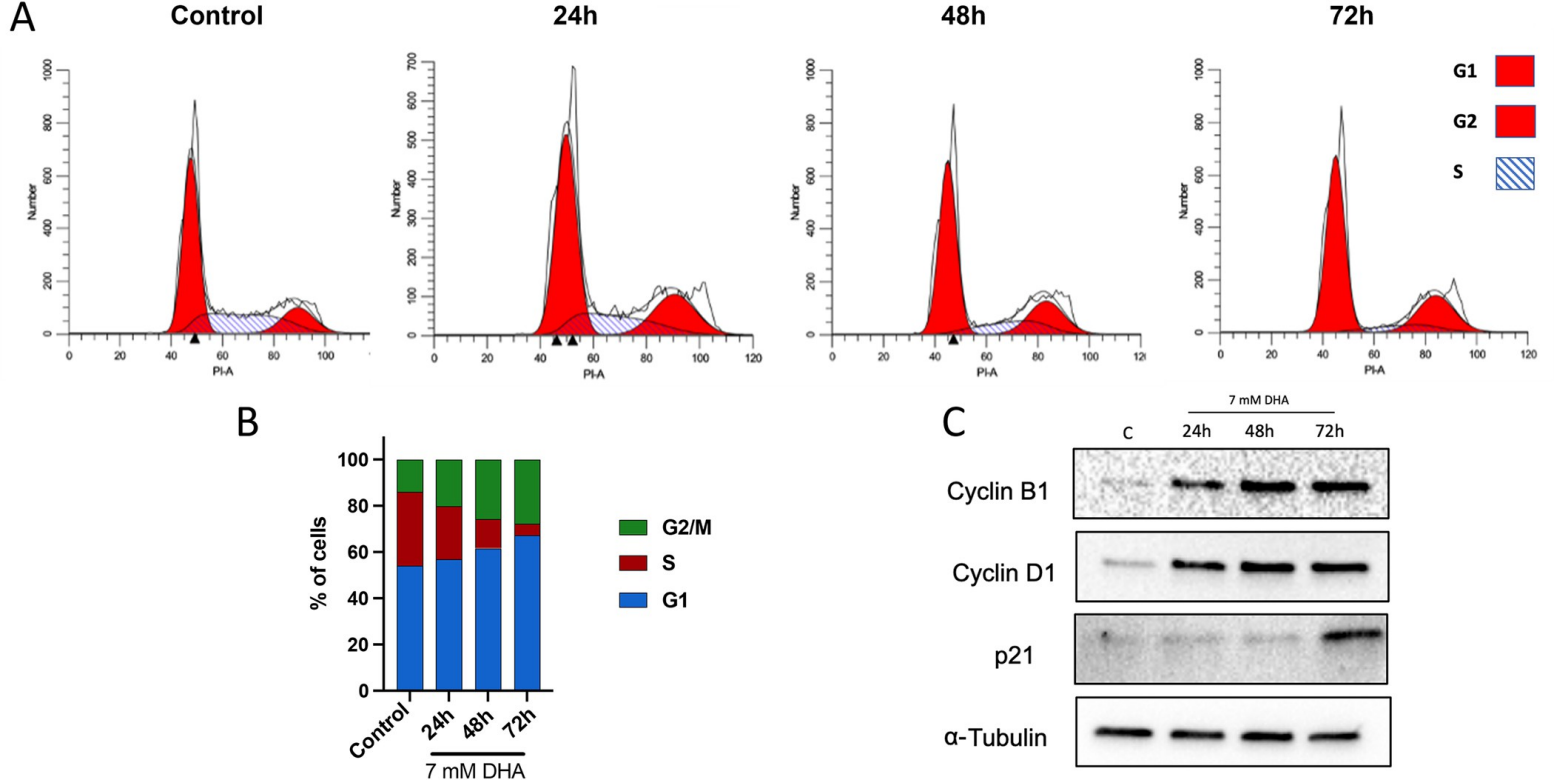
DHA induces weak cell cycle arrest at G1/S and G2/M. (A) HepG3 cells were dosed with 7 mM DHA and exposed for 24, 48, and 72 h. Changes in cell cycle phases were observed using PI. (B) After 48 h of exposure, the cell population in G1 and G2/M increased while S-phase cells decreased. (C) Cell cycle arrest was confirmed by cumulative increases in cyclin B1, cyclin D1, and p21 starting at 24 h.

We then assessed the genotoxicity of DHA at the IC_90_ dose of 7 mM. Genotoxic effects were evaluated using two experiments, cell cycle analysis via flow cytometry and immunofluorescent staining with γH2AX. After dosing cells with 7 mM DHA, we observed weak cell cycle arrest within the first 24 h of exposure (Fig 2A and 2B). Significant increases in both G1 (56.9 ± 0.48, p = 0.005), and G2/M phases (20.2 ± 1.9, p = 0.03) were observed. The percentage of cells arrested in these two phases increased significantly up to 72 h, while a significant decrease in the S phase was observed (5.0 ± 2.3, p = 0.0003). The weak cell cycle arrest at both checkpoints was confirmed by immunoblotting (Fig 2C) which showed an increase in both cyclin B1 and D1 starting at 24 h. Further, p21, a protein related to cell cycle arrest, increased throughout the exposure period. In addition to the replication stress observed, γH2AX staining showed a significant increase in γH2AX intensity in DHA-dosed cells at all time points (S2 Fig).

### Cell death is induced by 7 mM DHA exposure

**Fig 3:**
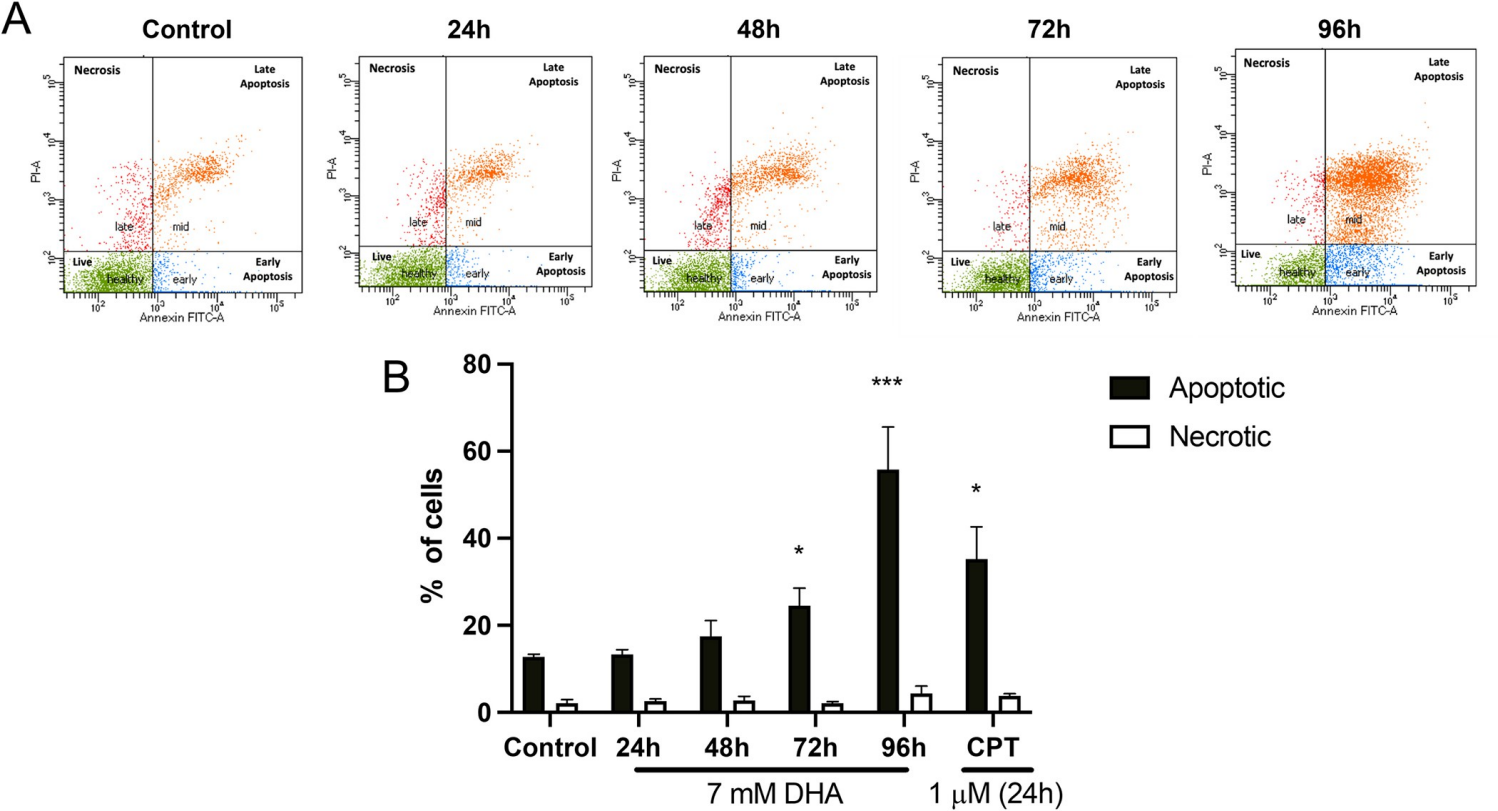
Cell death is mediated by apoptosis after exposure to DHA. (A) Cell death was evaluated at 24, 48, 72, and 96 h cells dosed with 7 mM DHA. Cells categorized in the early and mid-stages are apoptotic cells. Those in the late stage are necrotic cells. (B) A significant increase was calculated in apoptotic cells at 72 and 96 h, while necrotic cells stayed relatively constant throughout all time points. CPT, a positive cell death control, also showed a significant increase in the number of cells in apoptosis. The level of statistical significance was marked as follows: *p < 0.05; ***p < 0.001.

The cytotoxicity of DHA was assessed using Annexin/PI assay. The HepG3 cells were dosed with 7 mM of DHA for 24, 48, 72, and 96 h, and flow cytometry was used to evaluate the percentage of viable, apoptotic, and necrotic cells (Fig 3A). DHA-exposed cells showed an accumulation of apoptotic cells started at 48 h that continued to increase significantly at 72 and 96 h. A low percentage of necrotic cells remains constant throughout all time points. CPT was used as a positive apoptotic control and showed a significant increase in apoptotic cell death at 24 h (Fig 3B).

**Fig 4:**
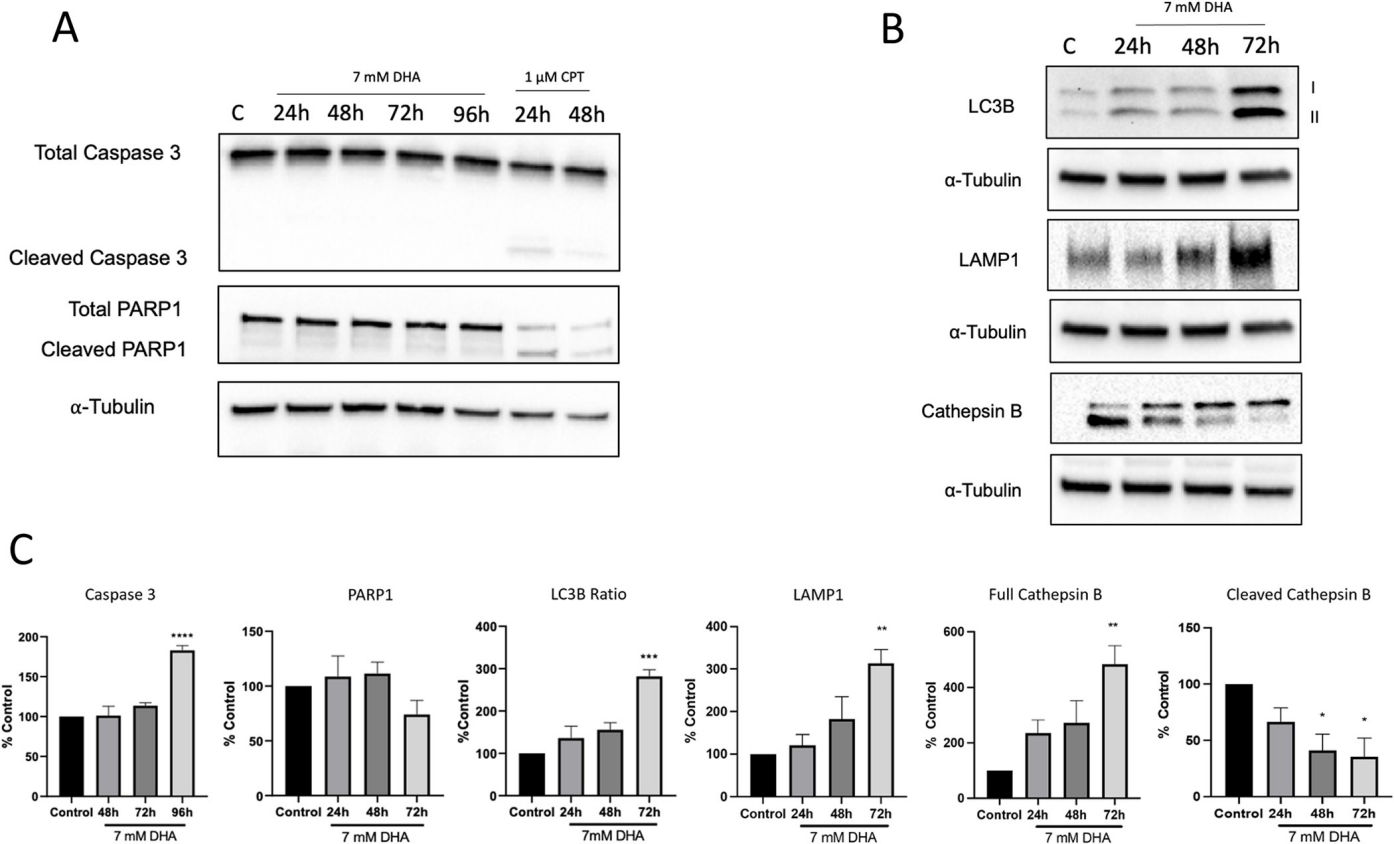
Cell death markers were evaluated in 7 mM DHA-exposed HepG3 cells. (A) Caspase 3 and PARP-1 proteins were probed in cells dosed with 7 mM DHA from 24 to 96 h exposure and CPT 1uM for 24 h and 48 h. A cleaved band for caspase 3 and PARP1 is present in cells dosed with CPT. CPT serves as a positive apoptotic control. Immunoblotting of samples dosed with CPT was performed in one biological repeat. (B) LC3B, LAMP-1, and cathepsin B levels at 24, 48, and 72 h. (C) Quantification of expression levels changes in cell death markers. The level of statistical significance was marked as follows: *p < 0.05; **p < 0.01; ***p < 0.001.

To confirm the mechanism of cell death, we then probed for the presence of various cell death markers over a 96 h exposure period. Previous studies have shown that DHA exposure causes cell death through multiple mechanisms, and effects were cell-type dependent [12–14]. We probed for the classic apoptotic pathway by examining the exposed cells for cleaved caspase 3 and PARP-1. Full-length Caspase 3 was observed over a 96 h exposure period. A significant increase was found in the full-length protein at 96 h compared to the control (Fig 4C). No changes in the full-length protein were observed at 24 h since HepG3 cells have a 48 h cycling period (S3B Fig). Caspase 1 and 2 were also probed but found no change in their expression levels (S3A Fig). However, no cleaved protein was observed for PARP-1 or Caspase 3 at any time point in HepG3 cells. We again used CPT as an apoptotic control to validate the lack of cleavage in these proteins. Cells were dosed with 1 μM CPT for 24 h and 48 h, and cleaved caspase 3 and cleaved PARP-1 were found in the CPT-dosed cells (Fig 4A).

We also examined the autophagy marker LC3B since HEK293T cells underwent an autophagic cell death [14]. A gradual increase in the ratio of LC3BII to LC3BI was observed at 24 and 48 h and significantly increased at 72 h compared to control untreated cells (Fig 4B and 4C). LC3BII intensity was also quantified as previous work has suggested LC3BII intensity only to be the indicator of autophagic cell death (S4 Fig) [22]. These trends are consistent with a significant increase in autophagy at 72 h observed by both the LC3BII alone and the LC3B ratio.

An increase in LAMP-1 was also observed starting at 24 h, which indicated lysosomal stress (Fig 4C). With the increase in autophagic markers aligning with the increasing population of apoptotic cell death (Fig 4), we examined lysosomal mediated cell death through cathepsin [23]. A gradual increase in full cathepsin B protein levels was observed starting at 24 h and significantly at 72 h, while cleaved cathepsin B decreased significantly at 48 and 72 h compared to control untreated cells (Fig 4C).

The lack of clear apoptotic markers and the presence of LC3BII and lysosomal stress led us to try and confirm the role of autophagy in cell death. Chemical inhibitors of mTOR, rapamycin, and autophagy, chloroquine, were combined with DHA, and a cell viability assay was performed (S5A and S6A Figs). Minimal cell death occurred when dosed with rapamycin or chloroquine alone. Co-dosing with rapamycin showed a modest increase in cell death (66.4 ± 4.8% 1.25 mM DHA vs. 37.2 ± 4.9% 1.25 mM DHA and 10 nM rapamycin, p-value = 0.001). Co-dosing with chloroquine showed a more significant increase in cell death (68 ± 6.6% 1.25 mM DHA vs. 20.50 ± 4.84% 1.25 mM DHA and 2.5 μM Chloroquine, p-value = 0.0070).

Associated proteins were probed to confirm inhibitor action and impact on autophagy markers through immunoblotting (S5B, S5C, S6B and S6C Figs). After rapamycin and DHA treatment, mTOR increased at 24 h while DHA alone increased at 72 h (S5B Fig). Increased LC3BII alone at 48 and 72 h was observed in the DHA and rapamycin-treated cells compared to 72 h for DHA alone (S5B Fig). Interestingly, we observed cleaved PARP1 at 72 h but no corresponding cleavage of caspase 3 (S5C Fig). For the chloroquine and DHA combination, LC3BII alone decreased at 72 h compared to DHA alone. No change in apoptotic markers, caspase 3 and PARP1, was observed with chloroquine and DHA (S6B and S6C Fig). These results suggest autophagy contributes to cell death, but inhibition does not revert the cell death mechanism to caspase-mediated apoptosis.

Changes in autophagic and lysosomal markers, along with increased full-length caspase 3, correlate with mitochondrial damage and cytochrome c release [24]. Therefore, we examined the localization and intensity of cytochrome c in HepG3 cells using immunofluorescence. Consistent with the annexin/PI assay results, an increase in cytochrome c staining intensity was observed at 48 h and gradually increased until 96 h with a significant two-fold increase compared to control (S7 Fig). The cytochrome c is mostly localized within the mitochondria, with some release into the cytoplasm observed.

### DHA regulates mTOR-specific signaling and nutrient sensing mechanisms

**Fig 5:**
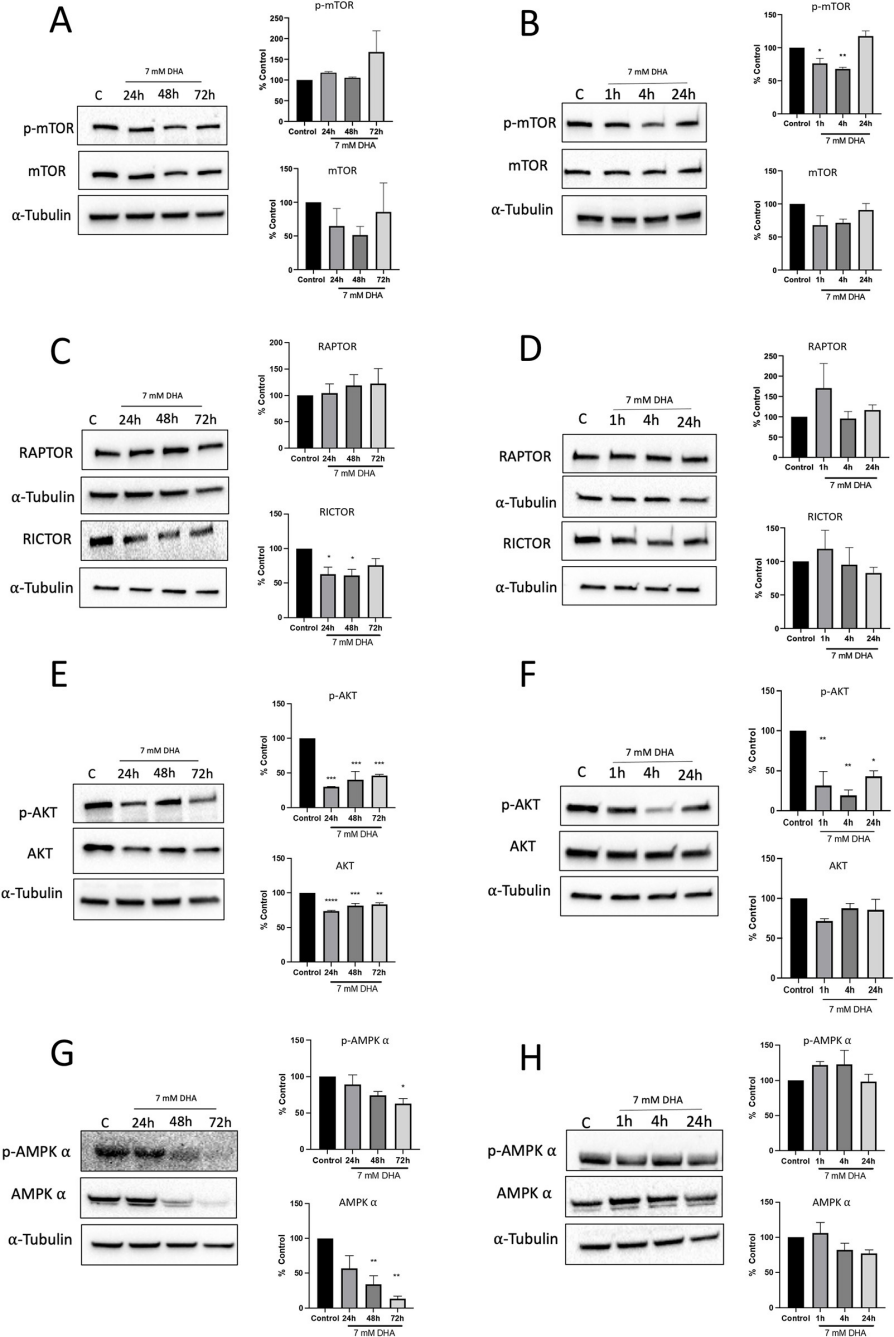
Changes in nutrient sensing mechanisms when cells are exposed to DHA. (A) mTOR signaling was evaluated using p-mTOR and mTOR markers when dosed with 7 mM DHA at 24, 48, and 72 h. Slight increases in p-mTOR were found, while mTOR displayed fluctuating levels at these time points. (B) p-mTOR and mTOR levels at 1, 4, and 24 h showed a significant decrease at 1 and 4 h, while mTOR decreased slightly at all time points. (C) RICTOR significantly diminished at 24 and 48 h, while RAPTOR slightly increased. (D) RICTOR and RAPTOR levels displayed fluctuating levels at 1, 4, and 24 h. (E) p-AKT and AKT levels showed significant decreases at 24, 48, and 72 h. (F) Only p-AKT levels significantly decreased at 1, 4, and 24 h. (G) p-AMPKα and AMPKα decreased gradually starting at 24 h and AMPKα significantly decreased at 48 and 72 h. (H) p-AMPKα showed a slight increase in expression levels at 1 and 4 h, while AMPKα slightly increased at 1 h and then decreased at 4 and 24 h. The level of statistical significance was marked as follows: *p < 0.05; **p < 0.01; ***p < 0.001.

With the increased autophagy marker LC3B, we probed for the mTOR-specific autophagy marker, phospho-ULK1, and total ULK1 activated by mTOR in autophagic cells [25]. Protein expression levels doubled at 24 h and increased up to 96 h in DHA-exposed cells (S8 Fig). We then examined mTOR signaling after DHA exposure [26,27]. Immunoblotting showed altered expression patterns of phospho-mTOR (p-mTOR) (Ser2448) and mTOR expression in cells exposed to 7 mM DHA (Fig 5A). p-mTOR increased non-significantly up to 72 h, and mTOR decreased at 24 h and 48 h. As factors influencing mTOR-specific signaling can fluctuate rapidly, mTOR markers were also probed at short time points, which showed a decrease in p-mTOR at 1 and 4 h, while total mTOR also decreased at these time points (Fig 5B). We also assessed the formation of mTOR complexes through RICTOR, part of mTOR complex II, and RAPTOR, part of mTOR complex I, changes at both long and short time points. RICTOR decreased significantly at 4, 24, and 48 h (Fig 5C and 5D). RAPTOR increased at 1 h and stayed constant from 24 to 72 h after DHA exposure (Fig 5C and 5D). We then probed the eukaryotic translation initiation factor 4E-binding protein 1 (4E-BP1), a central node of several signaling pathways sensing nutrients downstream of mTORC1, to validate the changes found in mTOR-specific signaling. mTORC1 inhibits 4EBP1, and as an increase is found in RAPTOR, this correlates with the slight decreases found starting at 1 h in phosphorylated and total 4E-BP1 protein (S9 Fig) [25].

We also examined AKT, which regulates the two mTOR complexes [26,27]. We observed a significant decrease in p-AKT (Ser473) at all time points and AKT at 24, 48, and 72 h (Fig 5E and 5F). As mTOR and AKT signaling changes were detected, AMPKα expression levels were also measured. A gradual decrease in p-AMPKα and total AMPKα levels starting at 24 h and significantly decreased until 72 h in cells dosed with 7 mM DHA (Fig 5G). A slight increase was observed in p-AMPKα levels at 1 and 4 h, while total AMPKα slightly decreased at 4 and 24 h in DHA-dosed cells (Fig 5H).

### Cellular energetics are modified in response to DHA exposure

Changes in mTOR-specific signaling and nutrient sensing nodes can be altered by upstream events such as energy, glucose, or amino acid levels [26,27]. To probe these events, changes in total ATP levels were assessed. Total cellular ATP levels fluctuated at 1 and 24 h, though the changes were insignificant (S10 Fig). Given these changes in ATP levels, a more detailed analysis of metabolic pathways was performed using flux analysis.

**Fig 6:**
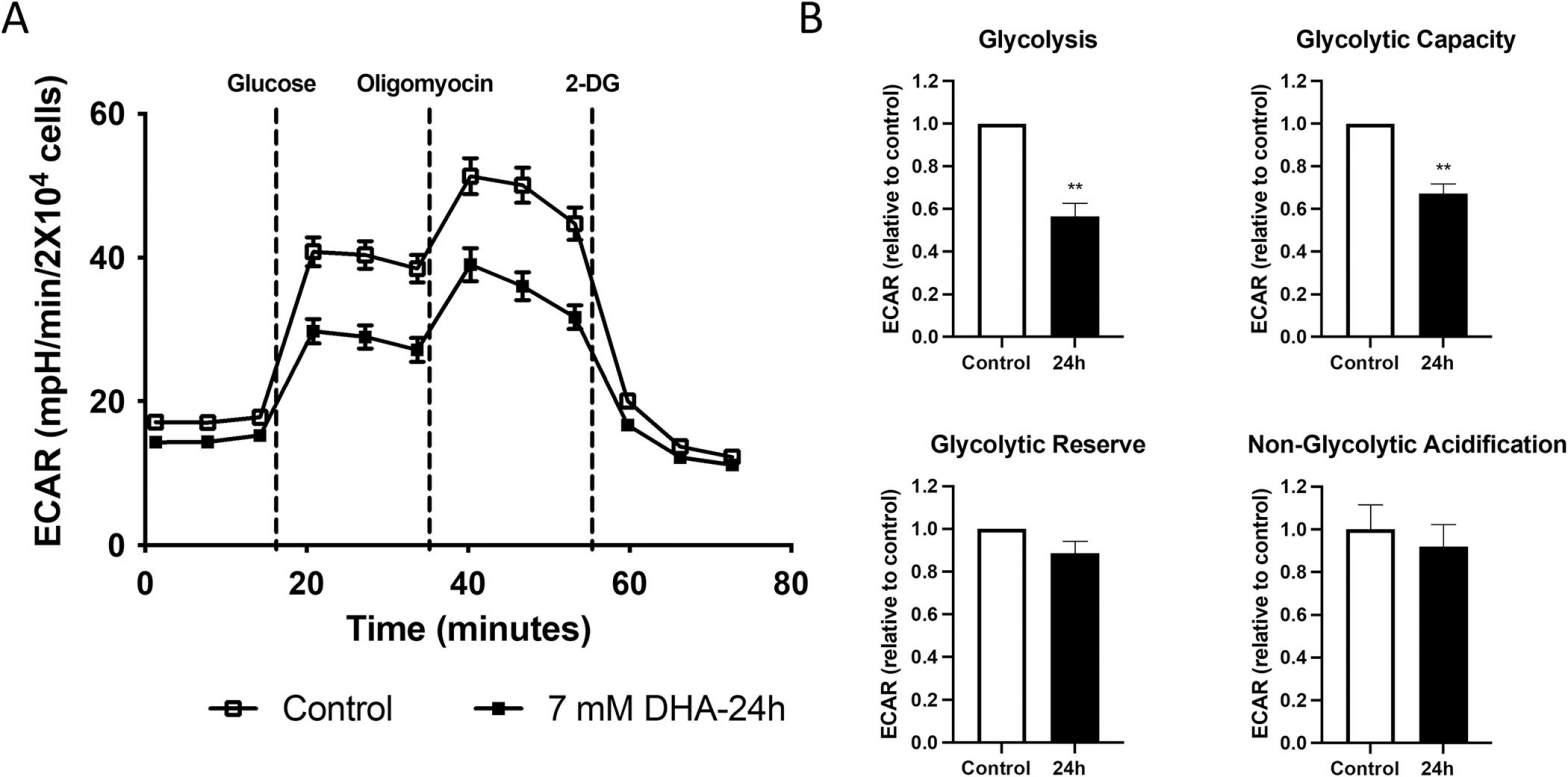
Glycolysis is modulated after DHA exposure. (A) Extracellular acidification rate (ECAR) displayed a shift in cells dosed with 7 mM DHA for 24 h. (B) All measured parameters showed a decrease with a significant difference in the glycolysis and glycolytic capacity. The statistical significance level was marked as **p < 0.01.

Seahorse XF Glycolysis Stress Test evaluated the effects of DHA exposure in the Glycolysis pathway. HepG3 cells were treated with the IC_90_ dose of 7 mM DHA for 24 h, and the extracellular acidification rate (ECAR) was measured. DHA-exposed cells showed decreased ECAR compared to untreated cells (Fig 6A). At all stress points within the assay, DHA-exposed cells showed declines in all parameters measured, with a significant reduction in glycolysis and glycolytic capacity (Fig 6B). Table 2 summarizes the effects of DHA exposure measured by the Glycolysis Stress Test.

**Table 2:** Flux analysis summary for DHA-exposed HepG3 cells.

Parameter	Effect (7 mM DHA)
**Glycolysis**	45% decrease
**Glycolytic capacity**	35% decrease
**Glycolytic reserve**	<10% decrease
**Non-glycolytic acidification**	<10% decrease

### Mitochondrial stress is generated by DHA exposure

**Fig 7:**
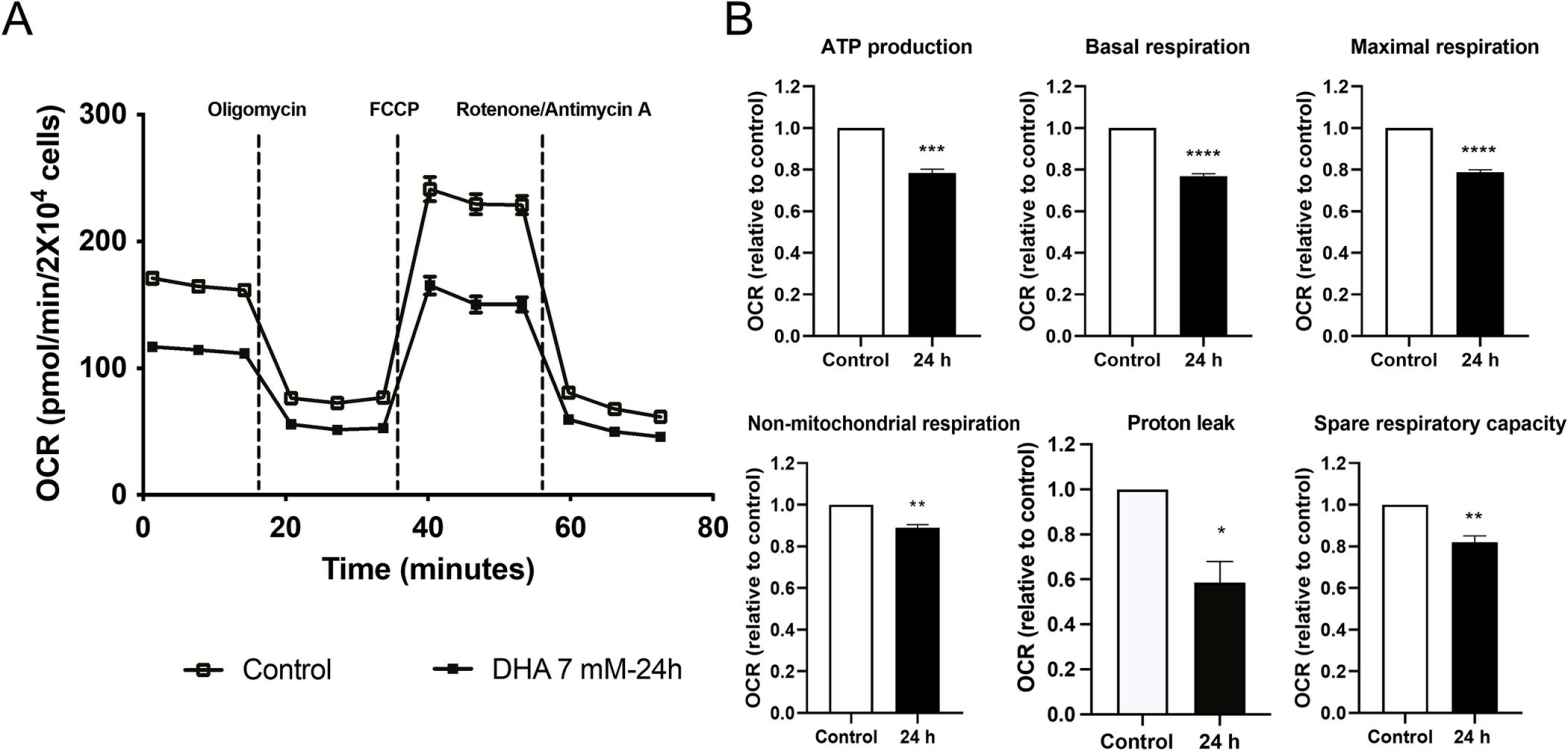
Oxidative phosphorylation is also reduced after 7 mM DHA exposure. (A) Oxygen consumption rate (OCR) decreased at 24 h. (B) All measured parameters showed a significant decrease compared to control cells. The level of statistical significance was marked as follows: *p < 0.05; **p < 0.01; ***p < 0.001; ****p<0.0001.

Given the changes in glycolytic function, we examined oxidative phosphorylation changes using the Seahorse XF Mito Stress Test. A decrease in oxygen consumption rate (OCR) was observed in DHA-dosed cells compared to the control (Fig 7A). All six parameters calculated showed significant decreases compared to untreated HepG3 cells (Fig 7B). A summary of the effects of DHA exposure promoted in Mito Stress Test parameters are in Table 3.

**Table 3:** Flux analysis of oxidative phosphorylation in DHA-exposed HepG3 cells.

Parameter	Effect (7 mM DHA)
**ATP production**	20% decrease
**Basal respiration**	25% decrease
**Maximal respiration**	20% decrease
**Non-mitochondrial respiration**	20% decrease
**Proton leak**	40% decrease
**Spare respiratory capacity**	18% decrease

**Fig 8:**
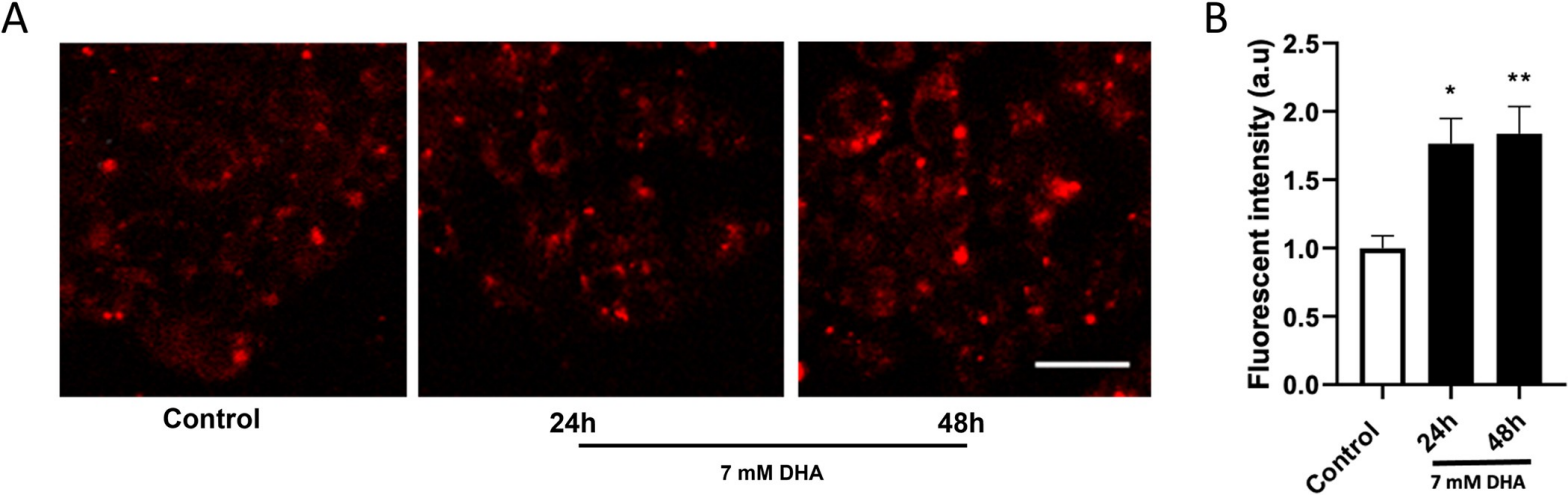
Mitochondrial ROS is increased when cells are exposed to DHA. (A) HepG3 cells were dosed with 7 mM DHA for 24 and 48 h and imaged to evaluate mitochondrial ROS; scale bars 25 μm. (B) A significant increase in fluorescent intensity can be observed at 24 and 48h DHA exposure cells compared to the control. The level of statistical significance was marked as follows: *p < 0.05; **p < 0.01.

Mitochondria are susceptible to damage mediated by ROS that would impair mitochondrial function. We further investigated the accumulation of mitochondrial-specific ROS and superoxide using the MitoSOX assay [28]. Live-cell imaging of mitochondria exposed to 7 mM DHA showed increased superoxide production at 24 and 48 h (Fig 8A). Significant increases in superoxide production by 1.7 to 1.8-fold were calculated at 24 and 48 h after 7 mM DHA exposure in HepG3 cells compared to untreated control cells (Fig 8B).

## Discussion

E-cigarettes have grown exponentially in popularity over the past ten years [29]. Adverse health effects of vaping have been associated with e-juice components and their combustion into reactive molecules [8,30–32]. One overlooked reactive molecule generated from propylene glycol and glycerol combustion is DHA. DHA undergoes a Maillard-like reaction with proteins to produce the brown coloring in sunless tanning agents. More importantly, it is converted to DHAP in cells and rapidly incorporated into metabolic pathways, altering the 3-carbon balance within cells [2,3,15]. Imbalances in 3-carbon metabolites, whether through high fructose exposures or mutations in triosephosphate isomerase (TPI), can cause protein and DNA damage through oxidative stress and methylglyoxal formation, mitochondrial dysfunction, and induce complex disease pathologies [33,34].

While DHA has been considered safe for topical applications, various publications have demonstrated that absorption and systemic distribution of DHA could induce significant cellular and metabolic stress, along with protein and DNA damage [12–14,35–37]. Here, we have extended this work to examine exposure effects in a liver model to demonstrate DHA exposure may have long-term metabolic consequences for even detoxifying organs.

Low millimolar doses of DHA decreased cell growth and viability (Fig 1) and promoted replication stress with a weak cell cycle arrest in G1/S and G2/M (Fig 2). G2/M cell cycle arrest was observed previously in HaCaT keratinocytes dosed with 25 mM DHA [36]. Both cyclin B and cyclin D increases confirmed the weak block at both G1/S and G2/M, suggesting both checkpoints are initiated (Fig 2C). Furthermore, genotoxic damage increases p21 (Fig 2C), a protein related to cell cycle arrest, resulting in DHA-exposed cells decreasing in number.

Apoptotic cell death was confirmed via annexin/PI, but the cell death mechanism appears to be driven through non-classical mechanisms (Figs 3 and 4). Metabolic imbalances signaled through mTOR increase the autophagic marker LC3B and lysosomal stress marker LAMP-1 (Figs 4 and 5) [38]. Increases were also found in autophagy initiating protein, ULK1, driven by mTORC1 found when cells were dosed with 7 mM DHA, consistent with increases in LC3B (S6 Fig) [39]. Previous work found mTOR-independent autophagy in DHA-exposed HEK293T cells, with changes in LC3B cleavage and SIRT1 occurring along with reductions in glycolysis and mitochondrial stress [14]. Here, we observe autophagy leading to caspase-independent apoptosis, consistent with a recent study by Thorburn, which identified crosstalk between autophagy and apoptosis, suggesting the cell death mechanisms switch depending on the current state of the cell [40].

Further evidence of hybrid cell death was found when cells were dosed with chemical inhibitors, chloroquine, inhibiting autophagy, or rapamycin, inhibiting mTOR, thus activating autophagy [26,41]. Similar decreases in viability were found when cells were treated with a combination of DHA and either chloroquine or rapamycin. Immunoblotting of rapamycin shifted the timing of mTOR activation in combination with DHA compared to DHA alone (S5B Fig). Changes in LC3BII alone were also observed with a stronger signal in the DHA and rapamycin combination group (S5B Fig). Chloroquine combined with DHA decreased LC3BII alone compared to DHA alone yet showed no increase in cleavage of the classic caspase-mediated apoptosis proteins, caspase 3 and PARP1 (S6C Fig). These results confirm the role of autophagy in DHA-mediated cell death but still suggest a hybrid between autophagy and non-classical apoptosis mechanisms. In addition, the observed changes in full-length and cleaved cathepsin also point to a hybrid cell death linked to autophagy and apoptosis (Fig 4) as the energetics of the cells are depleted through DHA exposure [42]. Altogether, we have evidence pointing to a hybrid mechanism with the clearest evidence for autophagy and lysosomal stress. However, the apoptotic mechanism could not be fully mapped, though mitochondrial energetics are likely involved in this process.

Increases in full-length caspase 3, cathepsin B, and cytochrome c expression indicate mitochondrial injury [43,44]. We measured increased mitochondrial-specific ROS after DHA exposure, contributing to the reduced oxidative phosphorylation (Figs 7 and 8). Oxidative stress and mitochondrial DNA damage impair the ability of the mitochondria to replace damaged electron transport proteins, hindering all the parameters measured in the Mito stress test, including oxygen consumption, ATP production, and energy demands (Fig 7). DHA depletes the bioenergetic reserve capacity, causing mitochondrial dysfunction and programmed cell death. The reserve capacity is essential for resistance to oxidative stress and supplying ATP demand [45].

While the mitochondrial injury may be expected through increased ROS and even potential flux through glycerol-phosphate shuttling at the mitochondrial membrane, the suppression of glycolysis was unexpected given that DHA is a carbohydrate source and DHAP is a breakdown product of fructose. However, like oxidative phosphorylation, glycolysis and glycolytic capacity significantly decreased (Fig 6). These data demonstrate that DHA exposure alters the cell’s ability to sense and produce energy until it cannot meet its energy demands. Similar effects have been observed in cells exposed to high glucose or fructose where increased 1,3-bisphosphoglycerate reacts with lysine residues of nearby proteins to form 3-phosphoglycerate-lysine (pgK) conjugates. The pgK post-translational modification is enriched around the active sites of glycolytic enzymes, significantly reducing their enzymatic activities [46]. Glycolytic enzymes are present but cannot perform their functions when modified in this manner. While it is unclear if DHA produces similar effects, it is likely, given the similarities between DHA and fructose exposures, the fact DHA converts to DHAP, a breakdown product of fructose, and the clear depression in glycolysis observed after DHA exposures, warranting further investigation (Fig 6) [14,34].

Nutrient and metabolic changes have also been associated with the mTOR pathway, altered by DHA exposure in the HepG3 cells (Fig 5) [27]. The results here are consistent with a recent paper by Orozco et al., where DHA supplementation increased mTORC1 signaling in selective aldolase (ALDO) and TPI HEK293T knockouts. mTORC1 is involved in an AMPK-independent pathway where DHAP acts as a glucose-sensing metabolite. Orozco et al. link cellular DHAP levels in the cells with nutrient availability [15]. Our results demonstrate DHA exposure in HepG3 cells alters both mTORC1 and mTORC2 complexes on fast and slow time scales (Fig 5). Suppression of the nutrient-sensing pathway, AMPKα, was also found despite the novel DHAP and mTORC1 pathway being AMPK-independent. The trend correlates with elevated glucose concentrations and insulin resistance in nutrient-excess environments [47–49].

Altogether, the data for HepG3 suggest that the presence of DHA is manipulating nutrient balance, and nutrient sensing is being adapted or possibly subverted. This is particularly interesting because we see a decrease in both major energetic pathways. Often antagonistic roles of glycolysis and oxidative phosphorylation are elicited in cells. Yet, DHA appears to overwhelm metabolic pathways, possibly through post-translational modifications, signaling changes, altered cofactor balance, or a combination of these mechanisms promoting metabolic imbalances [14,46,50]. Therefore, despite having a carbohydrate source that should readily be incorporated into the glycolysis pathway, we see reduced energetic flux, metabolic rewiring, and mitochondrial stress, promoting cell death and lysosomal stress after DHA exposure.

These findings demonstrate the need to understand the systemic exposure effects of DHA and how they may enhance or contribute to the adverse effects observed from e-cigarette exposures. While the low millimolar IC_90_ doses here exceed current estimates for one-time vaping session exposures (0.14 to 600 μM), it may be relevant to repeated or chronic exposures to DHA from e-cigarettes. As we obtain more accurate information about e-cigarette use and exposure, we can better understand DHA exposure levels from vaping and model interactions with other combustion ingredients. It is important to note that DHA is produced by the e-juice mobilant and is not a low part per billion flavoring additive. Inhalation and absorption exposures to DHA require further study to understand the long-term health effects for vapers and individuals exposed to secondhand vaping.

## Supporting information

S1 Fig Cell growth and viability decrease confirmed by growth inhibition in HepG3. Cells were exposed to a range of DHA doses and counted after 5 days. The graph displays the percentage of cells relative to control. An IC_50_ of 1.5 mM ± 0.98 and an IC_90_ of 4.6 mM ± 0.72 were calculated.
(TIF)

S2 Fig Genotoxic effects induced by DHA. (A) HepG3 cells were dosed with 7mM DHA for the corresponding time points, and strand break analysis was performed with γH2AX staining. (B) A significant increase in intensity was observed starting at 24 h and continued until 96h. The statistical significance level was marked as ****p<0.0001.
(TIF)

S3 Fig Cell death markers probed in DHA-dosed cells. (A and C) Apoptotic markers, caspase 1 and caspase 2 were probed to define the cell death mechanism. No changes were observed nor calculated in either caspase 1 or 2 when cells were dosed with DHA compared to untreated controls. (B) Caspase 3 was also probed starting at 24 h, and no changes were found. Cathepsin L was probed, and no protein expression levels were observed at any time points.
(TIF)

S4 Fig LC3BII intensity was calculated in addition to the ratio calculated in Fig 4. A significant increase was found in cells dosed with 96 h DHA. Three biological replicates were quantified, and the significance level is marked as follows: ***p < 0.001.
(TIF)

S5 Fig Growth inhibition in cells dosed with rapamycin and DHA. (A) Cells were exposed to DHA, rapamycin, or a combination of rapamycin and DHA and counted after 5 days. The graph displays the percentage survival of cells relative to control. (B) Immunoblotting of associated proteins using a select dose of rapamycin alone and in combination with DHA. A similar increase in LC3BII after DHA and rapamycin alone dosing was observed and higher expression after combination treatment. mTOR protein levels increased at 72 h in DHA alone, while the combination treatment increased at 24 h. (C) Apoptotic markers were probed using a select dose of rapamycin alone and in combination with DHA which found no change between caspase 3 and PARP1 as well as no cleavage. Three biological replicates were quantified, and the significance level is marked as follows compared to the control: *p < 0.05, **p < 0.01, ***p < 0.001, ****p < 0.0001. The significance level is marked as ^##^*p* < 0.01 when compared to 1.25 mM DHA.
(TIF)

S6 Fig Growth inhibition in cells treated with chloroquine and DHA. (A) Cells were exposed to DHA, chloroquine, or a combination of chloroquine and DHA and counted after 5 days. The graph displays the percentage survival of cells relative to control. (B) Immunoblotting autophagic cell death markers using a select dose of chloroquine alone and in combination with DHA. LC3BII was found to reduce after DHA and chloroquine treatment compared to DHA and return to control level. (C) Immunoblotting of apoptotic using a select dose of chloroquine alone and in combination with DHA. No change in apoptotic markers caspase 3 and PARP1 were observed in combination treatment. Three biological replicates were quantified, and the significance level is marked as follows compared to control: *p < 0.05, **p < 0.01, ****p < 0.0001. The significance level is marked as ^##^*p* < 0.01 when compared to 1.25 mM DHA.
(TIF)

S7 Fig Cytochrome c localization and intensity were evaluated through immunofluorescence. (A) An increase was found in cells dosed with DHA starting at 48 h until 96 h. Cytochrome c is found in the mitochondrial compartment only. (B) Cytochrome c intensity was quantified, where a 2-fold increase was calculated in cells dosed with 96 h DHA. Significance is indicated as **p < 0.01.
(TIF)

S8 Fig Autophagy mediated through mTOR-specific signaling was confirmed. Changes in phospho- and total ULK1 protein levels were probed to validate autophagic cell death through mTOR in cells dosed with DHA 24–96 h. One biological replicate was conducted for the immunoblot.
(TIF)

S9 Fig The downstream protein of mTORC1, 4E-BP1 was evaluated to confirm mTOR-specific signaling. (A and B) Total 4E-BP1 started to decrease at 4 h and significantly decreased at 24 h while phosphor-4EBP1 levels significantly decreased starting at 1 h and continued until 24 h. Significance is marked as shown: *p < 0.05; **p < 0.01.
(TIF)

S10 Fig ATP levels show transient changes with exposure to DHA. A slight decrease in ATP levels was found at 1 and 24 h while remaining constant at 4 h in cells exposed to 7 mM DHA. Expression levels are expressed as a percentage of control.
(TIF)

S1 File (PDF)

S1 Graphical abstract (TIF)
