# Supplementary figures and images for: Dihydroxyacetone suppresses mTOR nutrient signaling and induces mitochondrial stress in liver cells
Source: PLoS One. 2022 Dec 6;17(12):e0278516. doi: 10.1371/journal.pone.0278516 (PMC9725129; doi:10.1371/journal.pone.0278516)

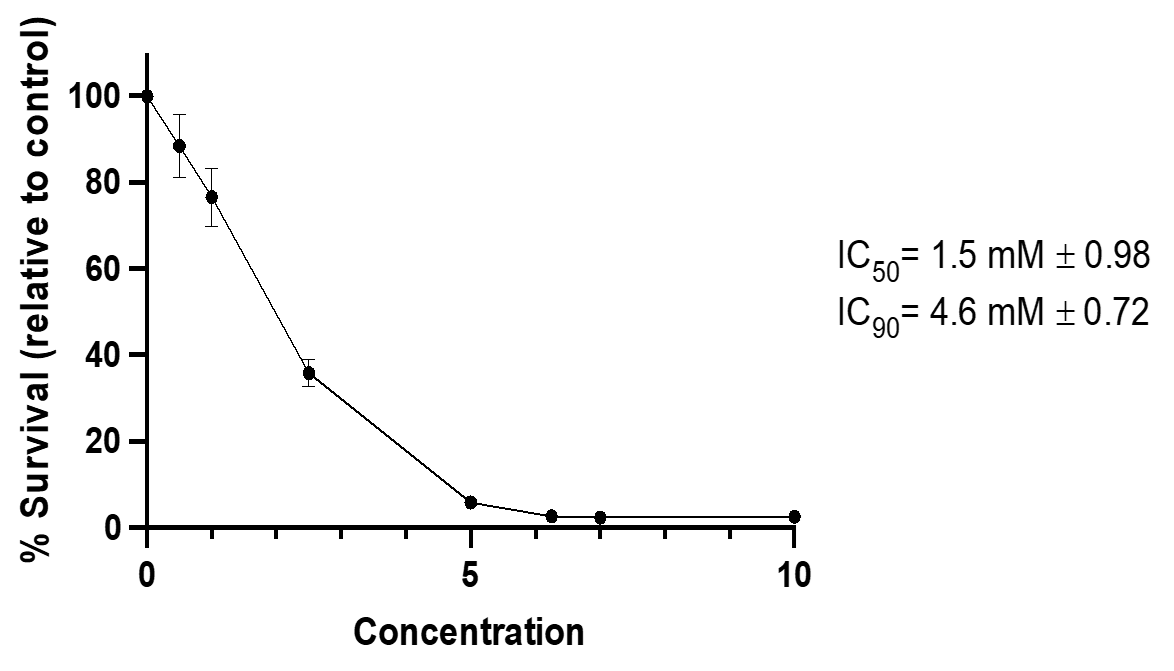

Supplement: S1 Fig — Cells were exposed to a range of DHA doses and counted after 5 days. The graph displays the percentage of cells relative to control. An IC50 of 1.5 mM ± 0.98 and an IC90 of 4.6 mM ± 0.72 were calculated. (TIF) [file pone.0278516.s001.tif]

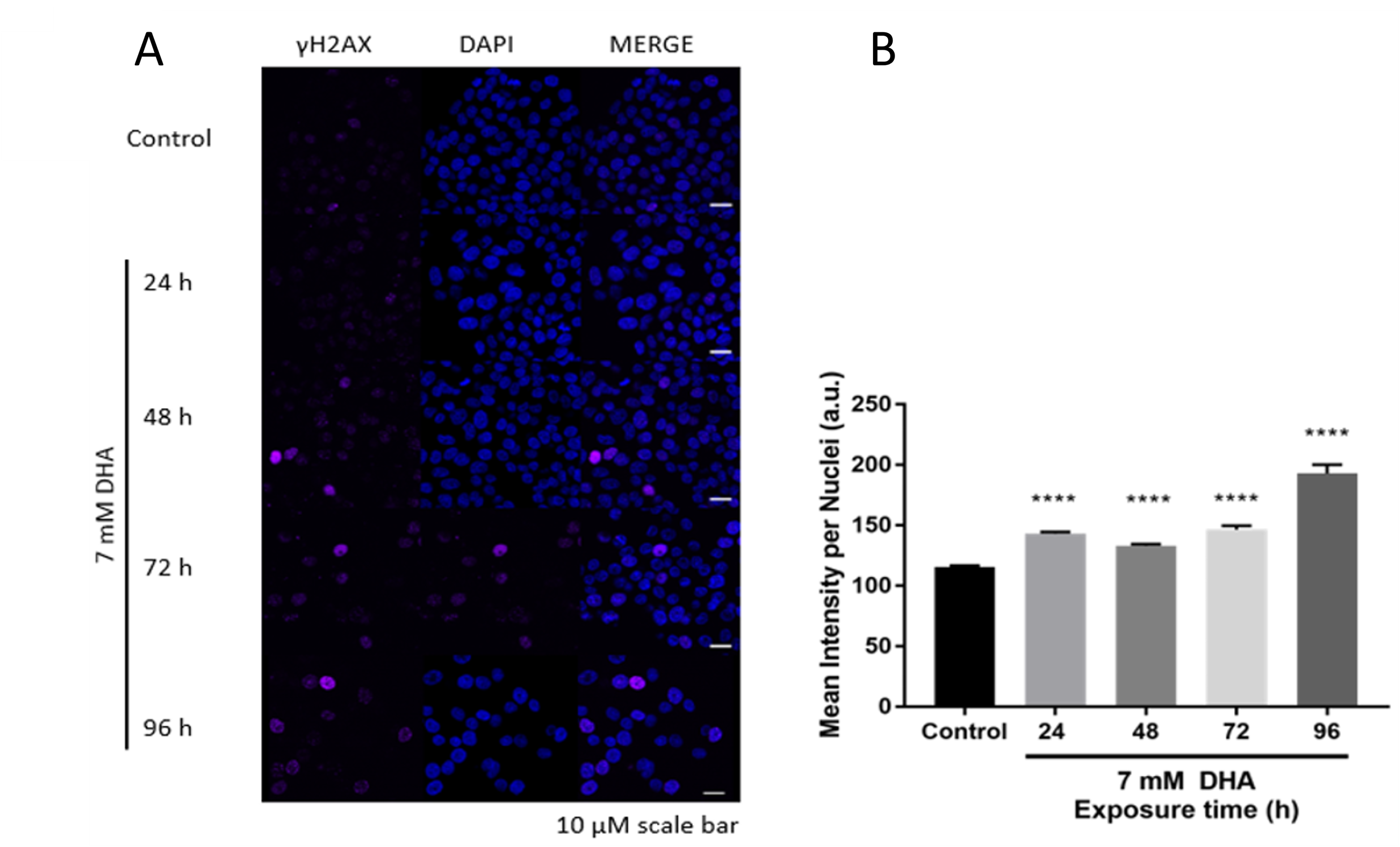

Supplement: S2 Fig — (A) HepG3 cells were dosed with 7mM DHA for the corresponding time points, and strand break analysis was performed with γH2AX staining. (B) A significant increase in intensity was observed starting at 24 h and continued until 96h. The statistical significance level was marked as ****p<0.0001. (TIF) [file pone.0278516.s002.tif]

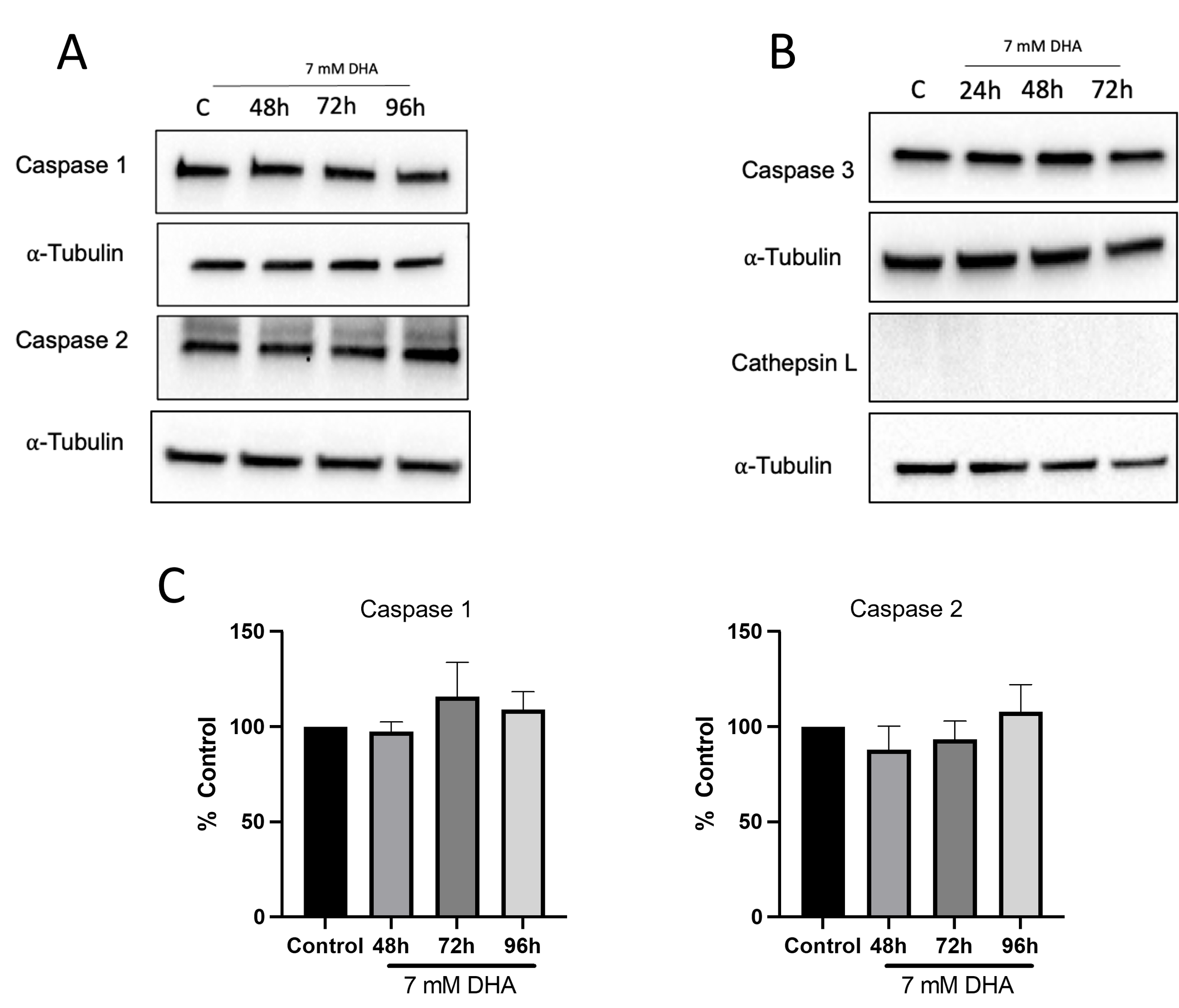

Supplement: S3 Fig — (A and C) Apoptotic markers, caspase 1 and caspase 2 were probed to define the cell death mechanism. No changes were observed nor calculated in either caspase 1 or 2 when cells were dosed with DHA compared to untreated controls. (B) Caspase 3 was also probed starting at 24 h, and no changes were found. Cathepsin L was probed, and no protein expression levels were observed at any time points. (TIF) [file pone.0278516.s003.tif]

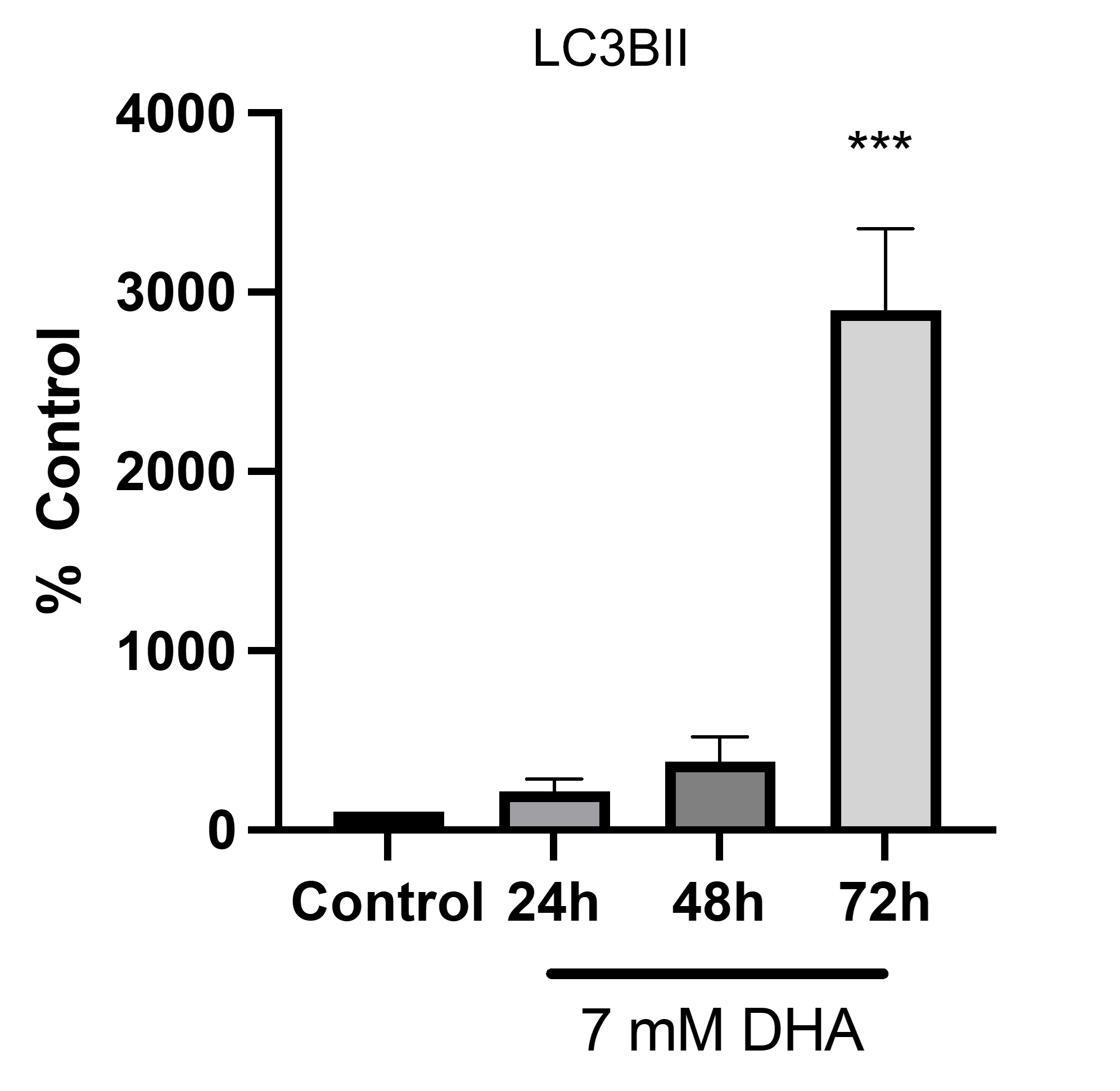

Supplement: S4 Fig — A significant increase was found in cells dosed with 96 h DHA. Three biological replicates were quantified, and the significance level is marked as follows: ***p < 0.001. (TIF) [file pone.0278516.s004.tif]

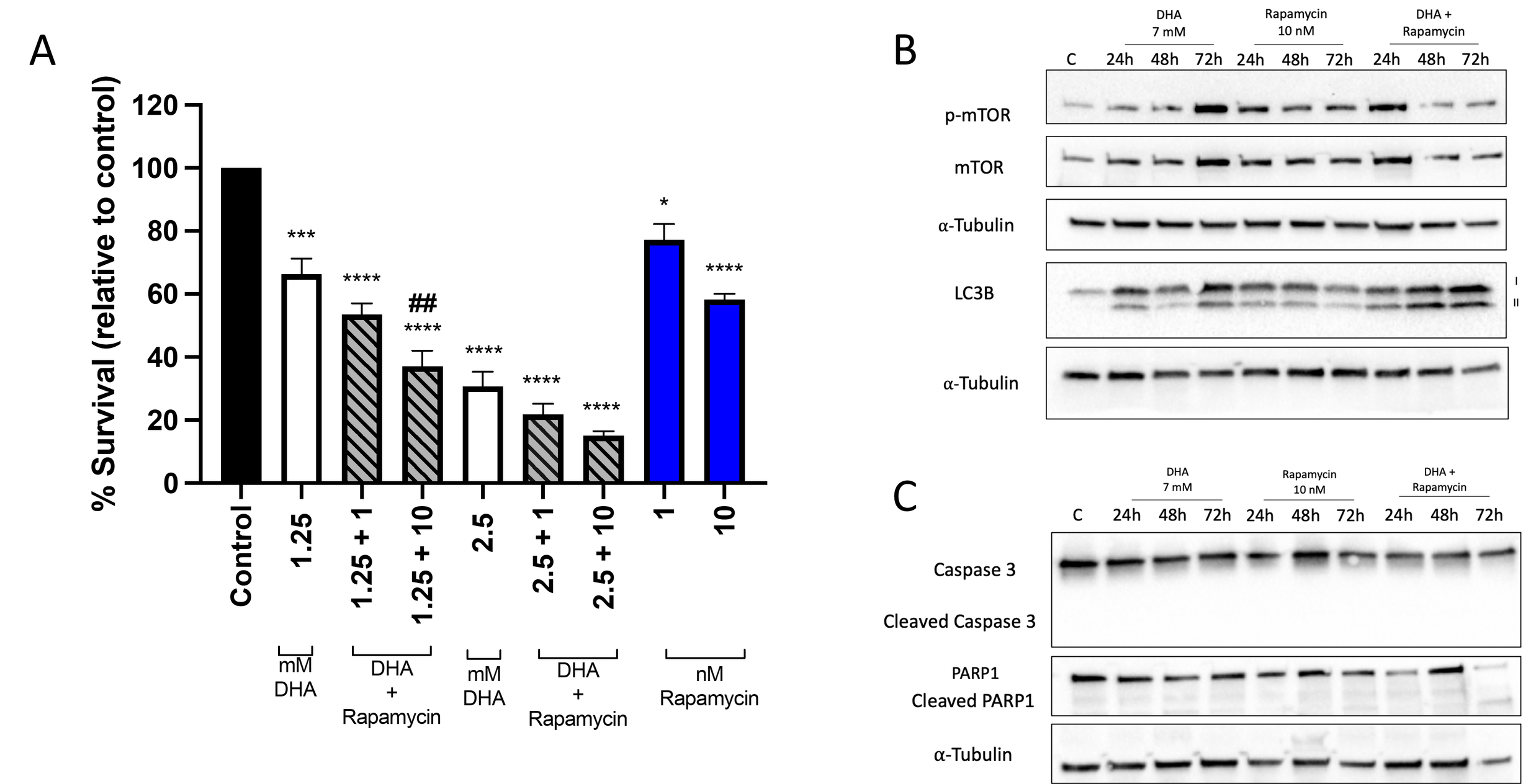

Supplement: S5 Fig — (A) Cells were exposed to DHA, rapamycin, or a combination of rapamycin and DHA and counted after 5 days. The graph displays the percentage survival of cells relative to control. (B) Immunoblotting of associated proteins using a select dose of rapamycin alone and in combination with DHA. A similar increase in LC3BII after DHA and rapamycin alone dosing was observed and higher expression after combination treatment. mTOR protein levels increased at 72 h in DHA alone, while the combination treatment increased at 24 h. (C) Apoptotic markers were probed using a select dose of rapamycin alone and in combination with DHA which found no change between caspase 3 and PARP1 as well as no cleavage. Three biological replicates were quantified, and the significance level is marked as follows compared to the control: *p < 0.05, **p < 0.01, ***p < 0.001, ****p < 0.0001. The significance level is marked as ##p < 0.01 when compared to 1.25 mM DHA. (TIF) [file pone.0278516.s005.tif]

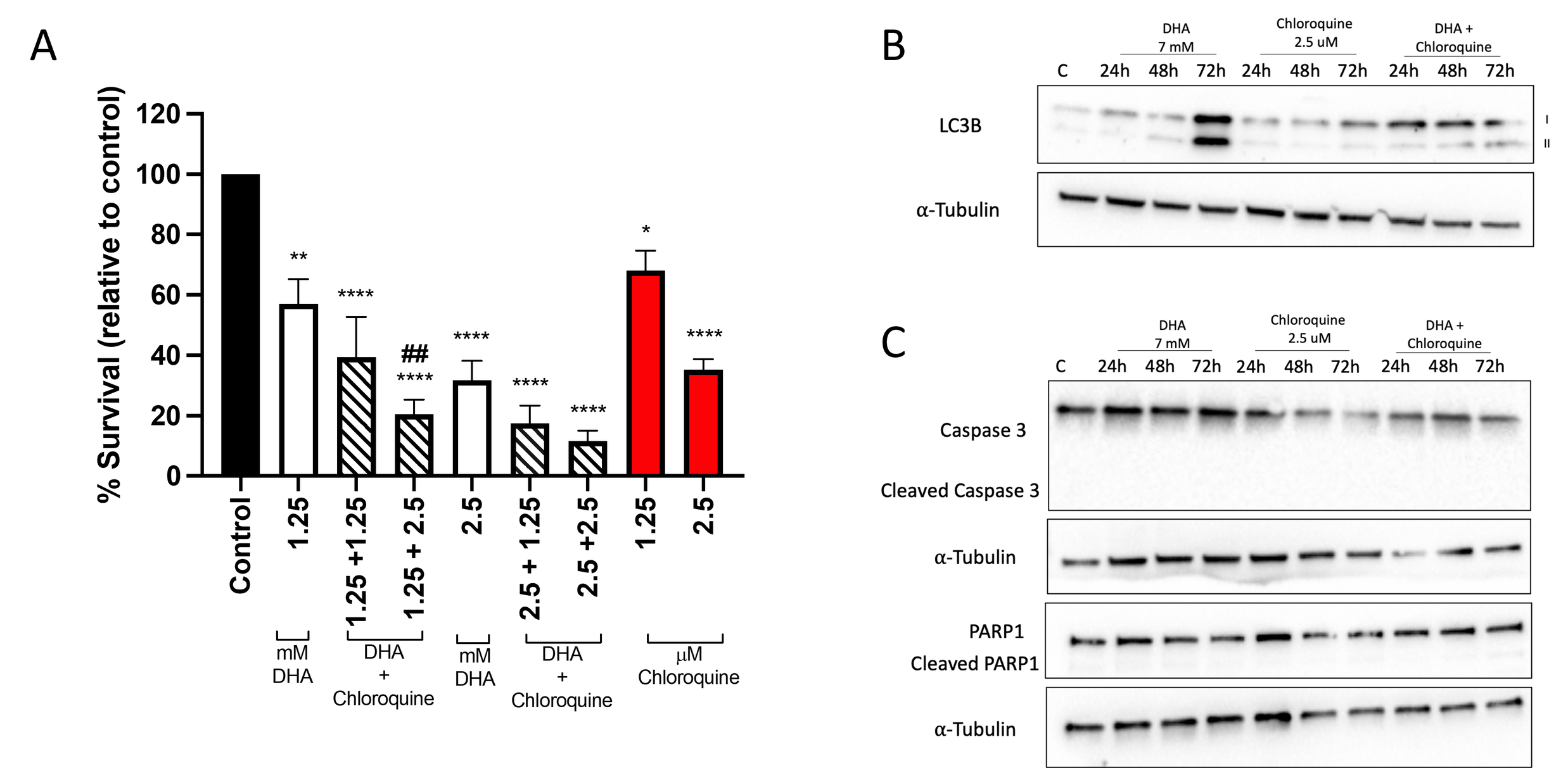

Supplement: S6 Fig — (A) Cells were exposed to DHA, chloroquine, or a combination of chloroquine and DHA and counted after 5 days. The graph displays the percentage survival of cells relative to control. (B) Immunoblotting autophagic cell death markers using a select dose of chloroquine alone and in combination with DHA. LC3BII was found to reduce after DHA and chloroquine treatment compared to DHA and return to control level. (C) Immunoblotting of apoptotic using a select dose of chloroquine alone and in combination with DHA. No change in apoptotic markers caspase 3 and PARP1 were observed in combination treatment. Three biological replicates were quantified, and the significance level is marked as follows compared to control: *p < 0.05, **p < 0.01, ****p < 0.0001. The significance level is marked as ##p < 0.01 when compared to 1.25 mM DHA. (TIF) [file pone.0278516.s006.tif]

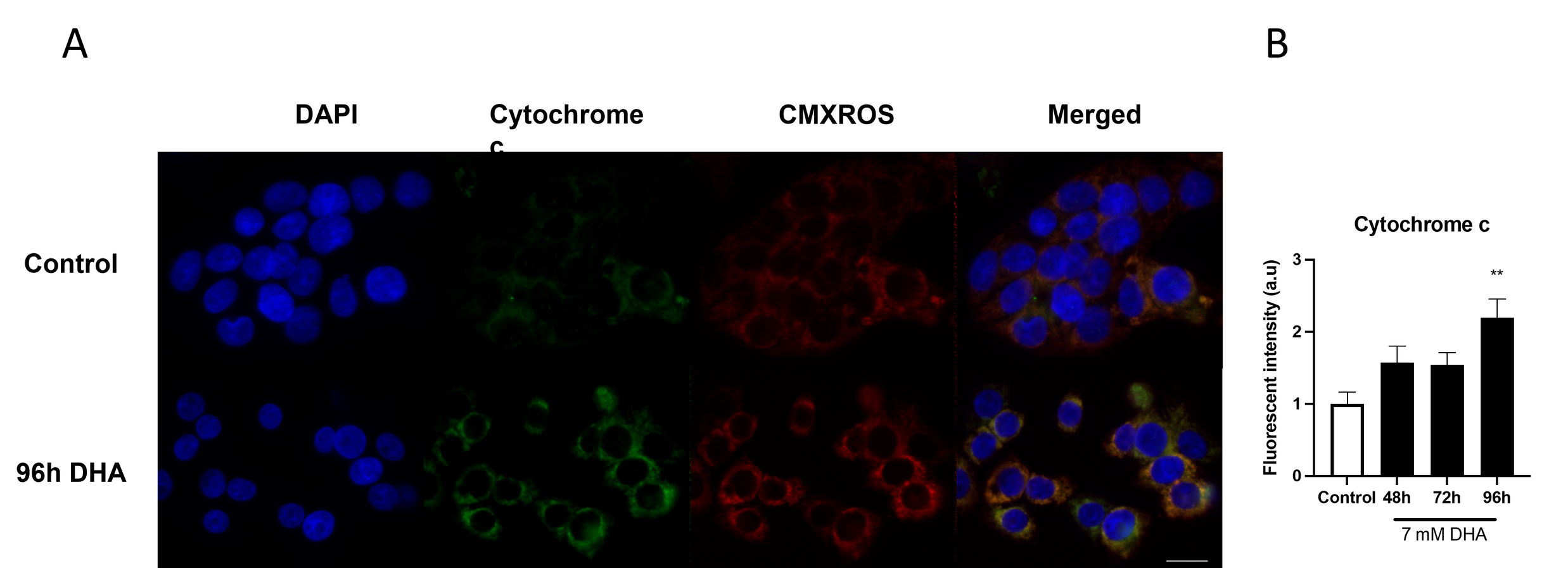

Supplement: S7 Fig — (A) An increase was found in cells dosed with DHA starting at 48 h until 96 h. Cytochrome c is found in the mitochondrial compartment only. (B) Cytochrome c intensity was quantified, where a 2-fold increase was calculated in cells dosed with 96 h DHA. Significance is indicated as **p < 0.01. (TIF) [file pone.0278516.s007.tif]

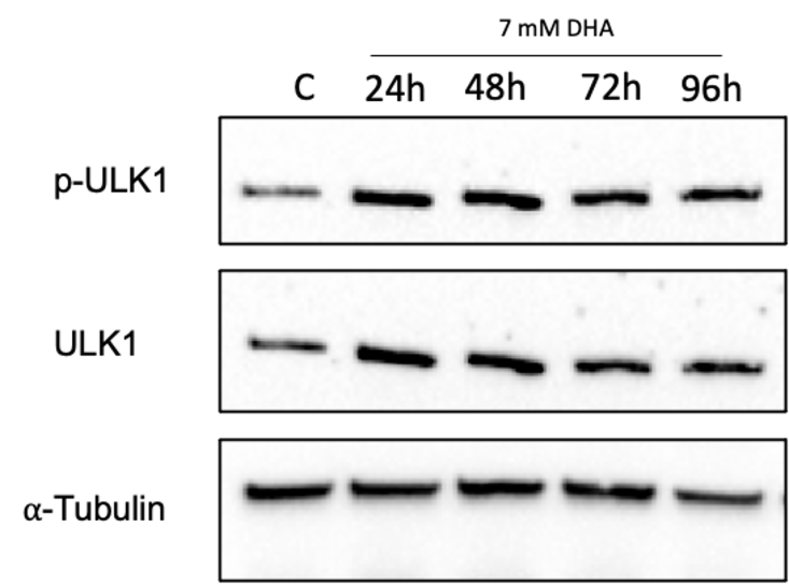

Supplement: S8 Fig — Changes in phospho- and total ULK1 protein levels were probed to validate autophagic cell death through mTOR in cells dosed with DHA 24–96 h. One biological replicate was conducted for the immunoblot. (TIF) [file pone.0278516.s008.tif]

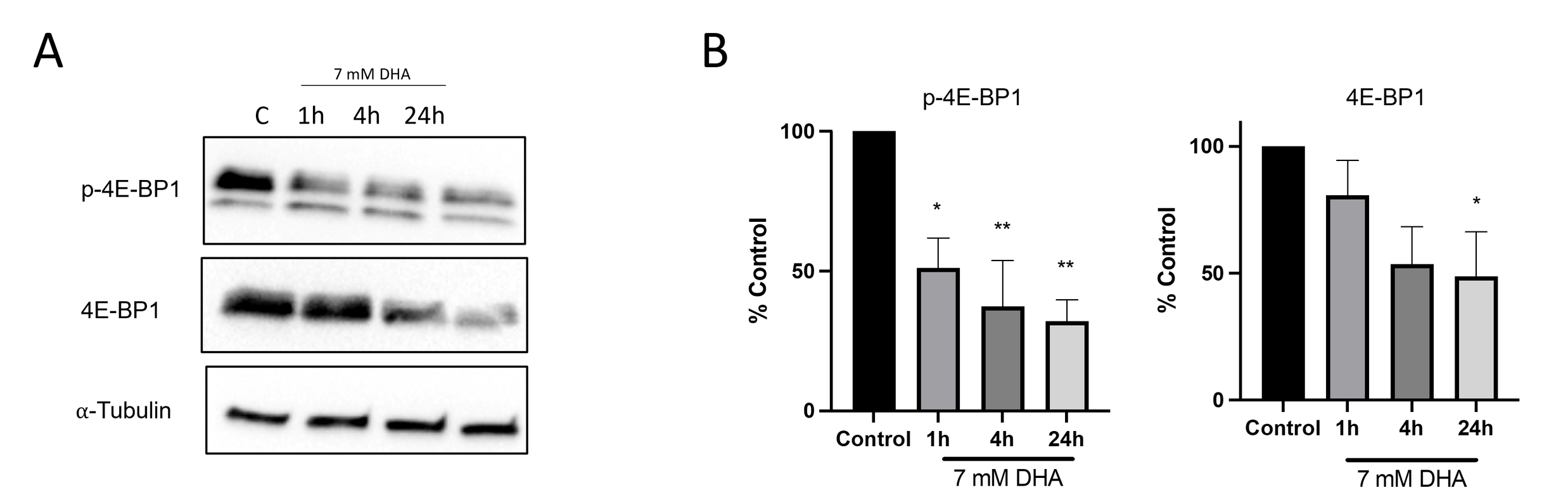

Supplement: S9 Fig — (A and B) Total 4E-BP1 started to decrease at 4 h and significantly decreased at 24 h while phosphor-4EBP1 levels significantly decreased starting at 1 h and continued until 24 h. Significance is marked as shown: *p < 0.05; **p < 0.01. (TIF) [file pone.0278516.s009.tif]

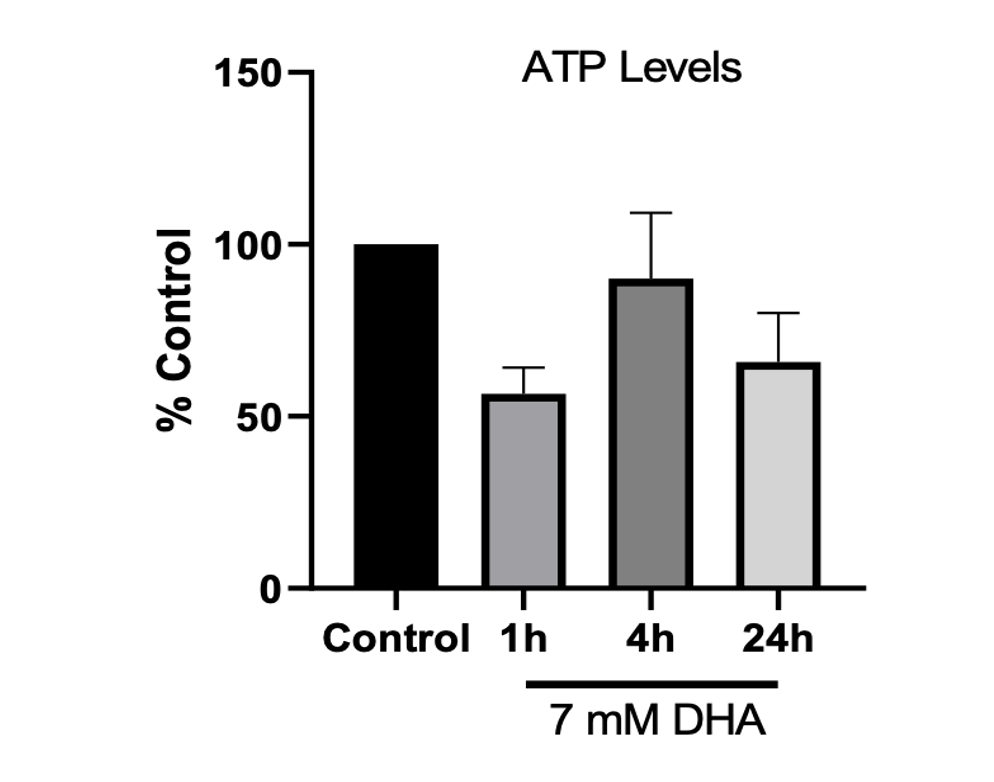

Supplement: S10 Fig — A slight decrease in ATP levels was found at 1 and 24 h while remaining constant at 4 h in cells exposed to 7 mM DHA. Expression levels are expressed as a percentage of control. (TIF) [file pone.0278516.s010.tif]

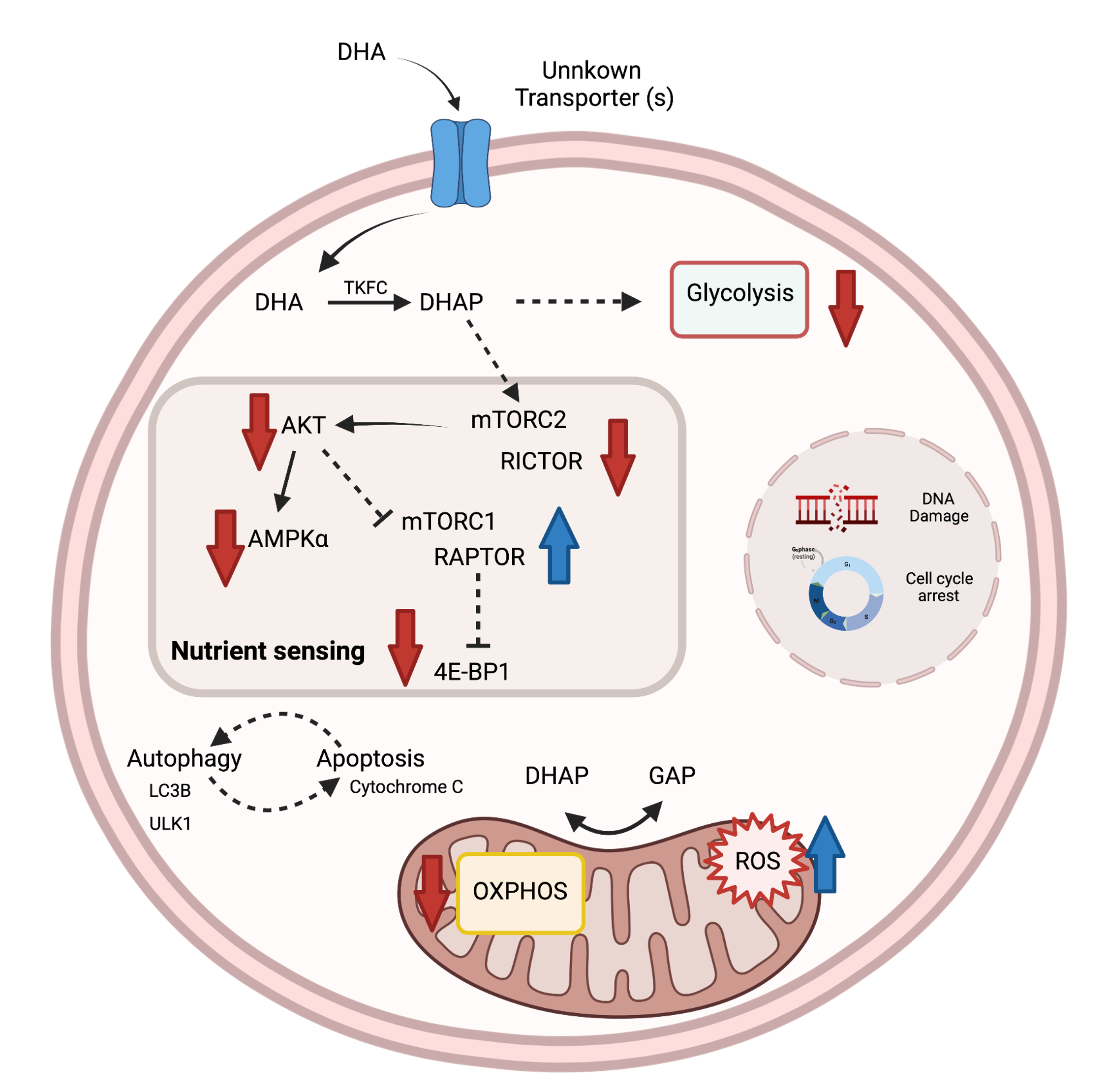

Supplement: S1 Graphical abstract — (TIF) [file pone.0278516.s012.tif]
