# Supplementary material for: Dihydroxyacetone suppresses mTOR nutrient signaling and induces mitochondrial stress in liver cells
Source: PLoS One. 2022 Dec 6;17(12):e0278516. doi: 10.1371/journal.pone.0278516 (PMC9725129; doi:10.1371/journal.pone.0278516)

Figure 2C
Probing: Cyclin B1 (12231)
Cell Signaling Technologies

Loading order:
Lane 1: Control at 72 h
Lane 2: 24h 7mM DHA
Lane 3: 48h 7mM DHA
Lane 4: 72 h 7mM DHA

Imaged with Bio-Rad ChemiDoc XRS
Imaging system
Chemi Hi Sensitivity setting

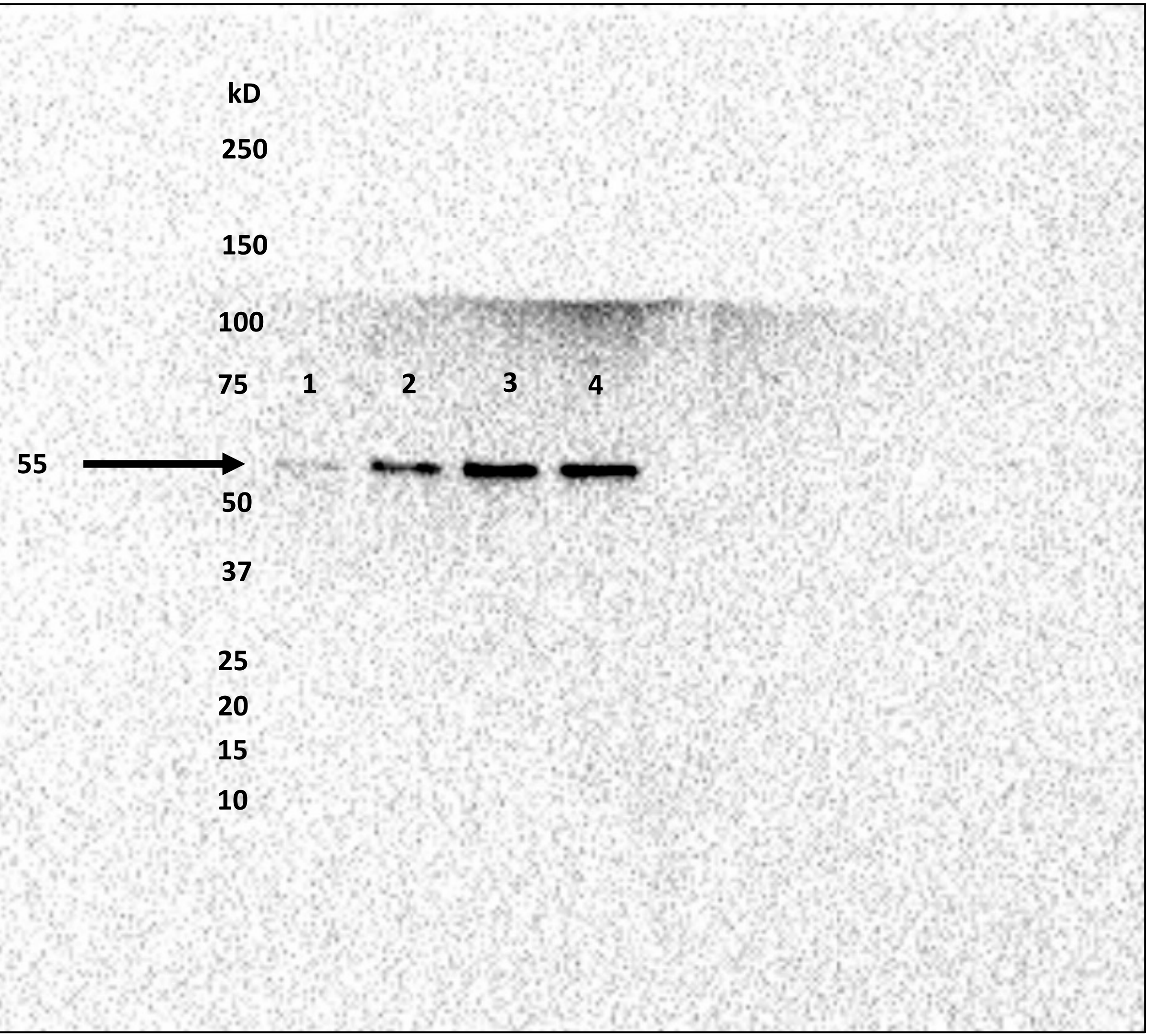

Figure 2C
Probing: Cyclin D1 (8396)
Santa Cruz Biotechnology

Loading order:
Lane 1: Control at 72 h
Lane 2: 24h 7mM DHA
Lane 3: 48h 7mM DHA
Lane 4: 72 h 7mM DHA

Imaged with Bio-Rad ChemiDoc
XRS Imaging system
Chemi Hi Sensitivity setting

Note: Non-specific bands from
previous antibody probing

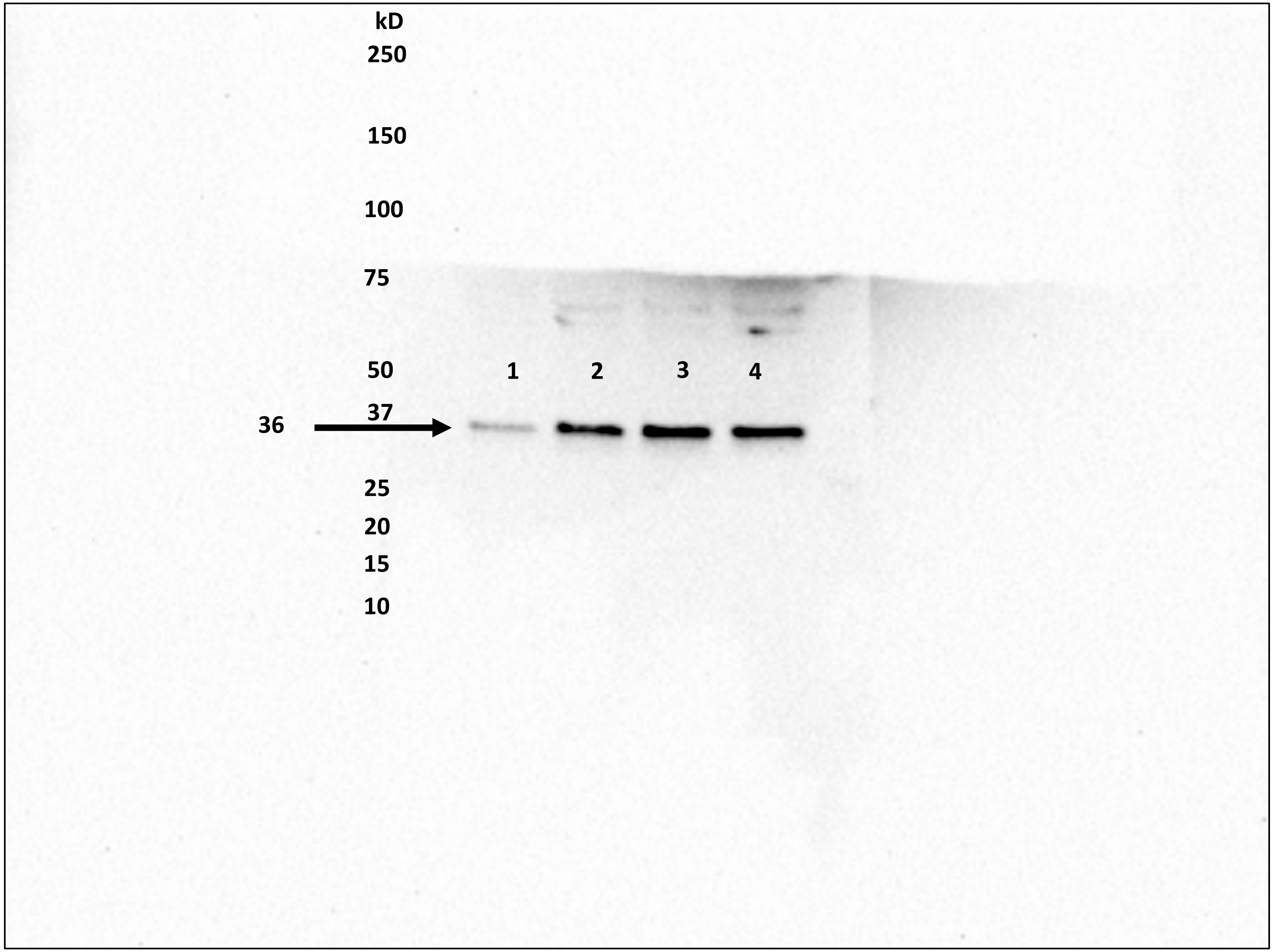

Figure 2C
Probing: p21 (397)
Santa Cruz Biotechnologies

Loading order:
Lane 1: Control at 72 h
Lane 2: 24h 7mM DHA
Lane 3: 48h 7mM DHA
Lane 4: 72 h 7mM DHA

Imaged with Bio-Rad ChemiDoc XRS
Imaging system
Chemi Hi Sensitivity setting

Note: Non-specific bands from previous
antibody probing

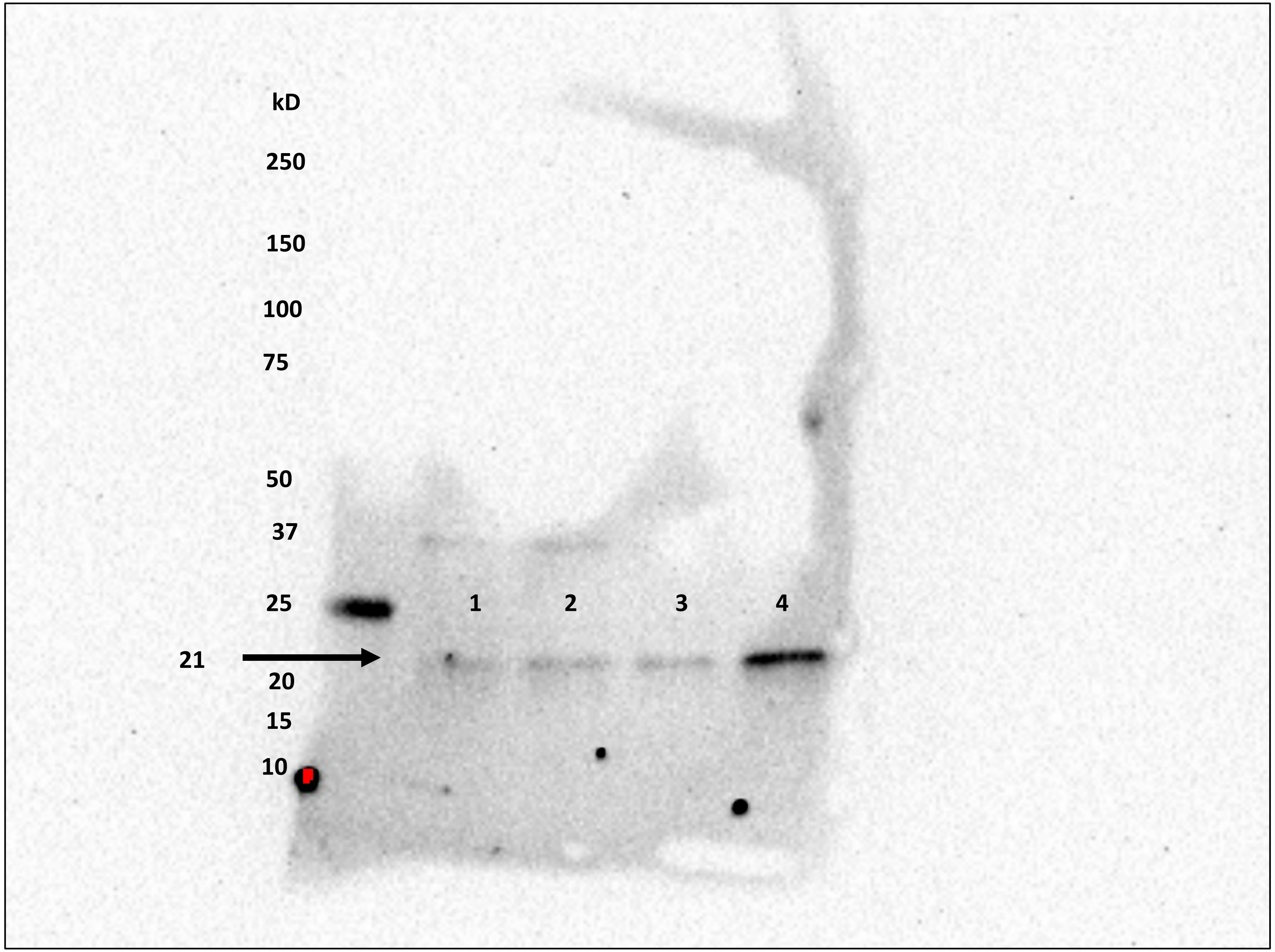

Figure 2C
Probing: α-Tubulin (T9026)
Millipore Sigma

Loading order:
Lane 1: Control at 72 h
Lane 2: 24h 7mM DHA
Lane 3: 48h 7mM DHA
Lane 4: 72 h 7mM DHA

Imaged with Bio-Rad ChemiDoc XRS
Imaging system
Chemi Hi Sensitivity setting

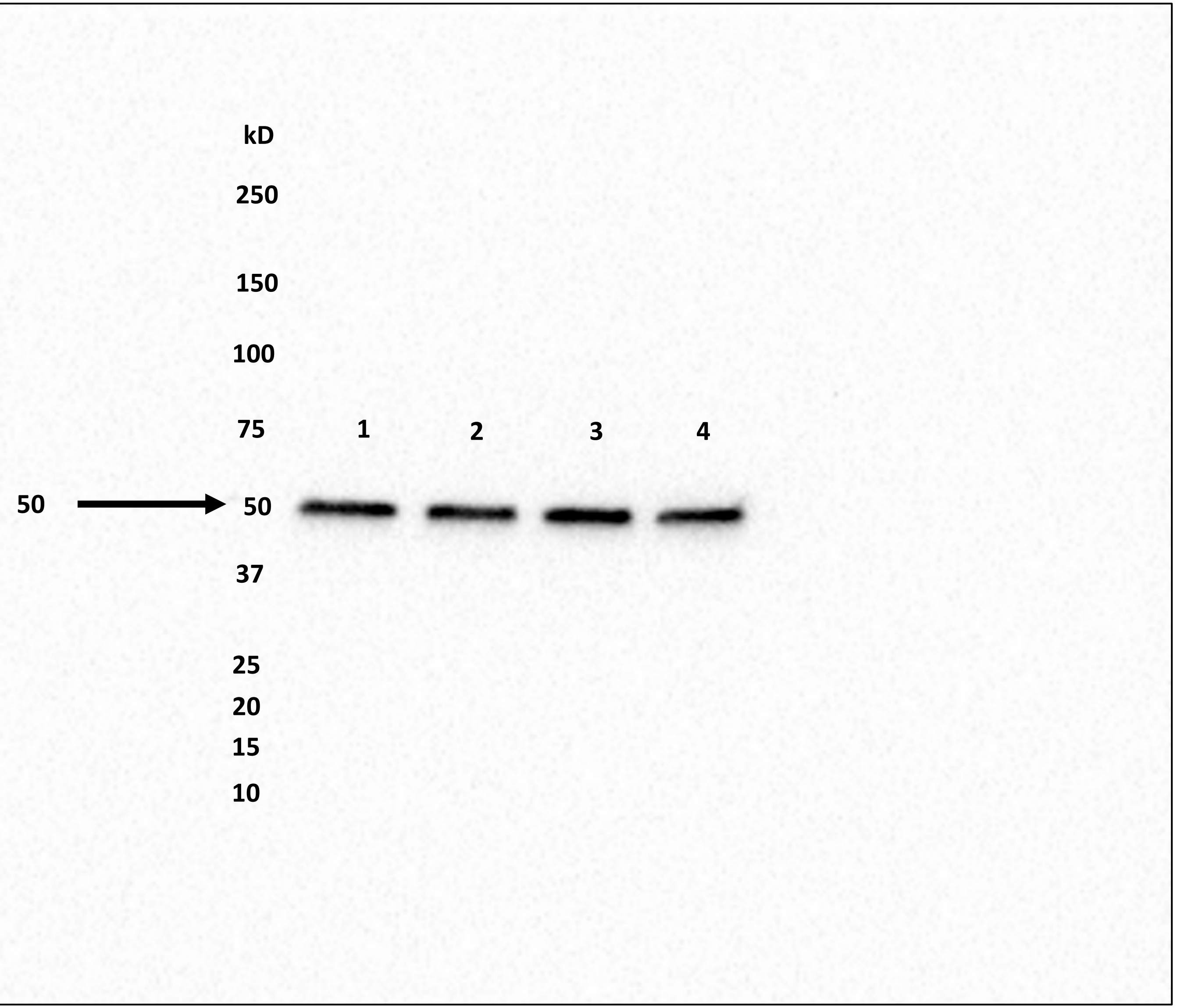

Figure 4A
Probing: Caspase-3 (GTX13585)
Genetex

Loading order:
Lane 1: Control at 96 h
Lane 2: 24h 7mM DHA
Lane 3: 48h 7mM DHA
Lane 4: 72 h 7mM DHA
Lane 5: 96 h 7mM DHA
Lane 6: 24 h 1 μM μCPT
Lane 7: 48h 1 μM μCPT

Imaged with Bio-Rad ChemiDoc XRS
Imaging system
Chemi Hi Sensitivity setting

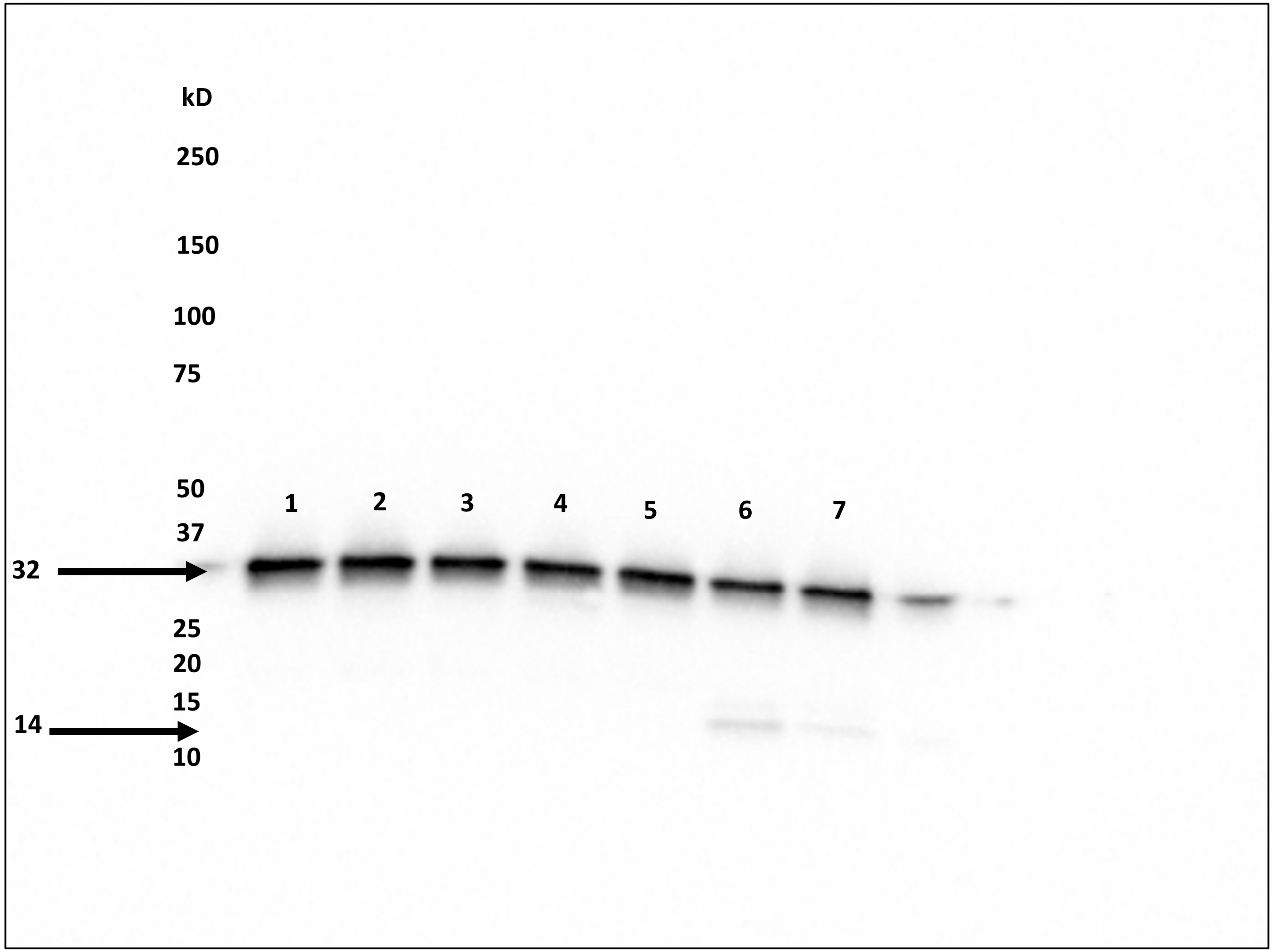

Figure 4A
Probing: PARP-1 (556494)
BD Biosciences

Loading order:
Lane 1: Control at 96 h
Lane 2: 24h 7mM DHA
Lane 3: 48h 7mM DHA
Lane 4: 72 h 7mM DHA
Lane 5: 96 h 7mM DHA
Lane 6: 24 h 1 μM μCPT
Lane 7: 48h 1 μM μCPT

Imaged with Bio-Rad ChemiDoc XRS
Imaging system
Chemi Hi Sensitivity setting

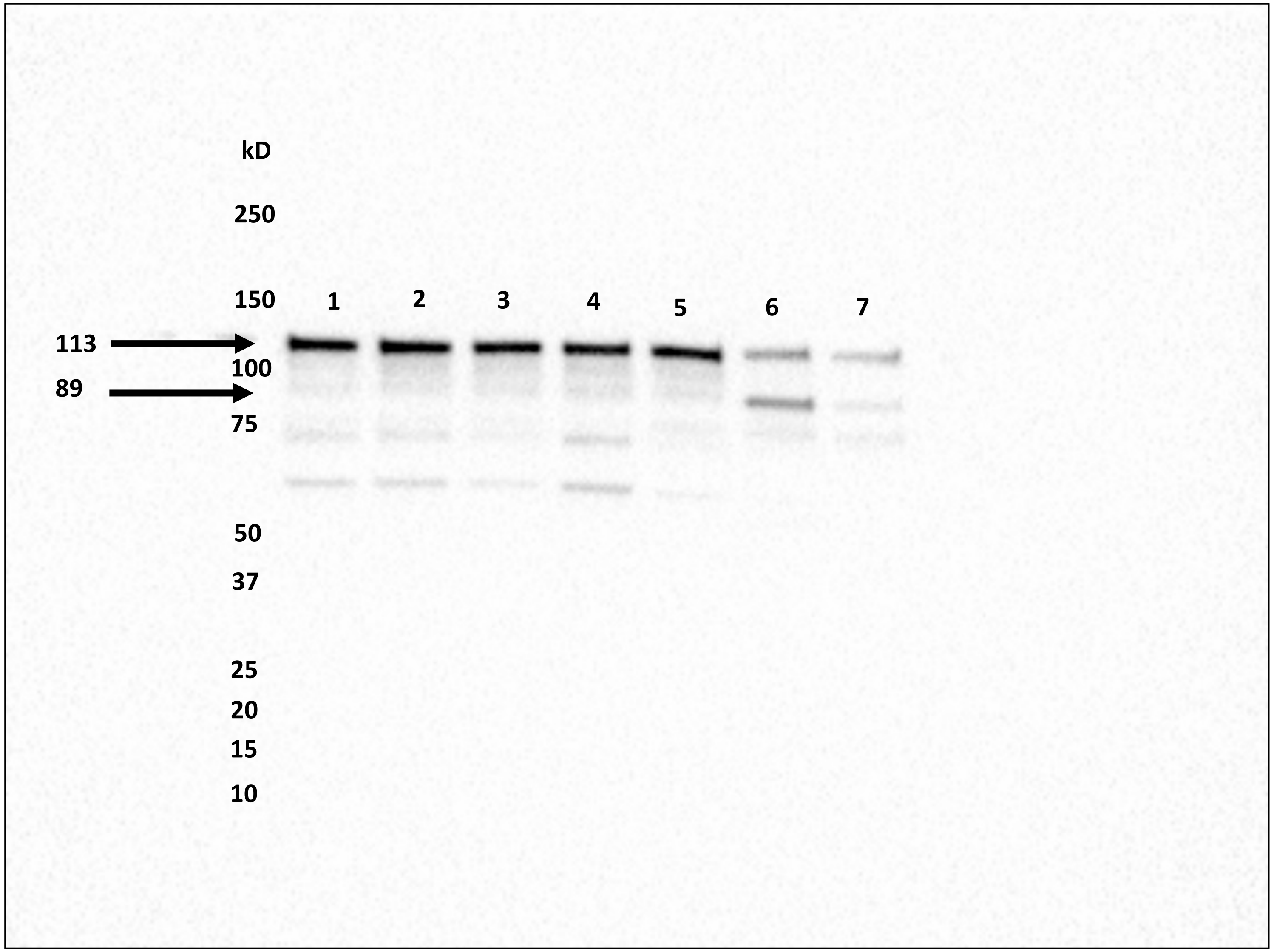

Figure 4A
Probing: α-Tubulin (T9026)
Millipore Sigma

Loading order:
Lane 1: Control at 96 h
Lane 2: 24h 7mM DHA
Lane 3: 48h 7mM DHA
Lane 4: 72 h 7mM DHA
Lane 5: 96 h 7mM DHA
Lane 6: 24 h 1 µM µCPT
Lane 7: 48h 1 µM µCPT

Imaged with Bio-Rad ChemiDoc XRS
Imaging system
Chemi Hi Sensitivity setting

50

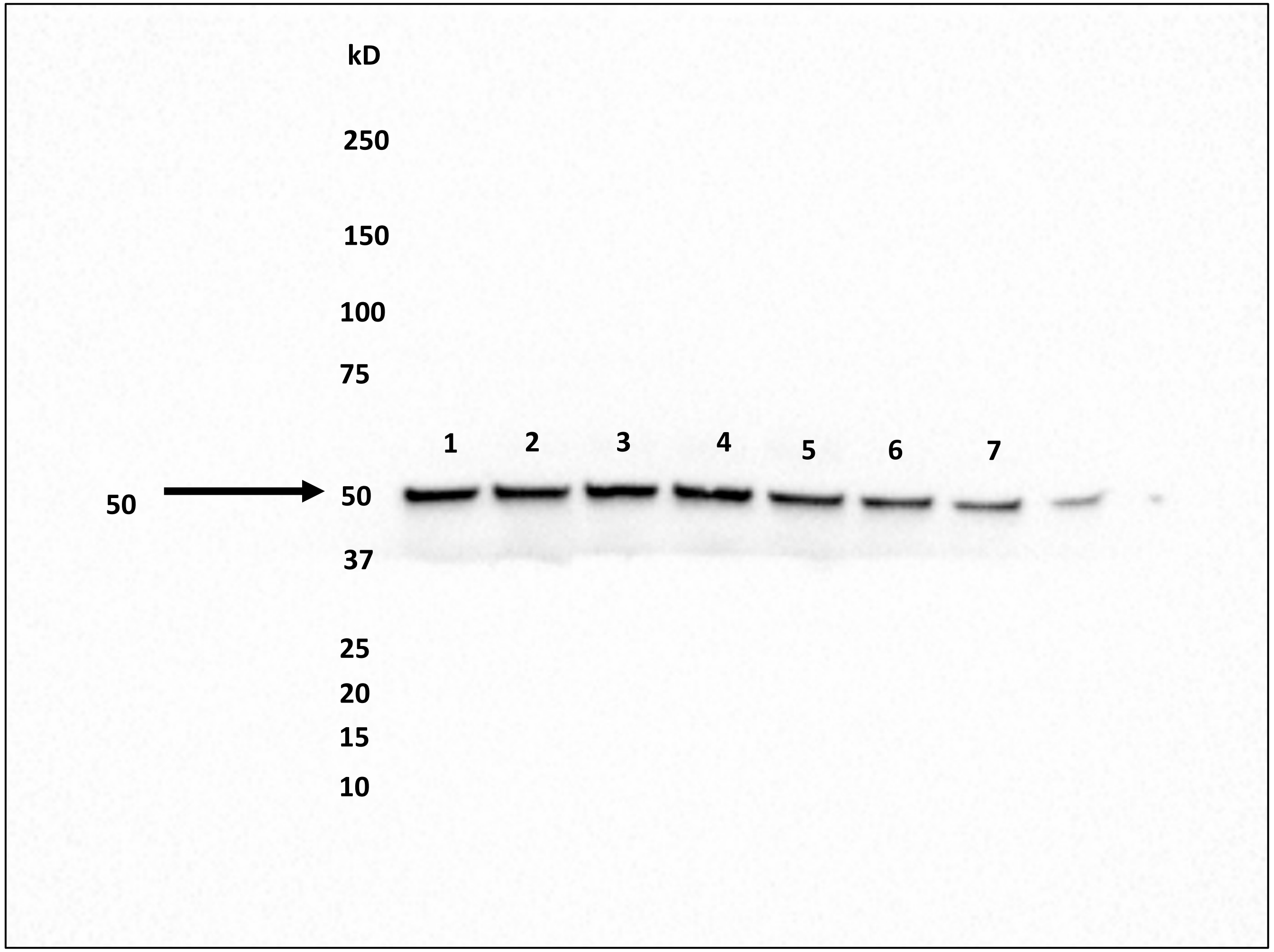

Figure 4B
Probing: LC3B (PA1-46286)
Life Technologies

Loading order:
Lane 1: Control at 72 h
Lane 2: 24h 7mM DHA
Lane 3: 48h 7mM DHA
Lane 4: 72 h 7mM DHA

Imaged with Bio-Rad ChemiDoc XRS
Imaging system
Chemi Hi Sensitivity setting

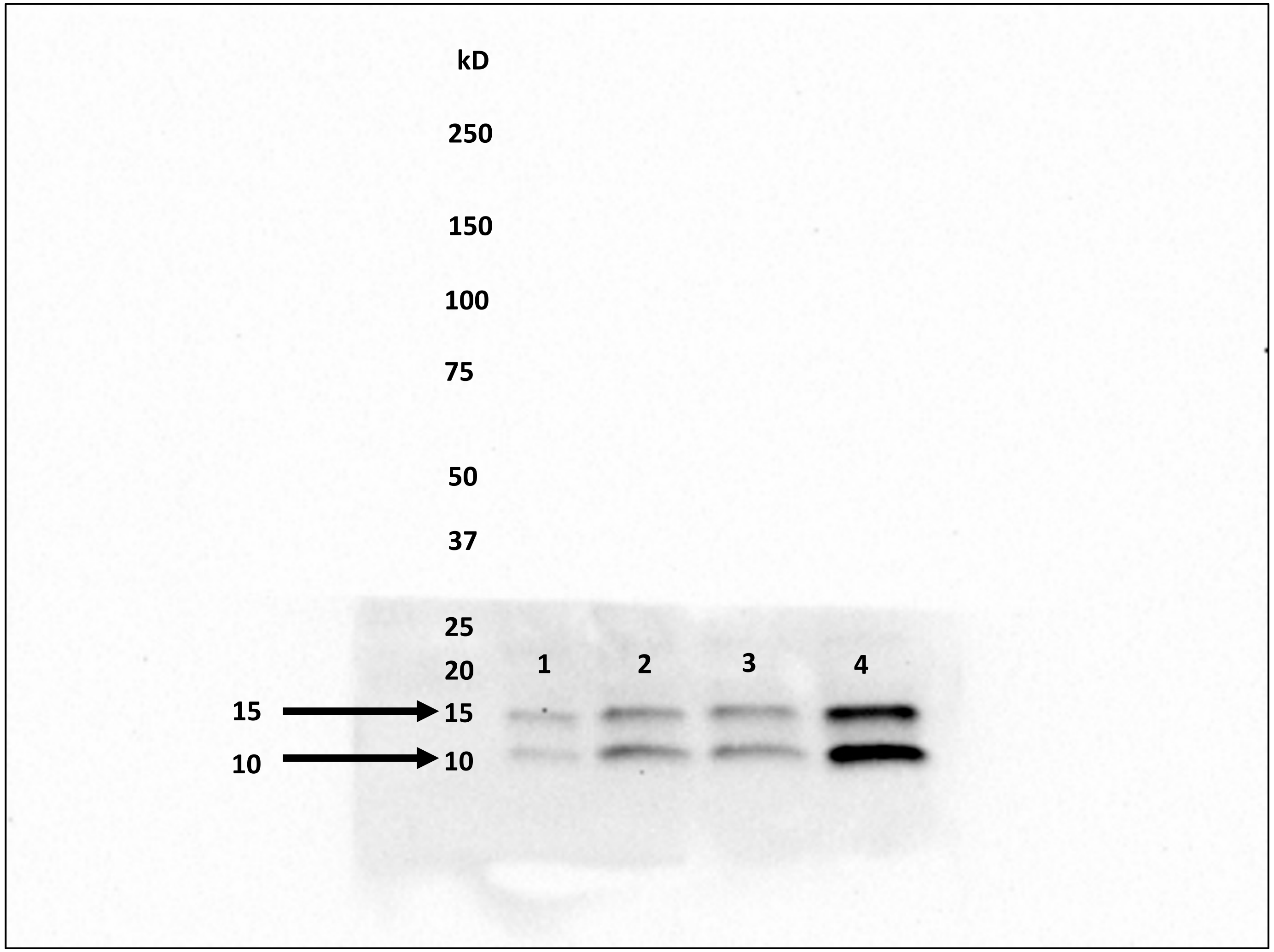

Figure 4B
Probing: α-Tubulin (T9026)
Millipore Sigma

Loading order:
Lane 1: Control at 72 h
Lane 2: 24h 7mM DHA
Lane 3: 48h 7mM DHA
Lane 4: 72 h 7mM DHA

Imaged with Bio-Rad ChemiDoc XRS
Imaging system
Chemi Hi Sensitivity setting

Note: Non-specific bands from previous antibody probing

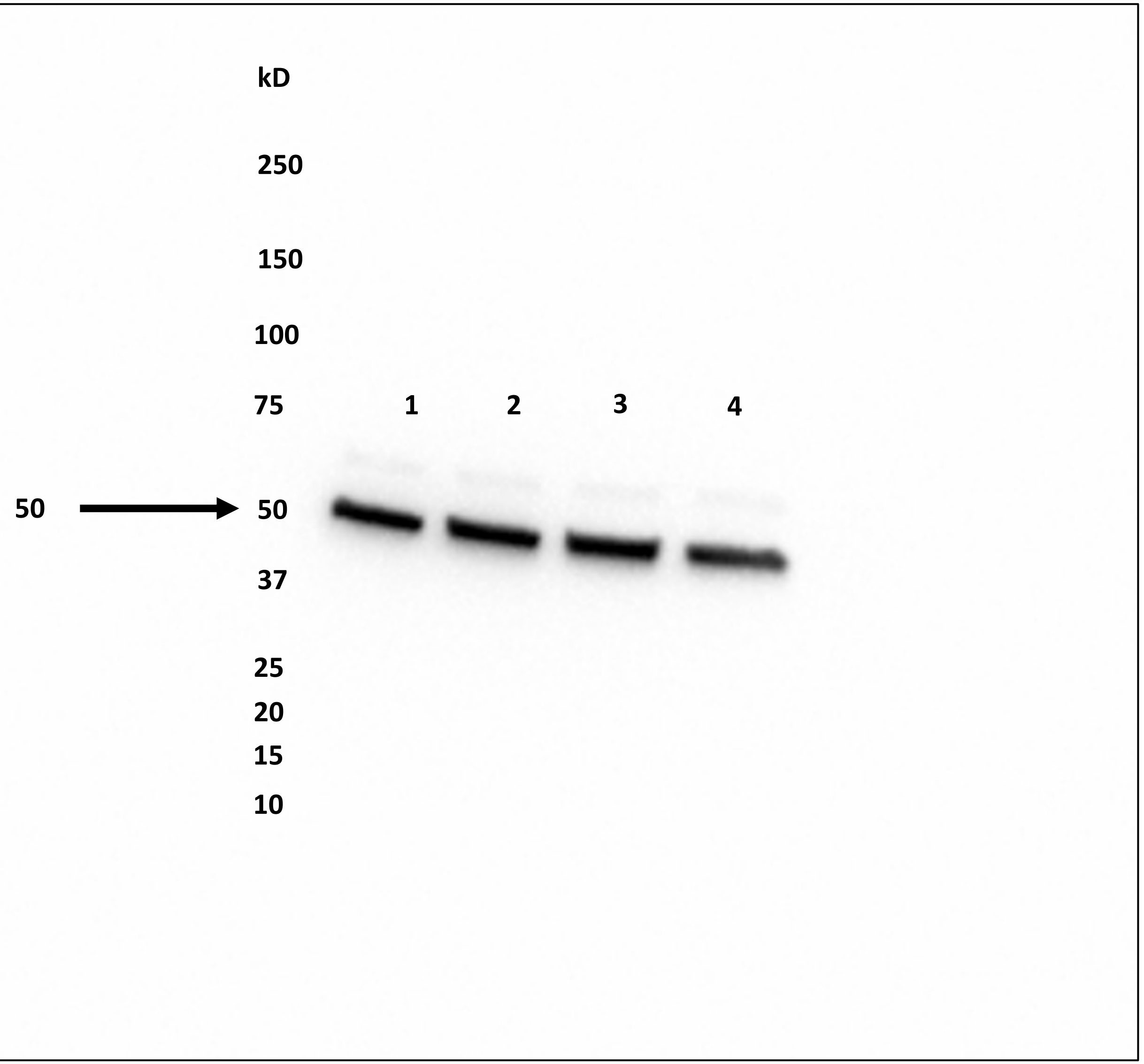

Figure 4B
Probing: LAMP-1 (D2D11) (9091)
Cell Signaling Technologies

Loading order:
Lane 1: Control at 72 h
Lane 2: 24h 7mM DHA
Lane 3: 48h 7mM DHA
Lane 4: 72 h 7mM DHA

Imaged with Bio-Rad ChemiDoc XRS
Imaging system
Chemi Hi Sensitivity setting

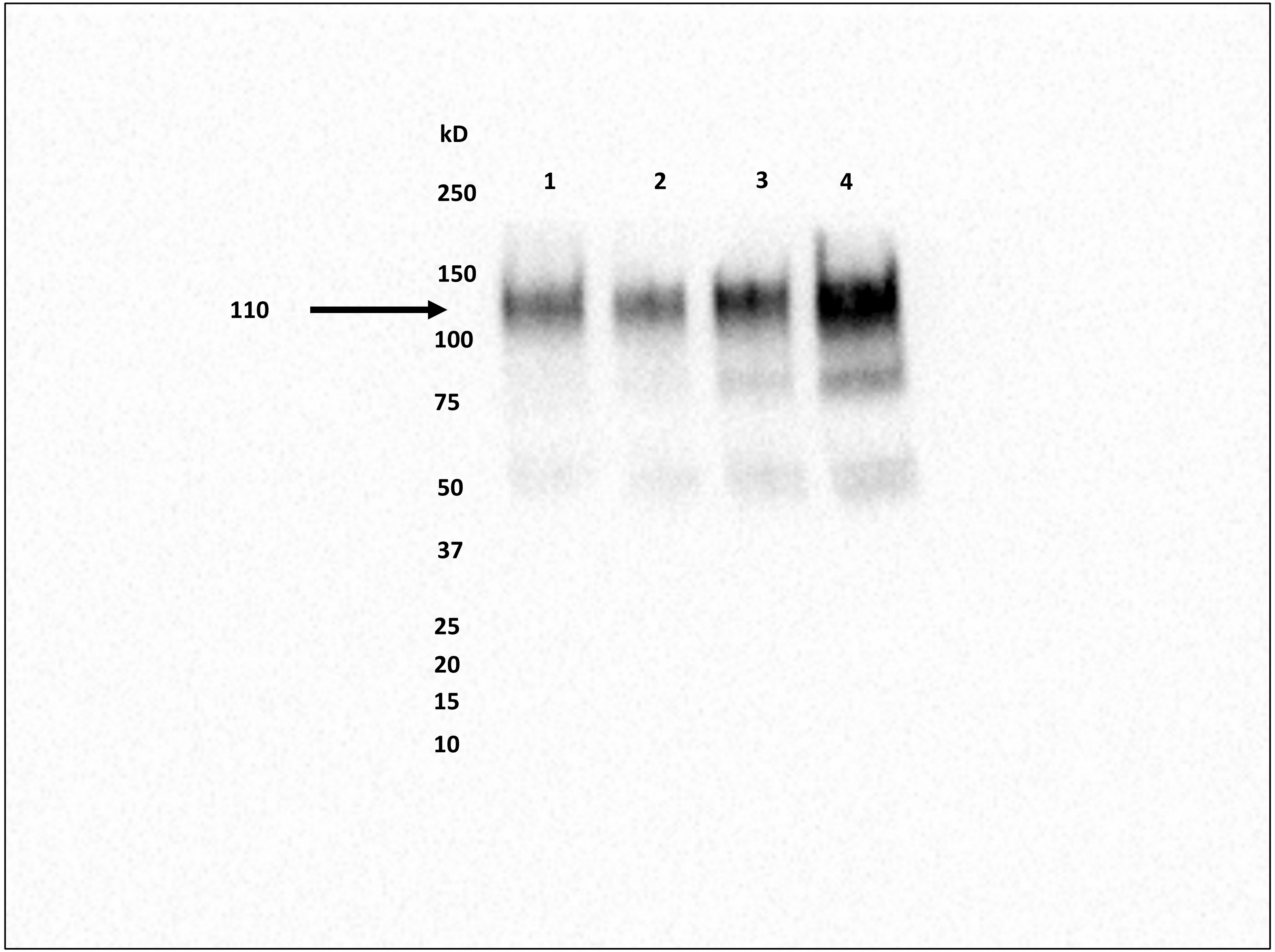

Figure 4B
Probing: α-Tubulin (T9026)
Millipore Sigma

Loading order:
Lane 1: Control at 72 h
Lane 2: 24h 7mM DHA
Lane 3: 48h 7mM DHA
Lane 4: 72 h 7mM DHA

Imaged with Bio-Rad ChemiDoc XRS
Imaging system
Chemi Hi Sensitivity setting

Note: Non-specific bands from previous
antibody probing

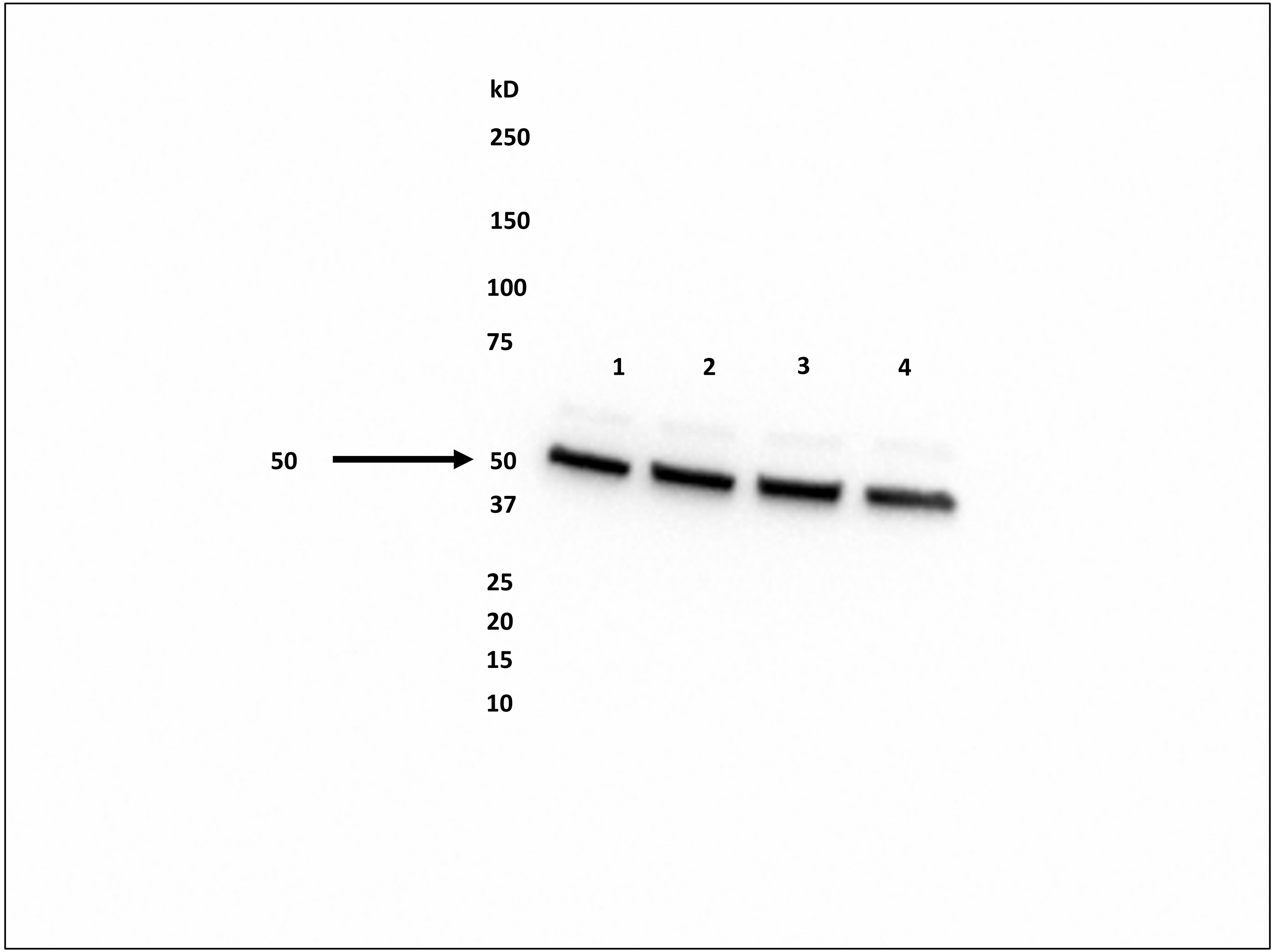

Figure 4B
Probing: Cathepsin B (D1C7Y) (31718)
Cell Signaling Technologies

Loading order:
Lane 1: Control at 96 h
Lane 2: 24h 7mM DHA
Lane 3: 48h 7mM DHA
Lane 4: 72 h 7mM DHA
Lane 5: 96 h 7 mM DHA

Imaged with Bio-Rad ChemiDoc XRS
Imaging system
Chemi Hi Sensitivity setting

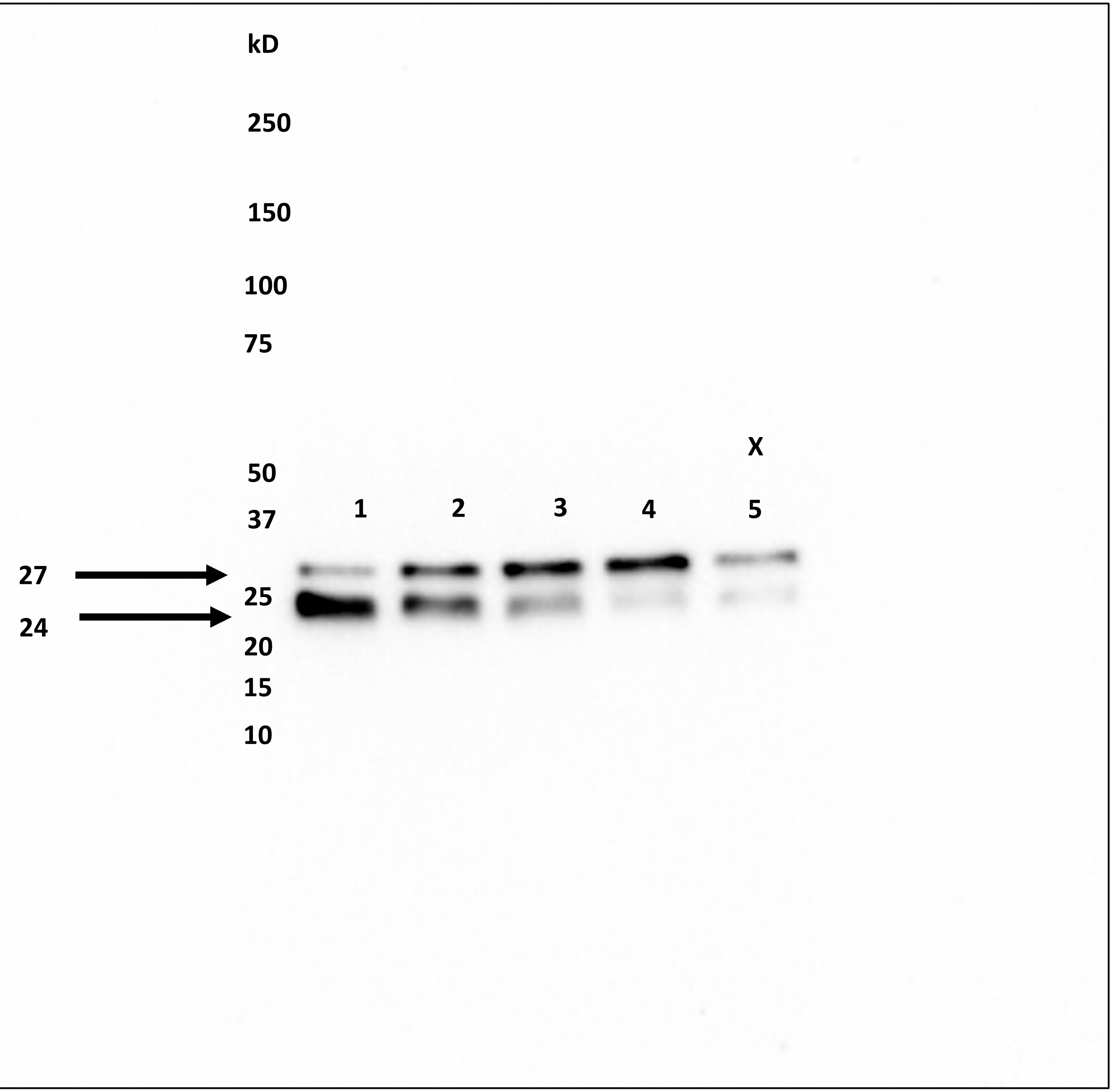

Figure 4B
Probing: α-Tubulin (T9026)
Millipore Sigma

Loading order:
Lane 1: Control at 96 h
Lane 2: 24h 7mM DHA
Lane 3: 48h 7mM DHA
Lane 4: 72 h 7mM DHA
Lane 5: 96 h 7 mM DHA

Imaged with Bio-Rad ChemiDoc XRS Imaging system
Chemi Hi Sensitivity setting

Note: Non-specific bands from previous antibody probing

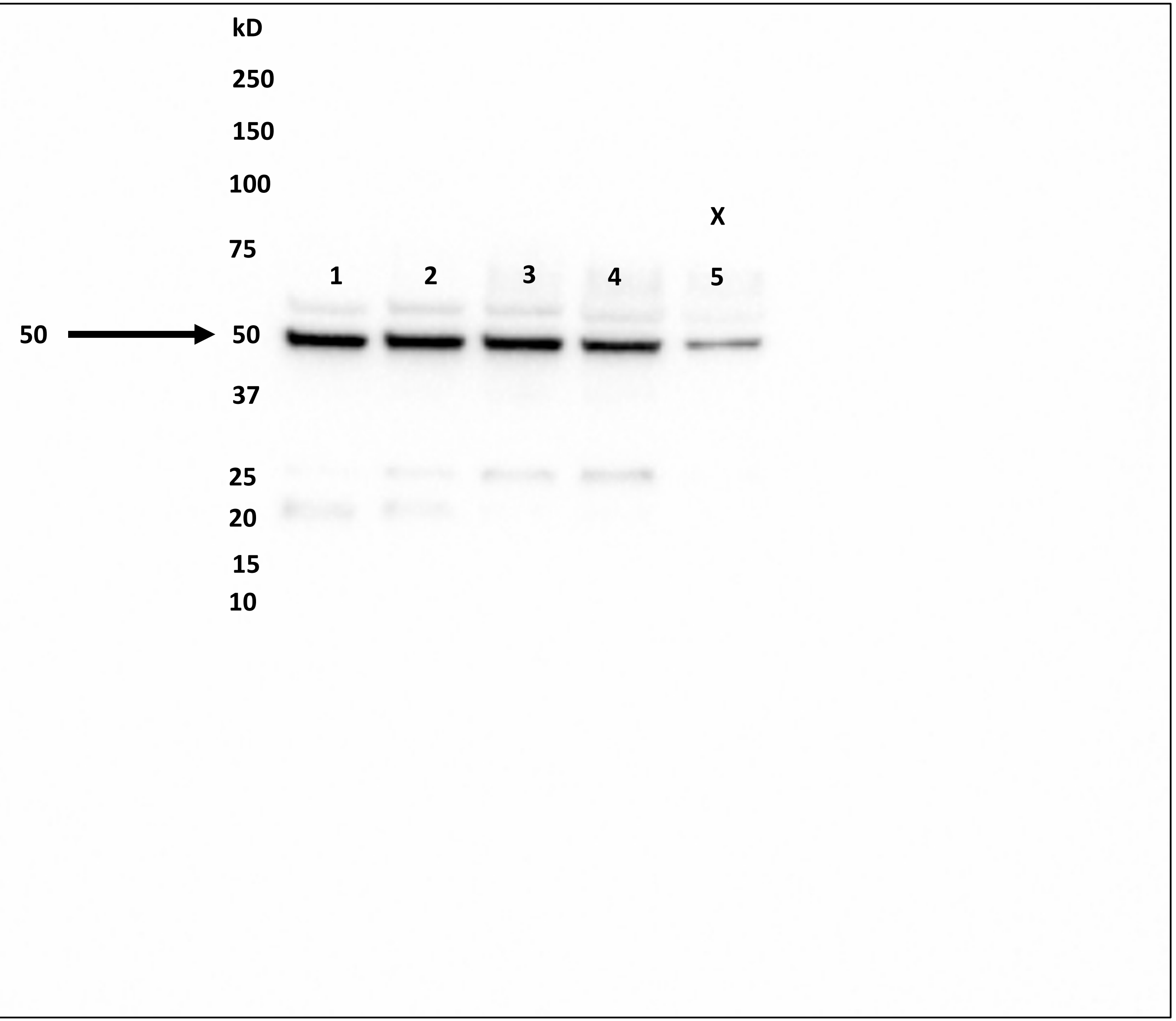

Figure 5A
Probing: p-mTOR (Ser2448) (2972)
Cell Signaling Technologies

Loading order:
Lane 1: Control at 96 h
Lane 2: 24h 7mM DHA
Lane 3: 48h 7mM DHA
Lane 4: 72 h 7mM DHA
Lane 5: 96 h 7 mM DHA

Imaged with Bio-Rad ChemiDoc XRS
Imaging system
Chemi Hi Sensitivity setting

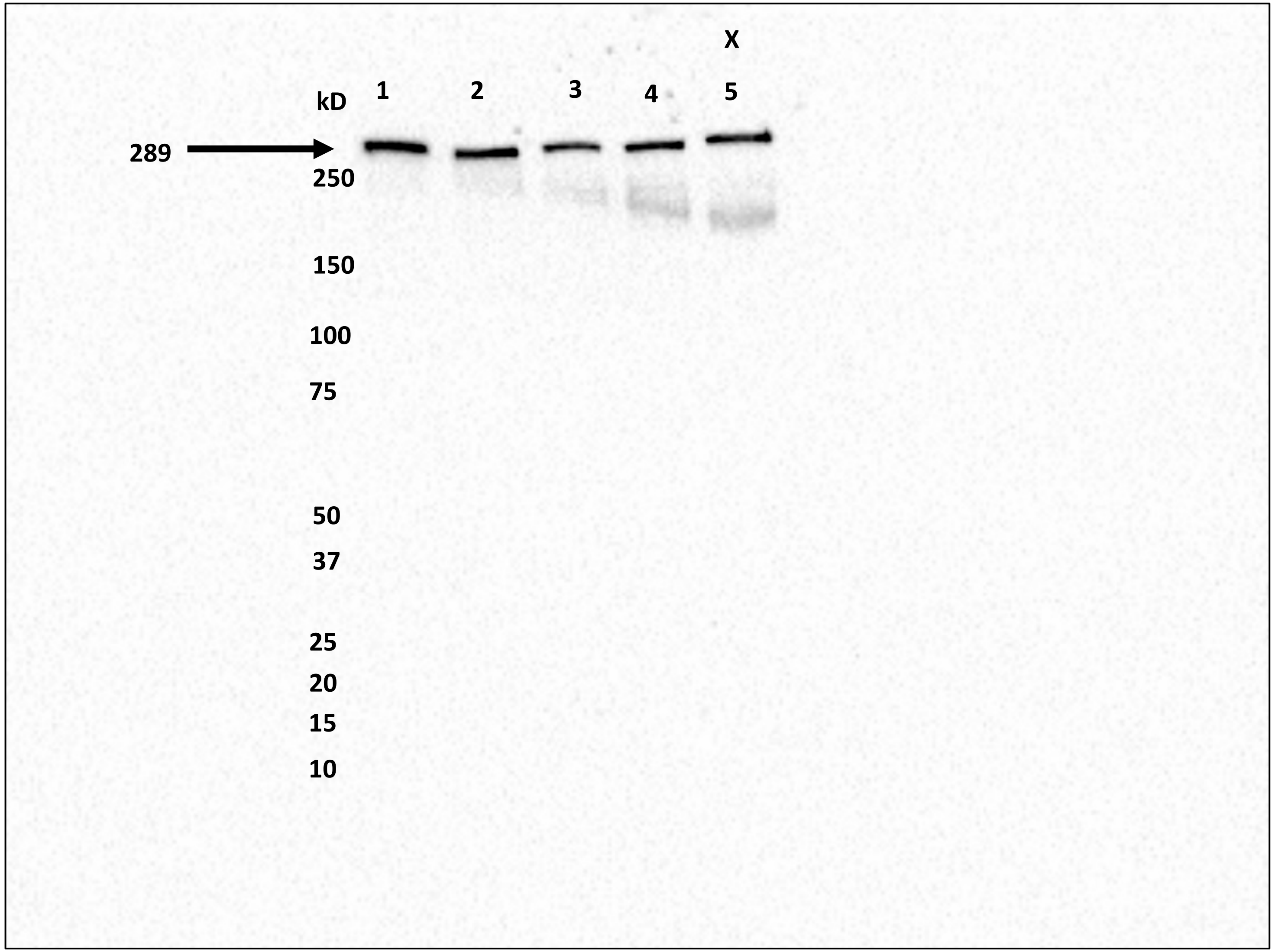

Figure 5A
Probing: mTOR (2972)
Cell Signaling Technologies

Loading order:
Lane 1: Control at 96 h
Lane 2: 24h 7mM DHA
Lane 3: 48h 7mM DHA
Lane 4: 72 h 7mM DHA
Lane 5: 96 h 7 mM DHA

Imaged with Bio-Rad ChemiDoc XRS
Imaging system
Chemi Hi Sensitivity setting

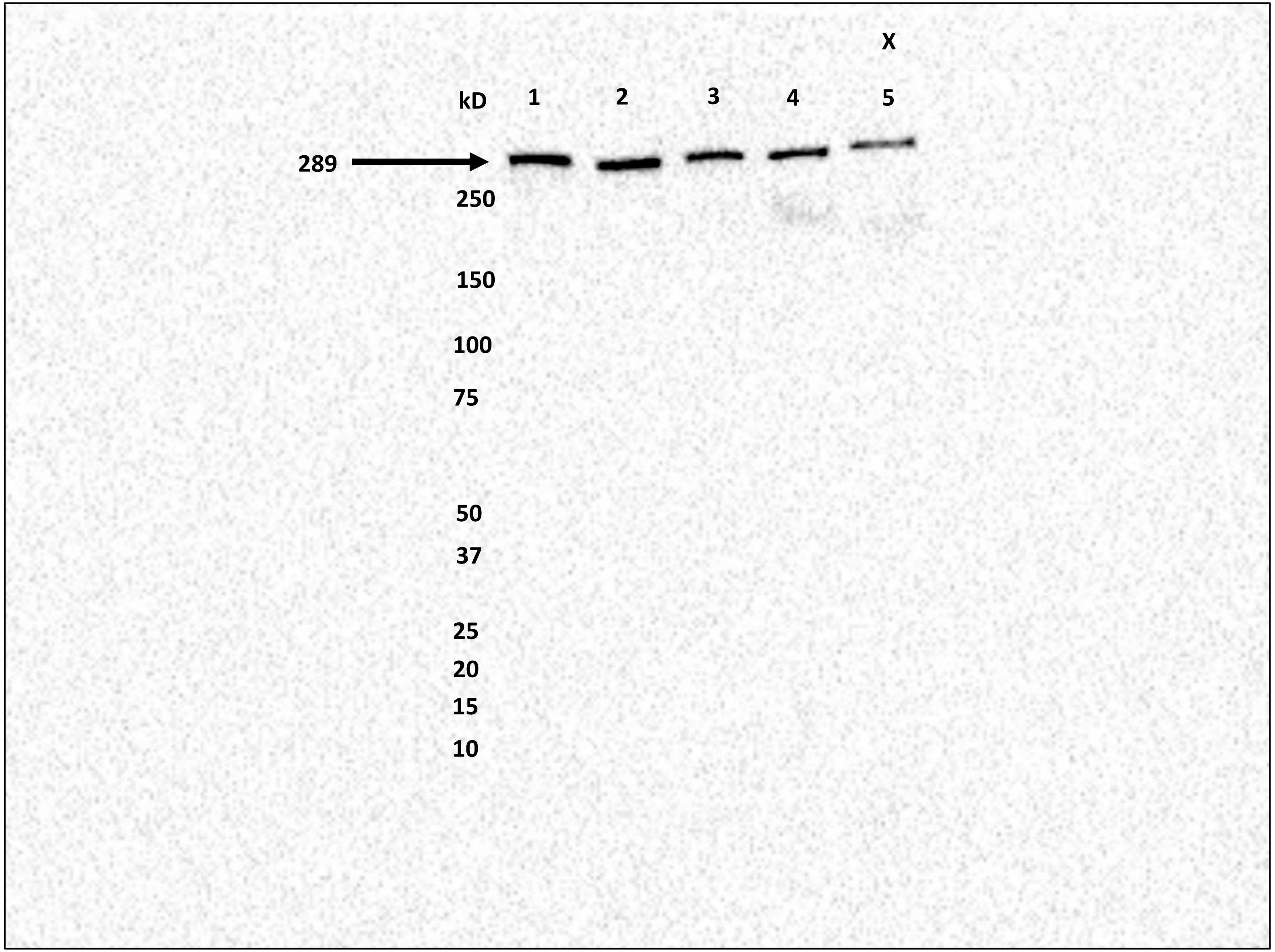

Figure 5A
Probing: α-Tubulin (T9026)
Millipore Sigma

Loading order:
Lane 1: Control at 96 h
Lane 2: 24h 7mM DHA
Lane 3: 48h 7mM DHA
Lane 4: 72 h 7mM DHA
Lane 5: 96 h 7 mM DHA

Imaged with Bio-Rad ChemiDoc XRS
Imaging system
Chemi Hi Sensitivity setting

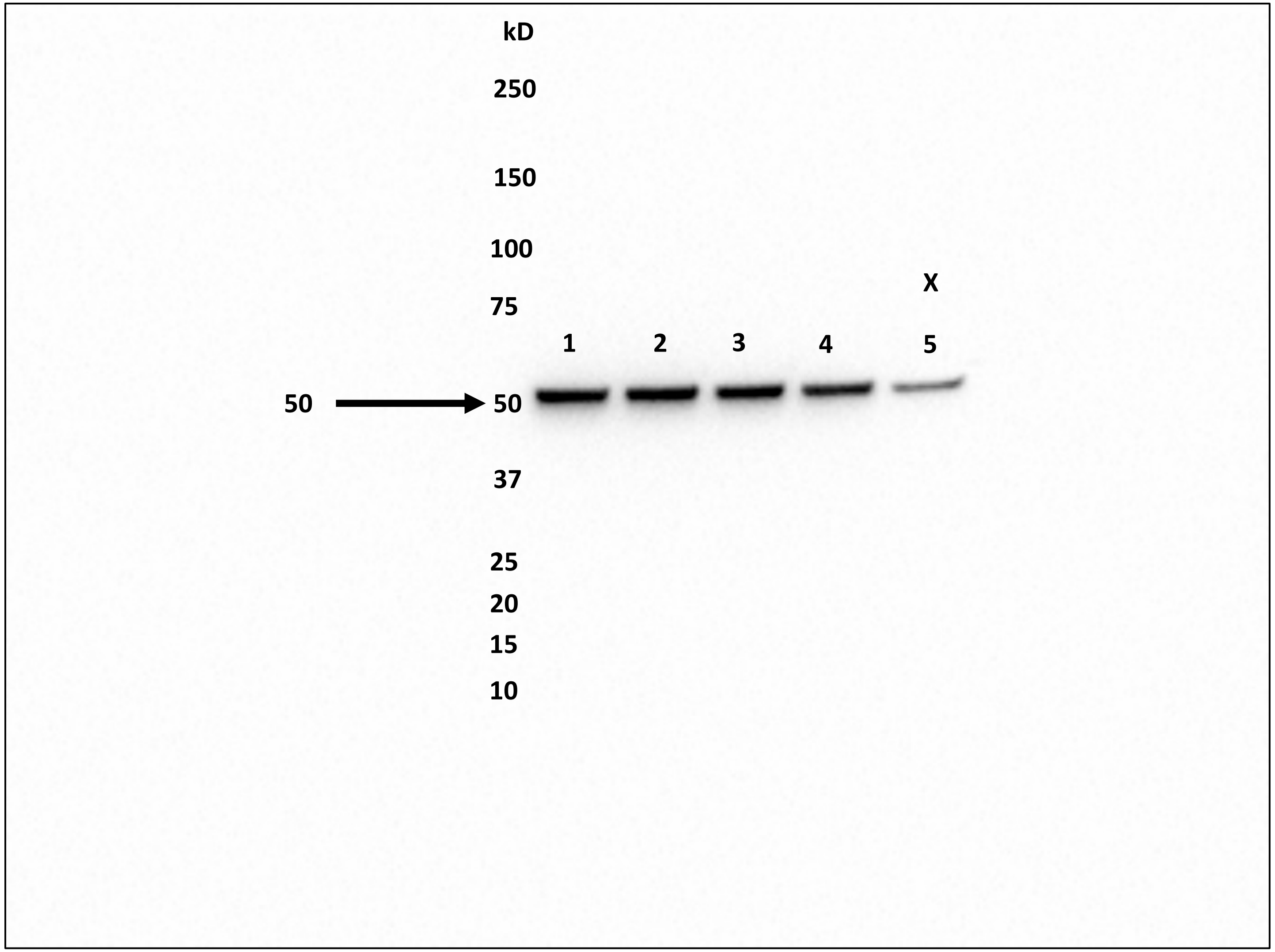

Figure 5B
Probing: p-mTOR (Ser2448) (2972)
Cell Signaling Technologies

Loading order:
Lane 1: Control at 24 h
Lane 2: 1h 7mM DHA
Lane 3: 4h 7mM DHA
Lane 4: 24 h 7mM DHA

Imaged with Bio-Rad ChemiDoc XRS
Imaging system
Chemi Hi Sensitivity setting

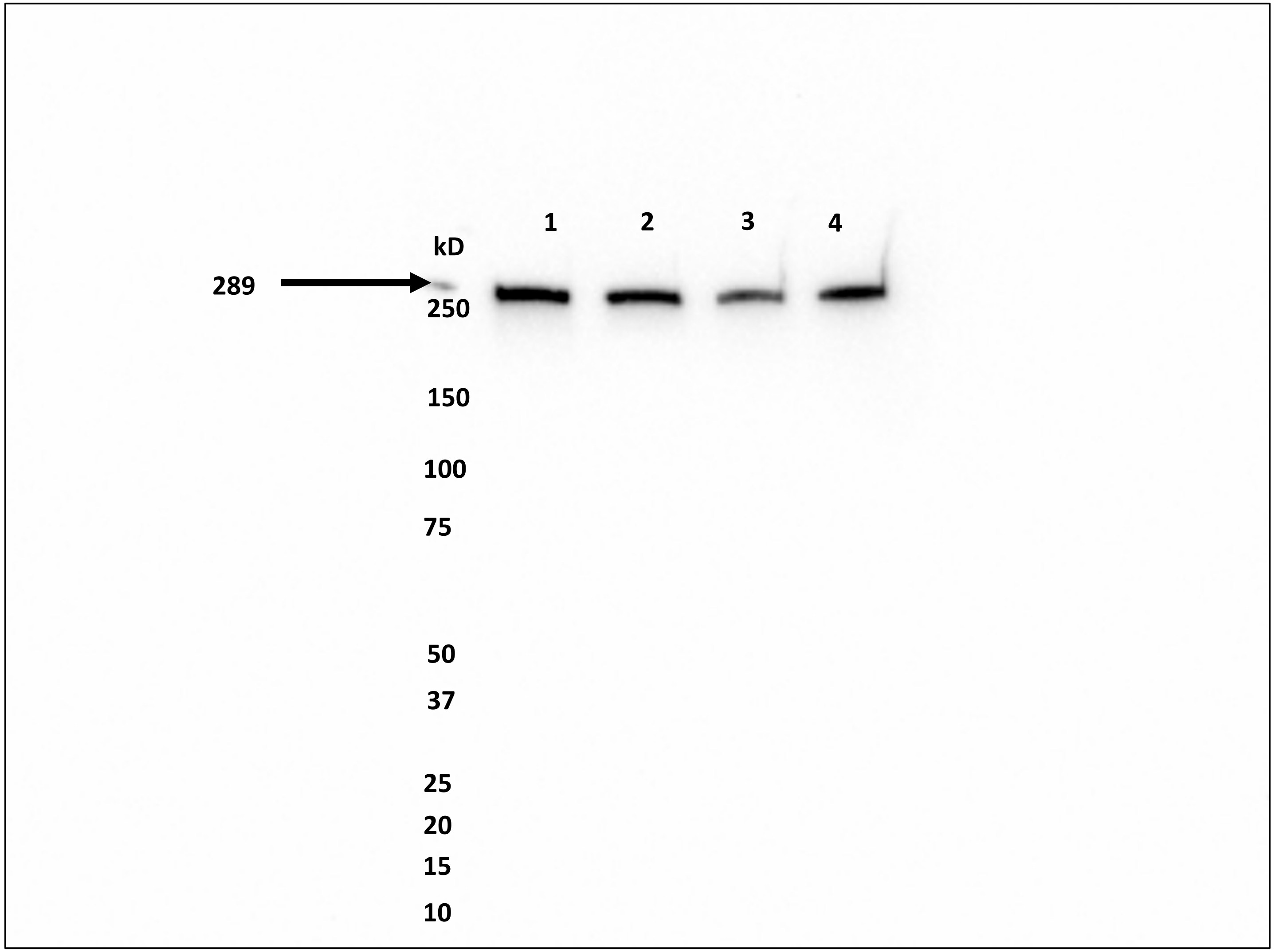

Figure 5B
Probing: mTOR (2972)
Cell Signaling Technologies

Loading order:
Lane 1: Control at 24 h
Lane 2: 1h 7mM DHA
Lane 3: 4h 7mM DHA
Lane 4: 24 h 7mM DHA

Imaged with Bio-Rad ChemiDoc XRS
Imaging system
Chemi Hi Sensitivity setting

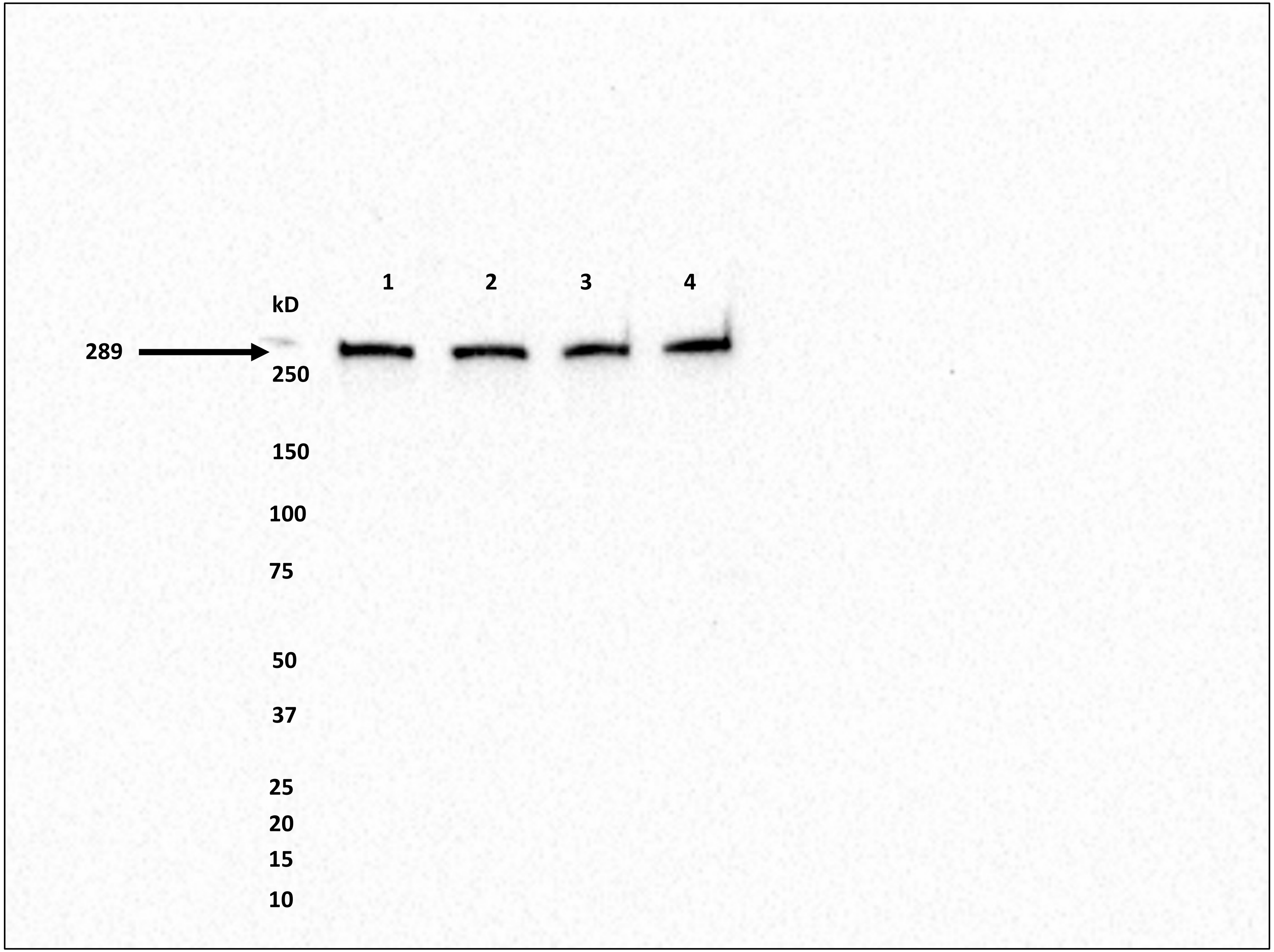

Figure 5B
Probing: α-Tubulin (T9026)
Millipore Sigma

Loading order:
Lane 1: Control at 24 h
Lane 2: 1h 7mM DHA
Lane 3: 4h 7mM DHA
Lane 4: 24 h 7mM DHA

Imaged with Bio-Rad ChemiDoc XRS
Imaging system
Chemi Hi Sensitivity setting

Note: Non-specific bands from previous
antibody probing

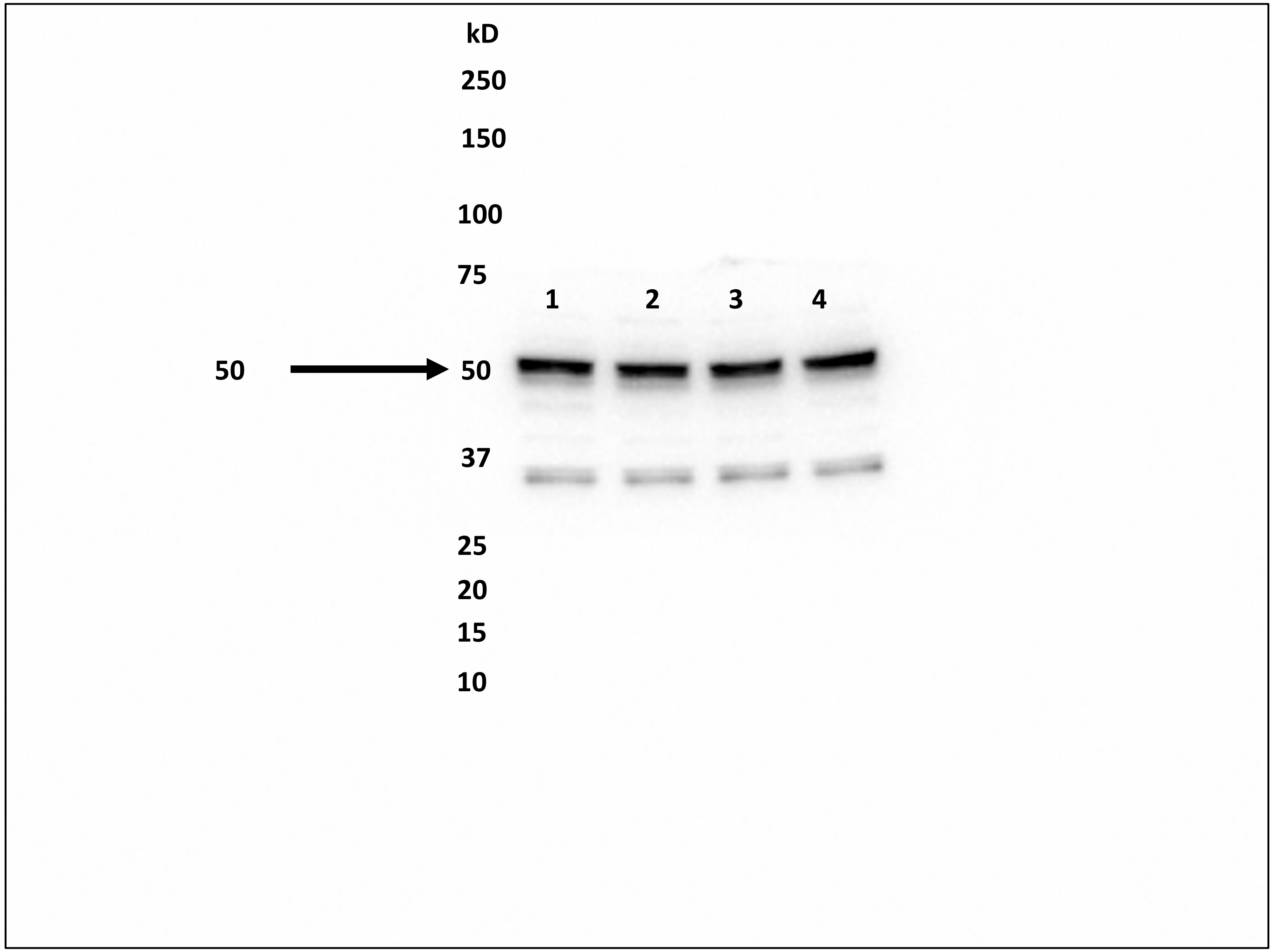

Figure 5C
Probing: RAPTOR (24C12) (2280)
Cell Signaling Technologies

Loading order:
Lane 1: Control at 96 h
Lane 2: 24h 7mM DHA
Lane 3: 48h 7mM DHA
Lane 4: 72 h 7mM DHA
Lane 5: 96 h 7 mM DHA

Imaged with Bio-Rad ChemiDoc XRS
Imaging system
Chemi Hi Sensitivity setting

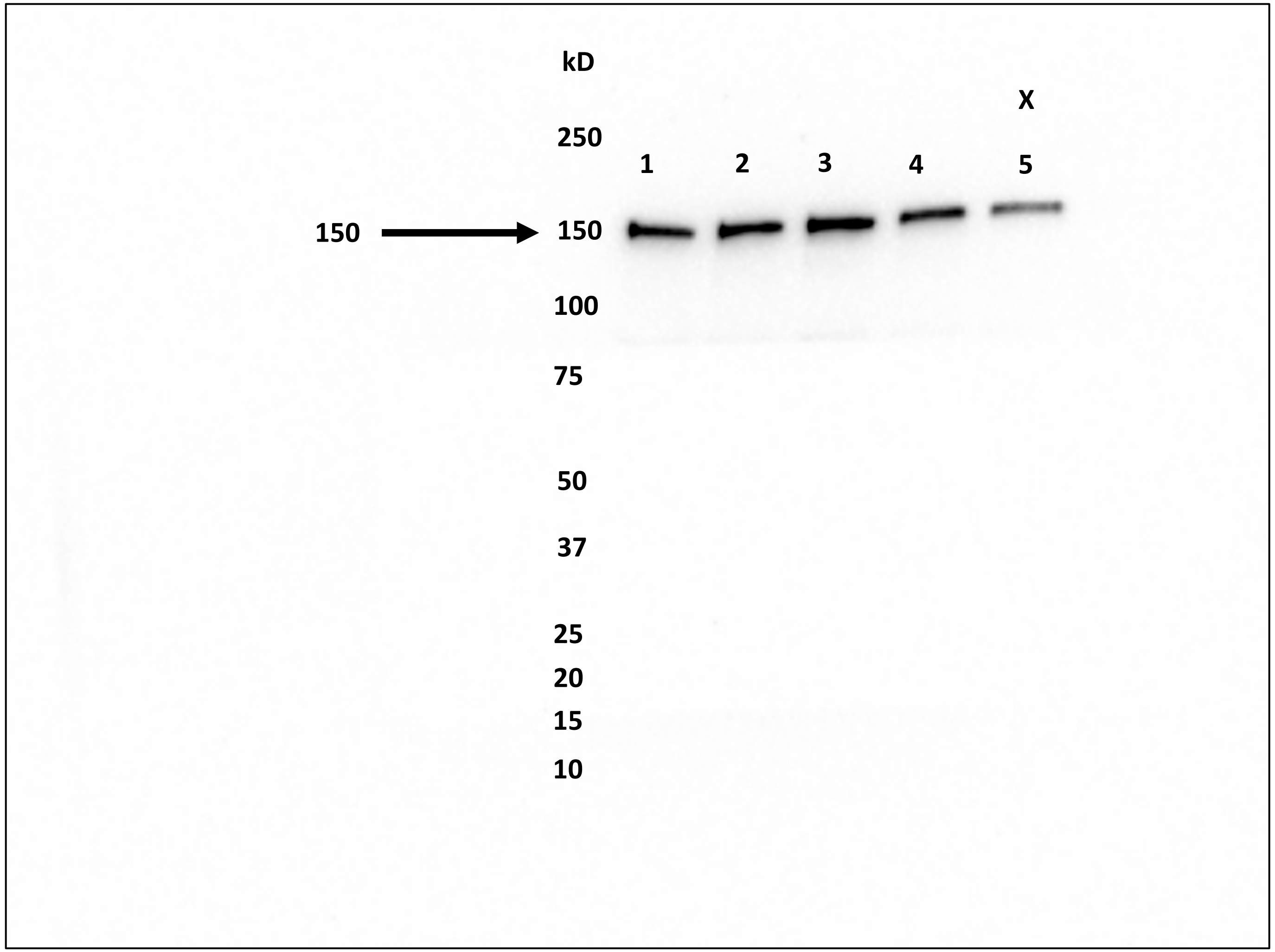

Figure 5C
Probing: α-Tubulin (T9026)
Millipore Sigma

Loading order:
Lane 1: Control at 96 h
Lane 2: 24h 7mM DHA
Lane 3: 48h 7mM DHA
Lane 4: 72 h 7mM DHA
Lane 5: 96 h 7 mM DHA

Imaged with Bio-Rad ChemiDoc XRS
Imaging system
Chemi Hi Sensitivity setting

Note: Non-specific bands from previous
antibody probing

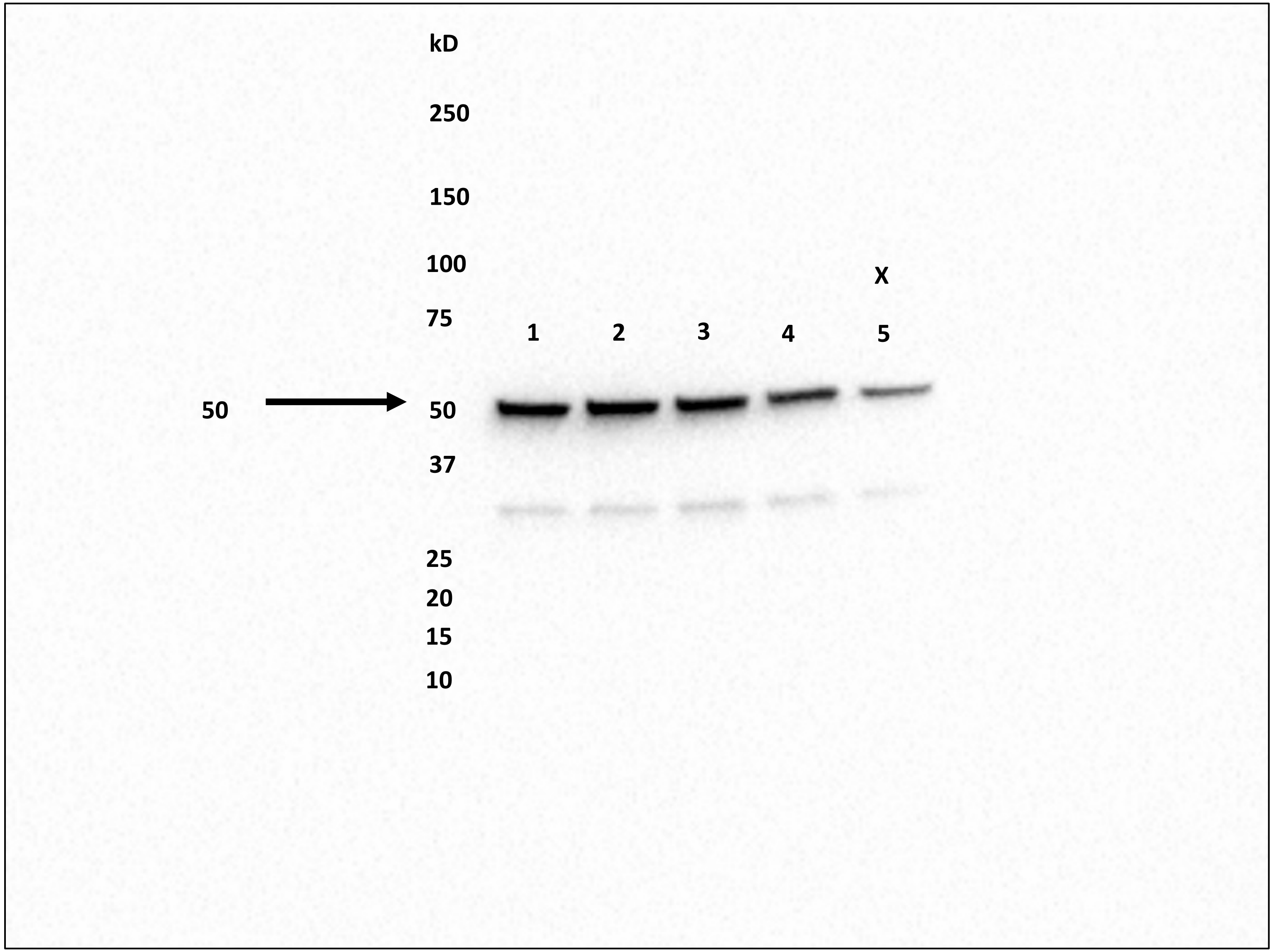

Figure 5C
Probing: RICTOR (53A2) (2114)
Cell Signaling Technologies

Loading order:
Lane 1: Control at 72 h
Lane 2: 24h 7mM DHA
Lane 3: 48h 7mM DHA
Lane 4: 72 h 7mM DHA

Imaged with Bio-Rad ChemiDoc XRS
Imaging system
Chemi Hi Sensitivity setting

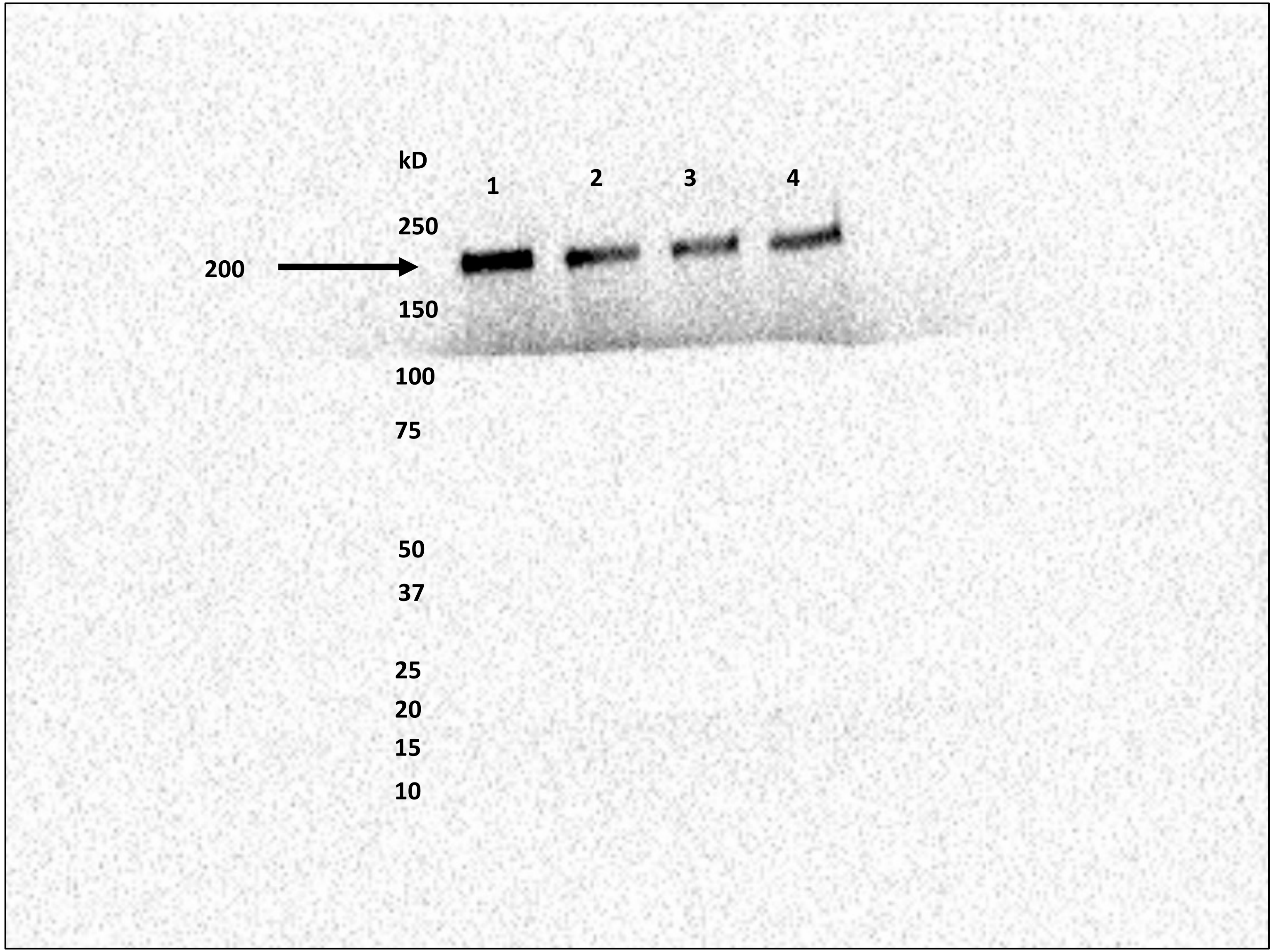

Figure 5C
Probing: α-Tubulin (T9026)
Millipore Sigma

Loading order:
Lane 1: Control at 72 h
Lane 2: 24h 7mM DHA
Lane 3: 48h 7mM DHA
Lane 4: 72 h 7mM DHA

Imaged with Bio-Rad ChemiDoc XRS
Imaging system
Chemi Hi Sensitivity setting

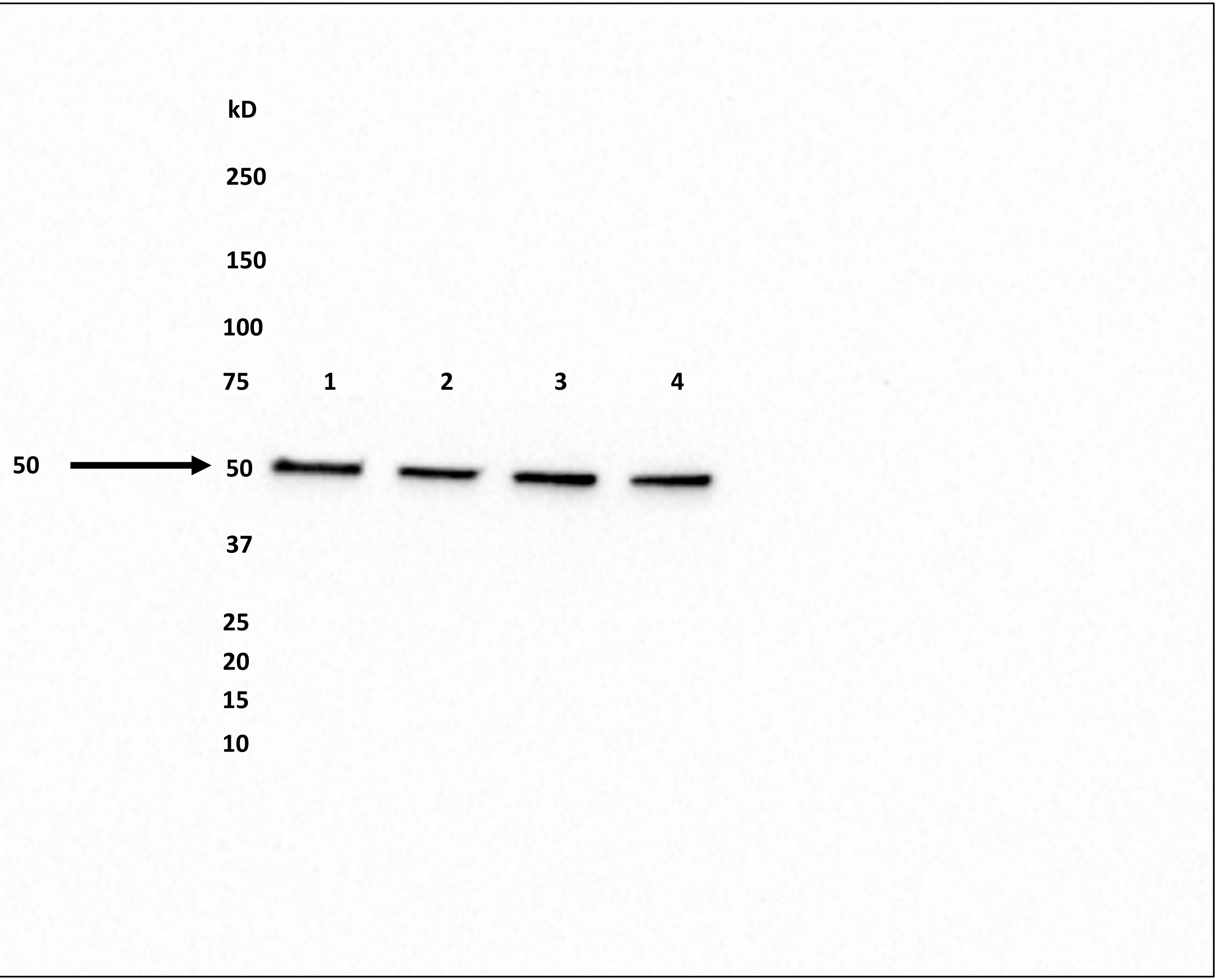

Figure 5D
Probing: RAPTOR (24C12) (2280)
Cell Signaling Technologies

Loading order:
Lane 1: Control at 24 h
Lane 2: 1h 7mM DHA
Lane 3: 4h 7mM DHA
Lane 4: 24 h 7mM DHA

Imaged with Bio-Rad ChemiDoc XRS
Imaging system
Chemi Hi Sensitivity setting

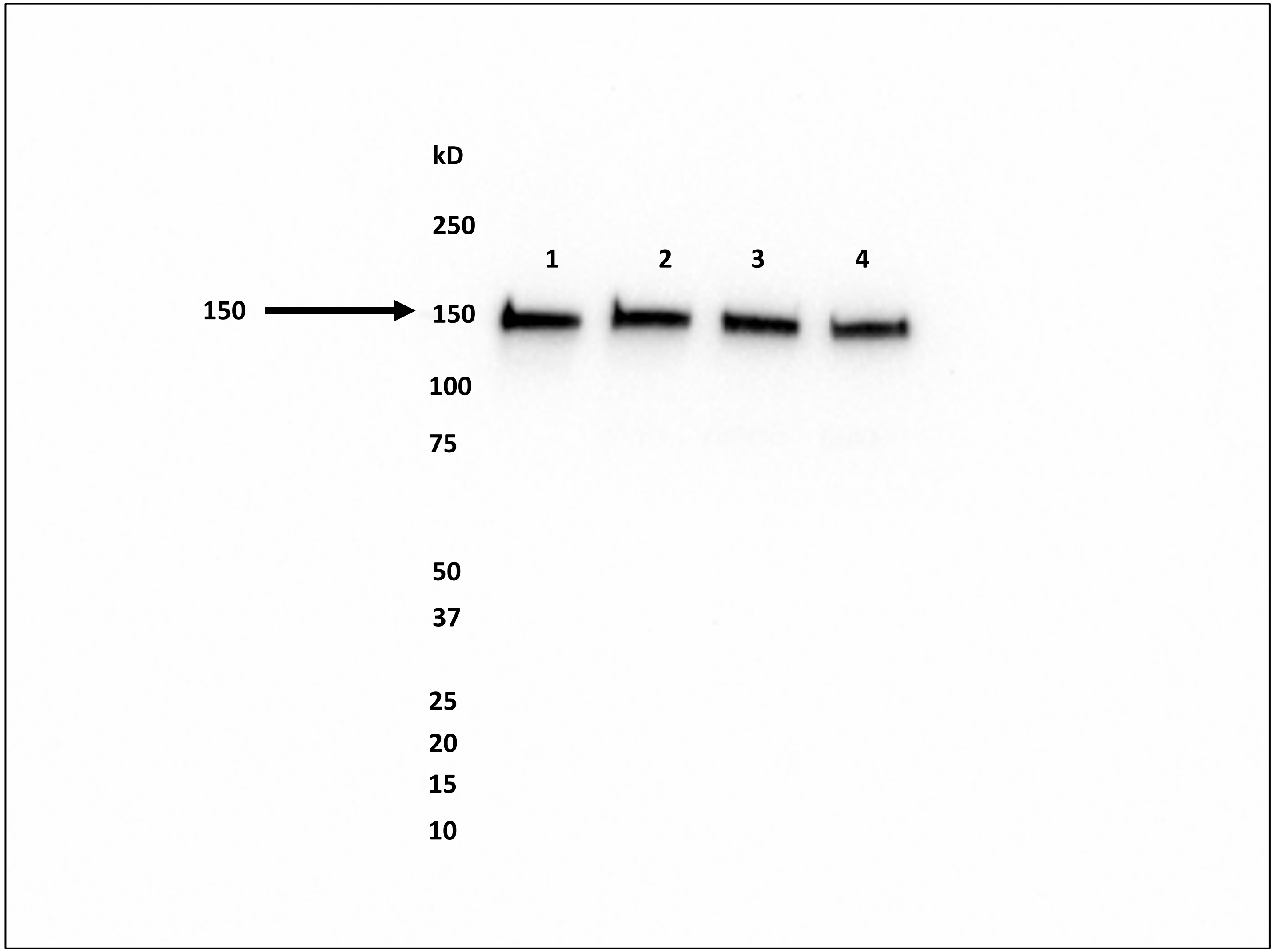

Figure 5D
Probing: α-Tubulin (T9026)
Millipore Sigma

Loading order:
Lane 1: Control at 24 h
Lane 2: 1h 7mM DHA
Lane 3: 4h 7mM DHA
Lane 4: 24 h 7mM DHA

Imaged with Bio-Rad ChemiDoc XRS
Imaging system
Chemi Hi Sensitivity setting

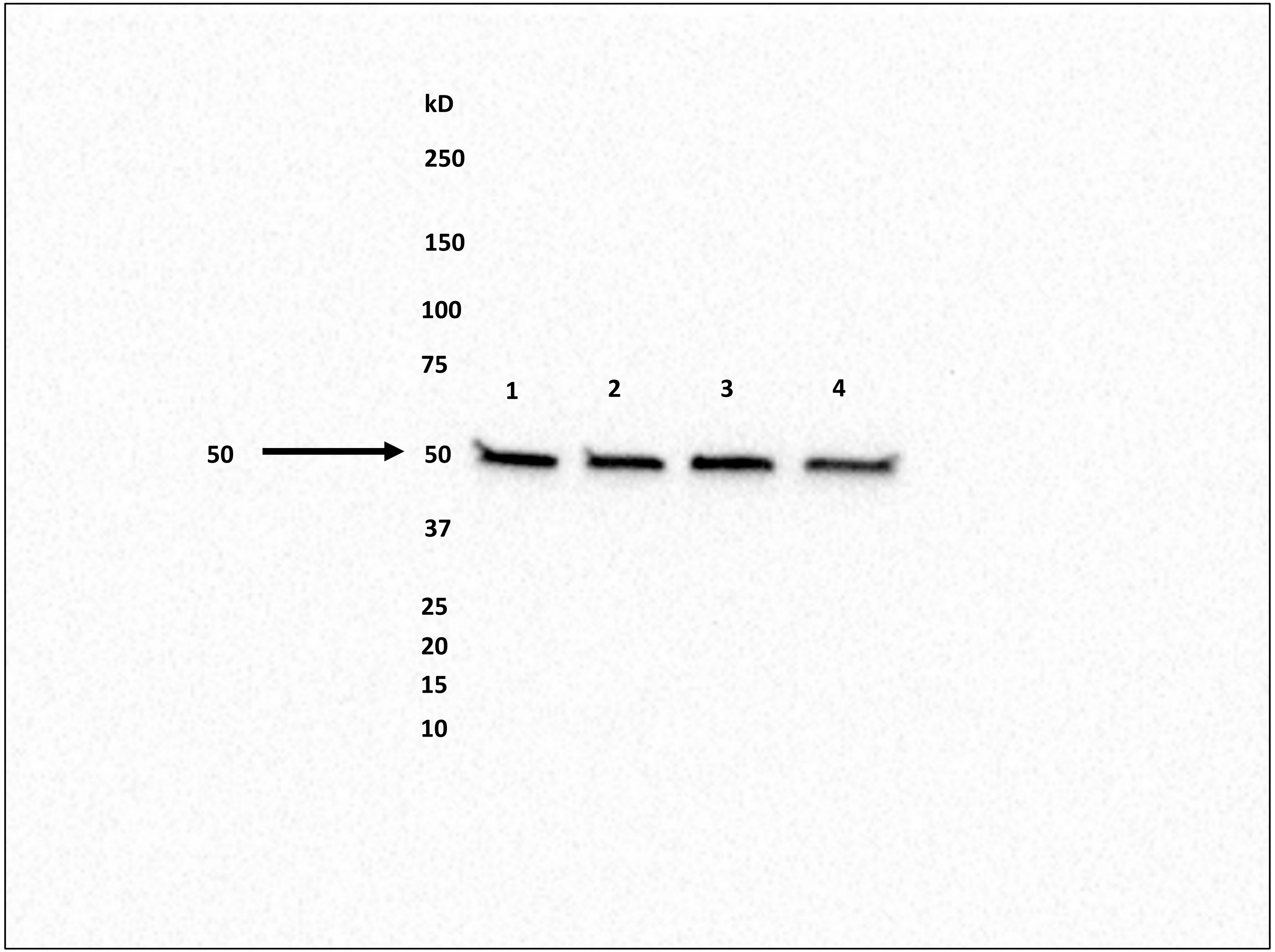

Figure 5D
Probing: RICTOR (53A2) (2114)
Cell Signaling Technologies

Loading order:
Lane 1: Control at 24 h
Lane 2: 1h 7mM DHA
Lane 3: 4h 7mM DHA
Lane 4: 24 h 7mM DHA

Imaged with Bio-Rad ChemiDoc XRS
Imaging system
Chemi Hi Sensitivity setting

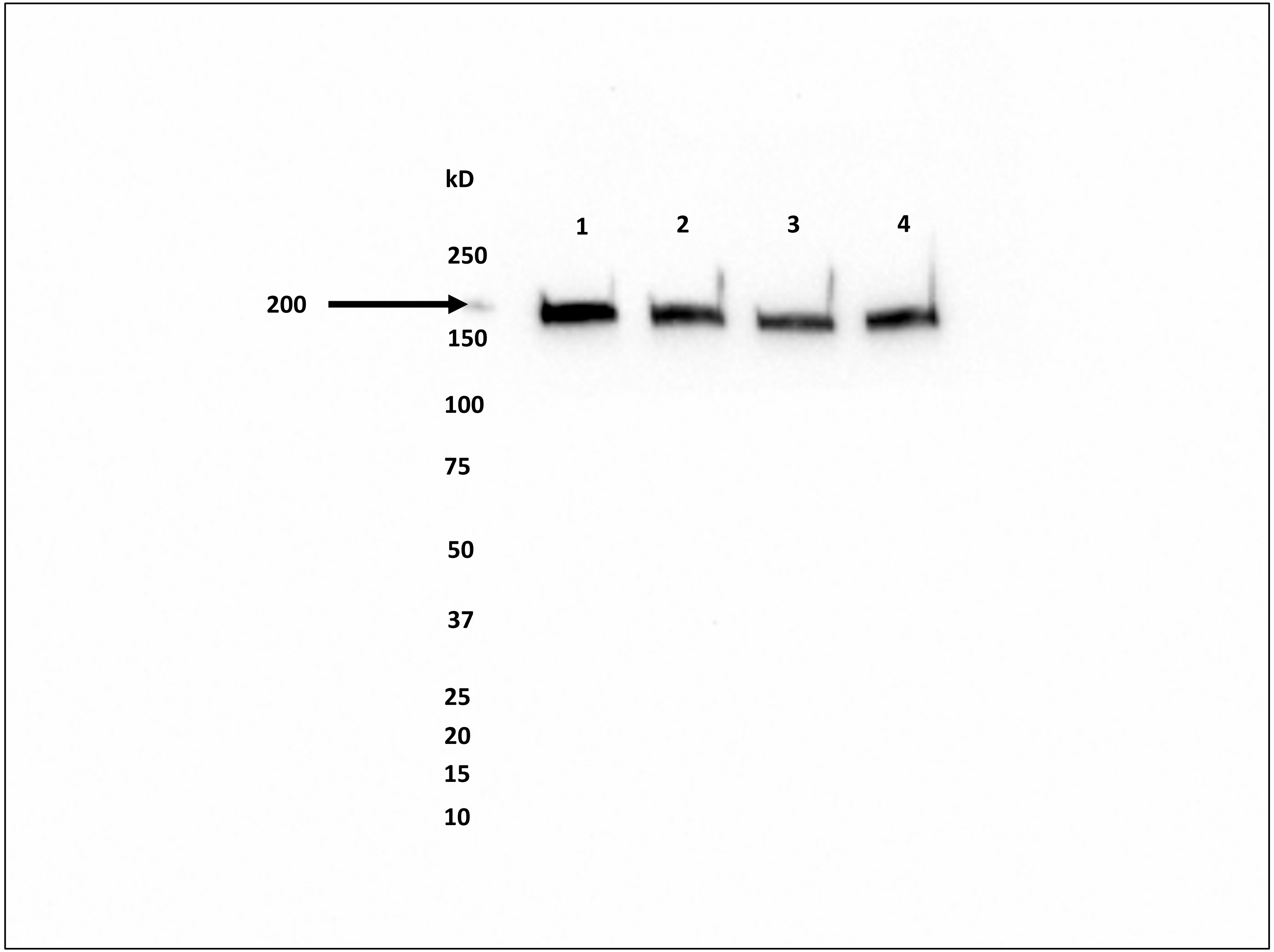

Figure 5D
Probing: α-Tubulin (T9026)
Millipore Sigma

Loading order:
Lane 1: Control at 24 h
Lane 2: 1h 7mM DHA
Lane 3: 4h 7mM DHA
Lane 4: 24 h 7mM DHA

Imaged with Bio-Rad ChemiDoc XRS
Imaging system
Chemi Hi Sensitivity setting

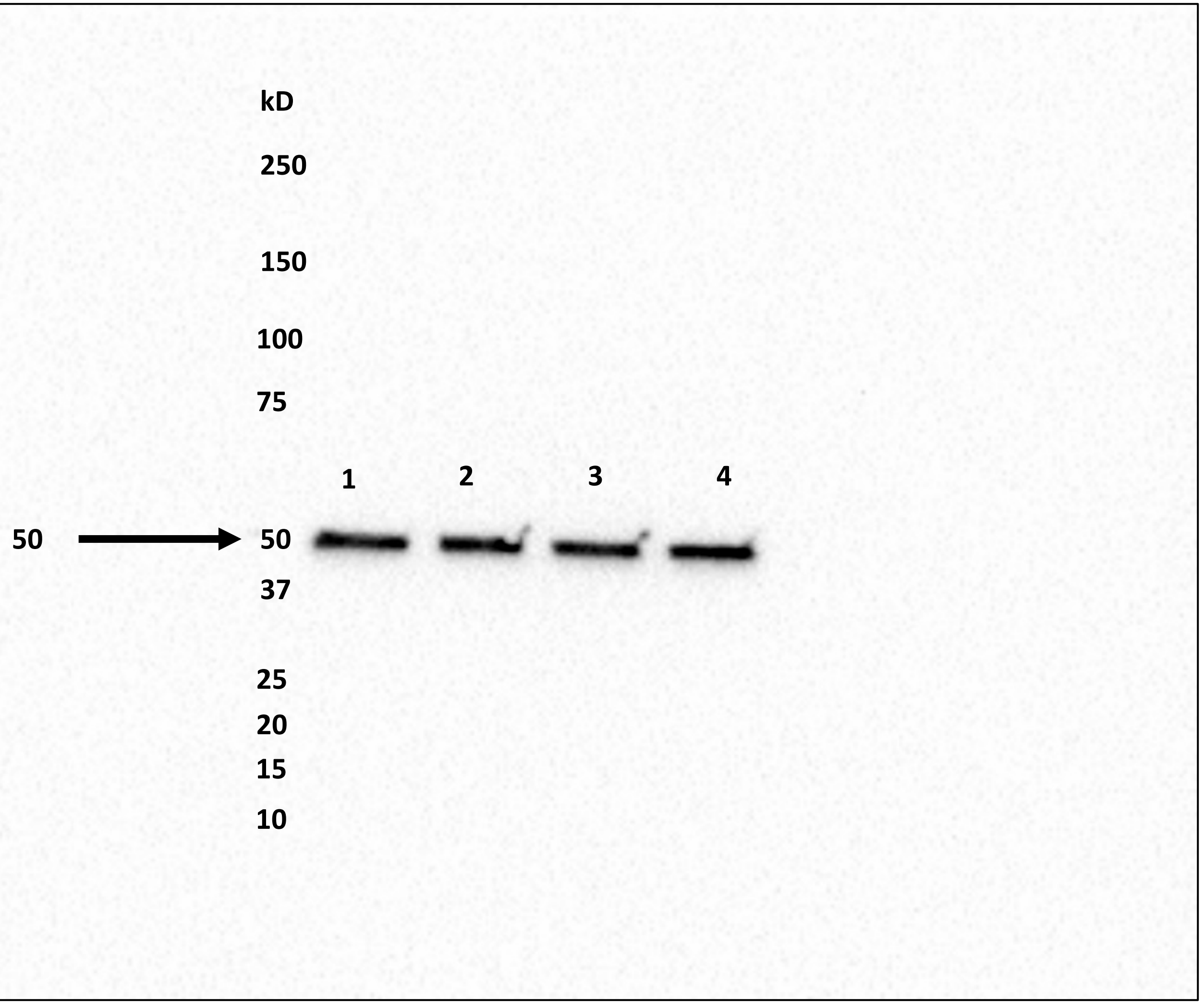

Figure 5E
Probing: p-AKT (Ser473)(D9E) (4060)
Cell Signaling Technologies

Loading order:
Lane 1: Control at 72 h
Lane 2: 24h 7mM DHA
Lane 3: 48h 7mM DHA
Lane 4: 72 h 7mM DHA

Imaged with Bio-Rad ChemiDoc XRS
Imaging system
Chemi Hi Sensitivity setting

Note: Non-specific bands from previous
antibody probing

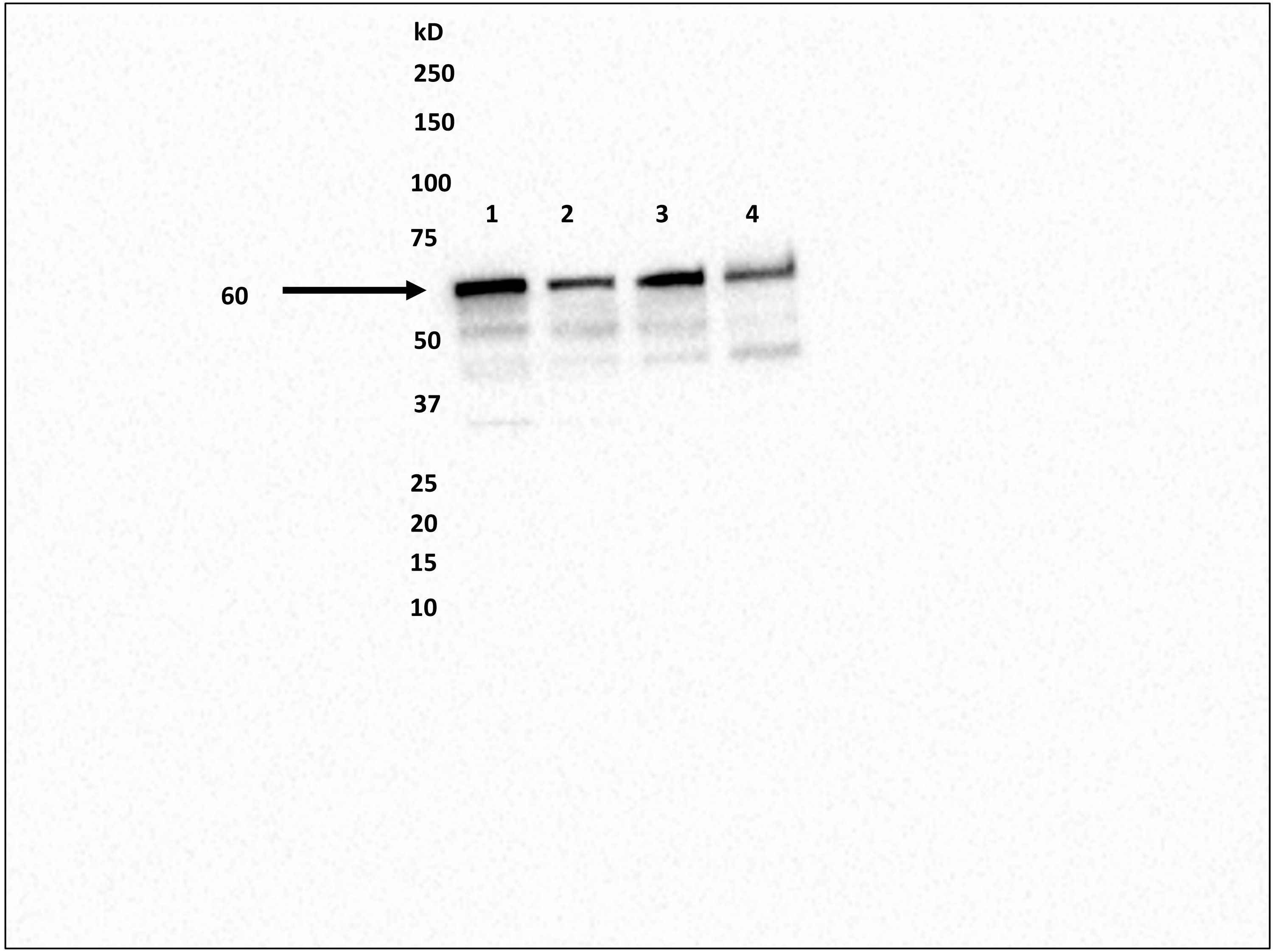

Figure 5E
Probing: AKT (C67E7) (4691)
Cell Signaling Technolgies

Loading order:
Lane 1: Control at 72 h
Lane 2: 24h 7mM DHA
Lane 3: 48h 7mM DHA
Lane 4: 72 h 7mM DHA

Imaged with Bio-Rad ChemiDoc XRS
Imaging system
Chemi Hi Sensitivity setting

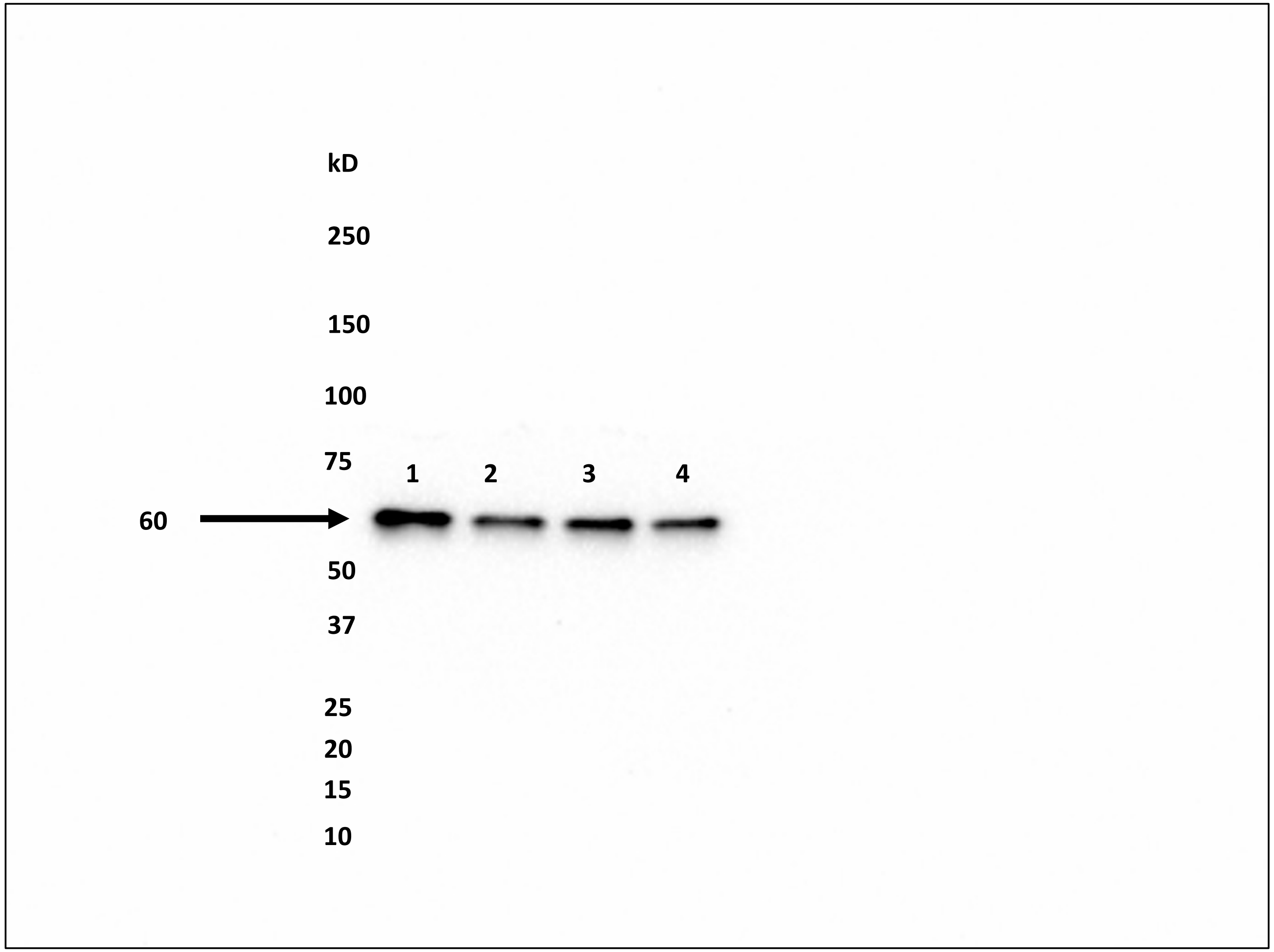

Figure 5E
Probing: α-Tubulin (T9026)
Millipore Sigma

Loading order:
Lane 1: Control at 72 h
Lane 2: 24h 7mM DHA
Lane 3: 48h 7mM DHA
Lane 4: 72 h 7mM DHA

Imaged with Bio-Rad ChemiDoc XRS
Imaging system
Chemi Hi Sensitivity setting

Note: Non-specific bands from previous
antibody probing

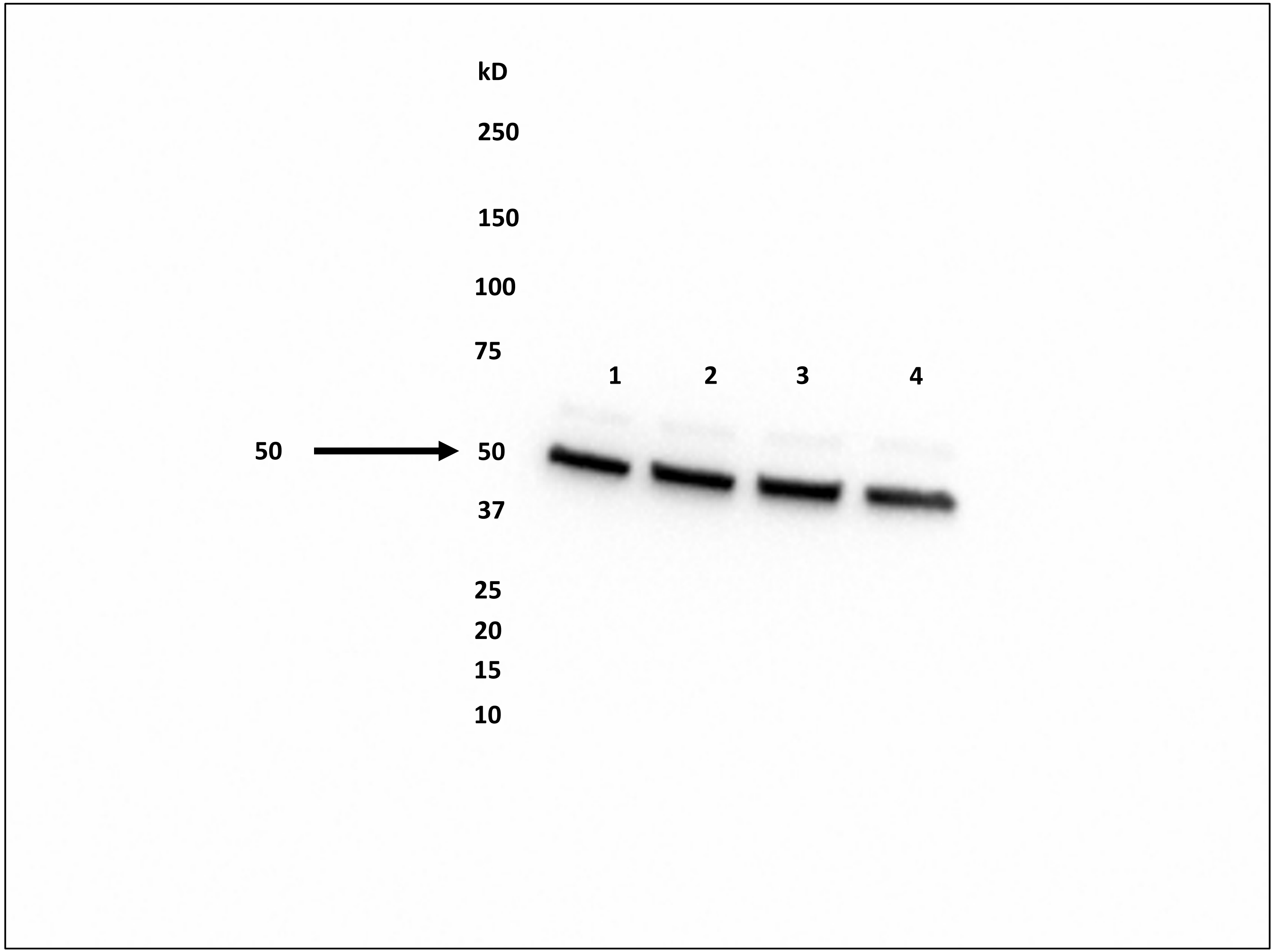

Figure 5F
Probing: p-AKT (Ser473)(D9E) (4060)
Cell Signaling Technologies

Loading order:
Lane 1: Control at 24 h
Lane 2: 1h 7mM DHA
Lane 3: 4h 7mM DHA
Lane 4: 24 h 7mM DHA

Imaged with Bio-Rad ChemiDoc XRS
Imaging system
Chemi Hi Sensitivity setting

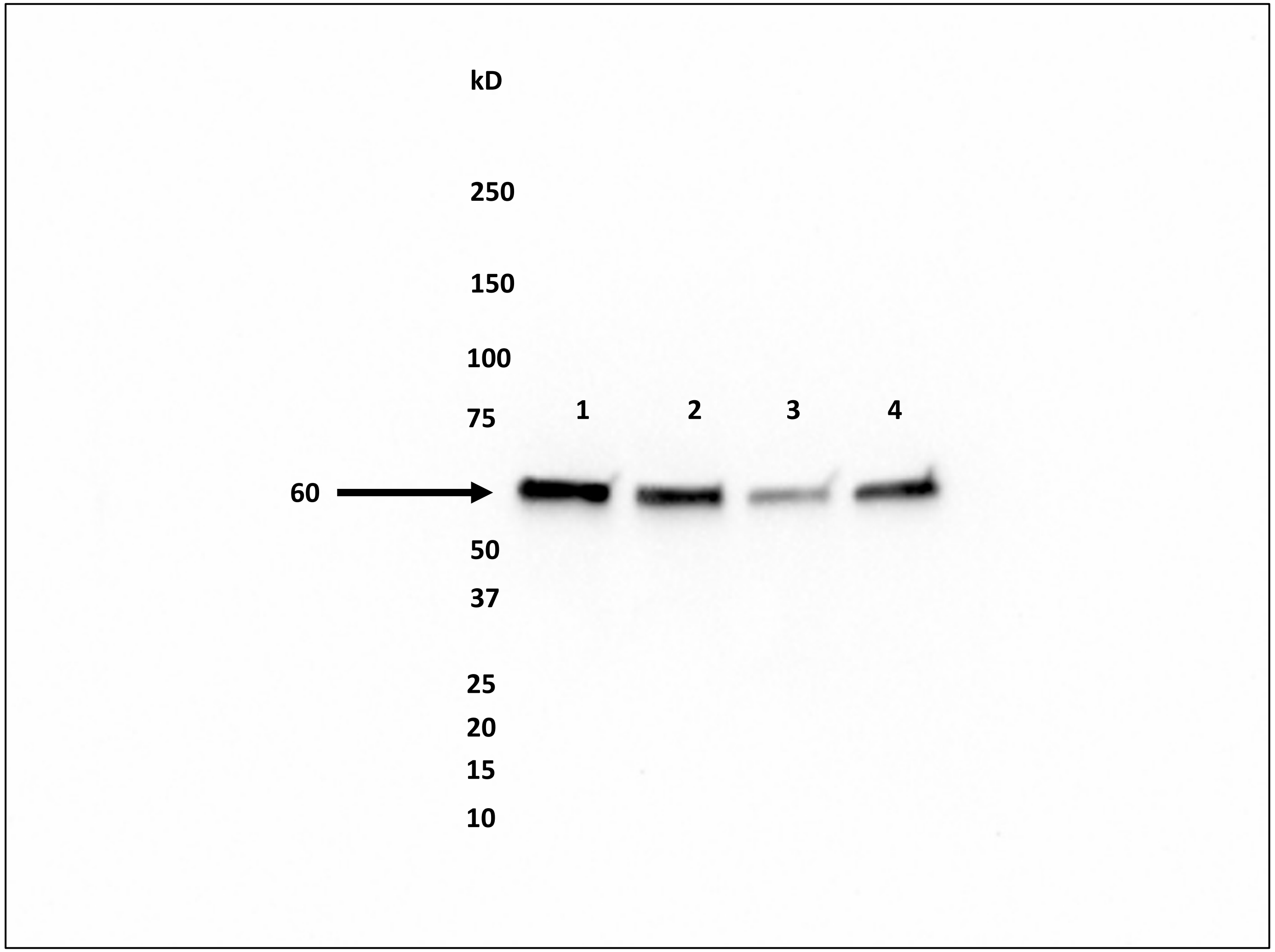

Figure 5F
Probing: AKT (C67E7) (4691)
Cell Signaling Technologies

Loading order:
Lane 1: Control at 24 h
Lane 2: 1h 7mM DHA
Lane 3: 4h 7mM DHA
Lane 4: 24 h 7mM DHA

Imaged with Bio-Rad ChemiDoc XRS
Imaging system
Chemi Hi Sensitivity setting

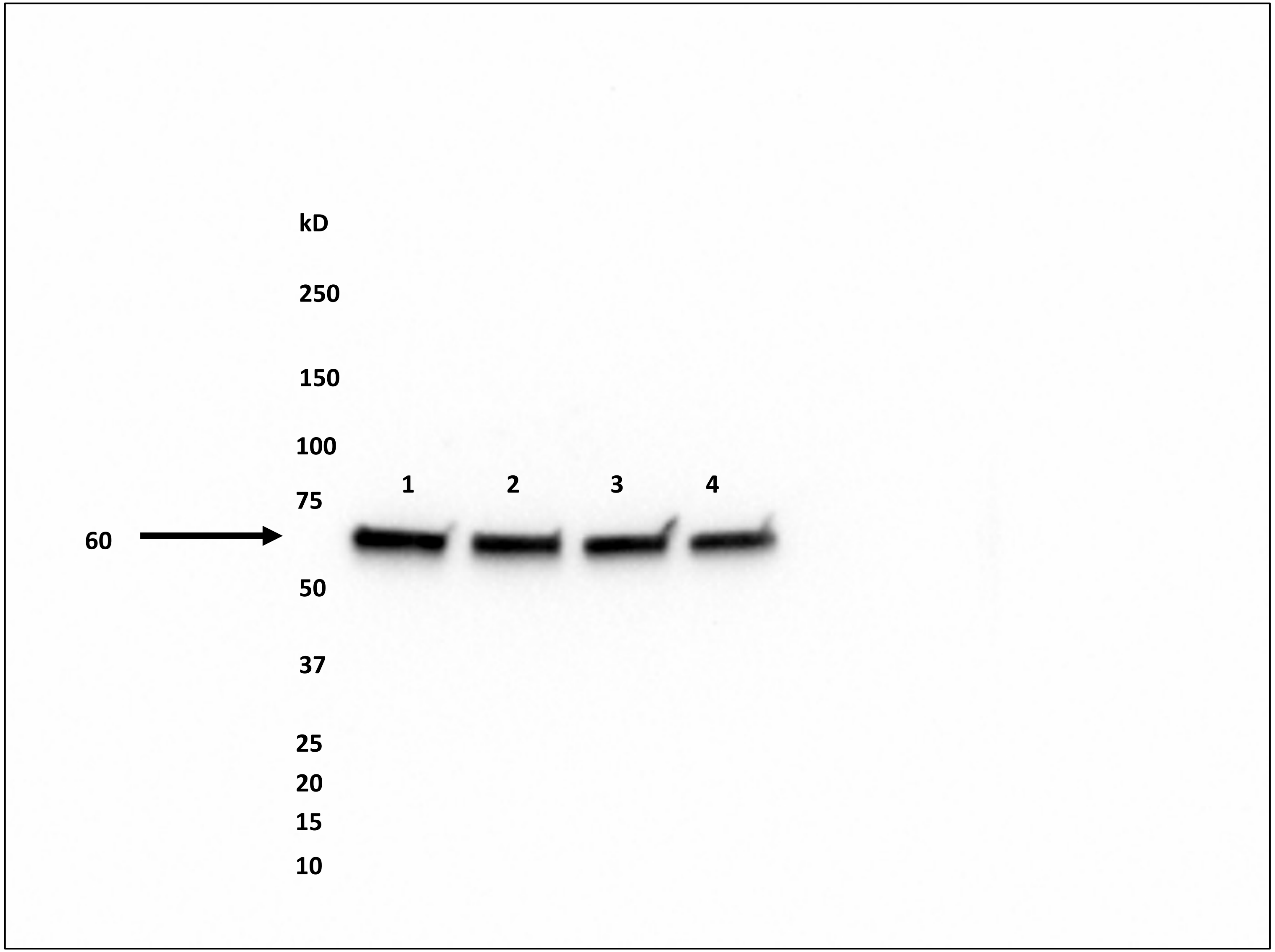

Figure 5F
Probing: α-Tubulin (T9026)
Millipore Sigma

Loading order:
Lane 1: Control at 24 h
Lane 2: 1h 7mM DHA
Lane 3: 4h 7mM DHA
Lane 4: 24 h 7mM DHA

Imaged with Bio-Rad ChemiDoc XRS
Imaging system
Chemi Hi Sensitivity setting

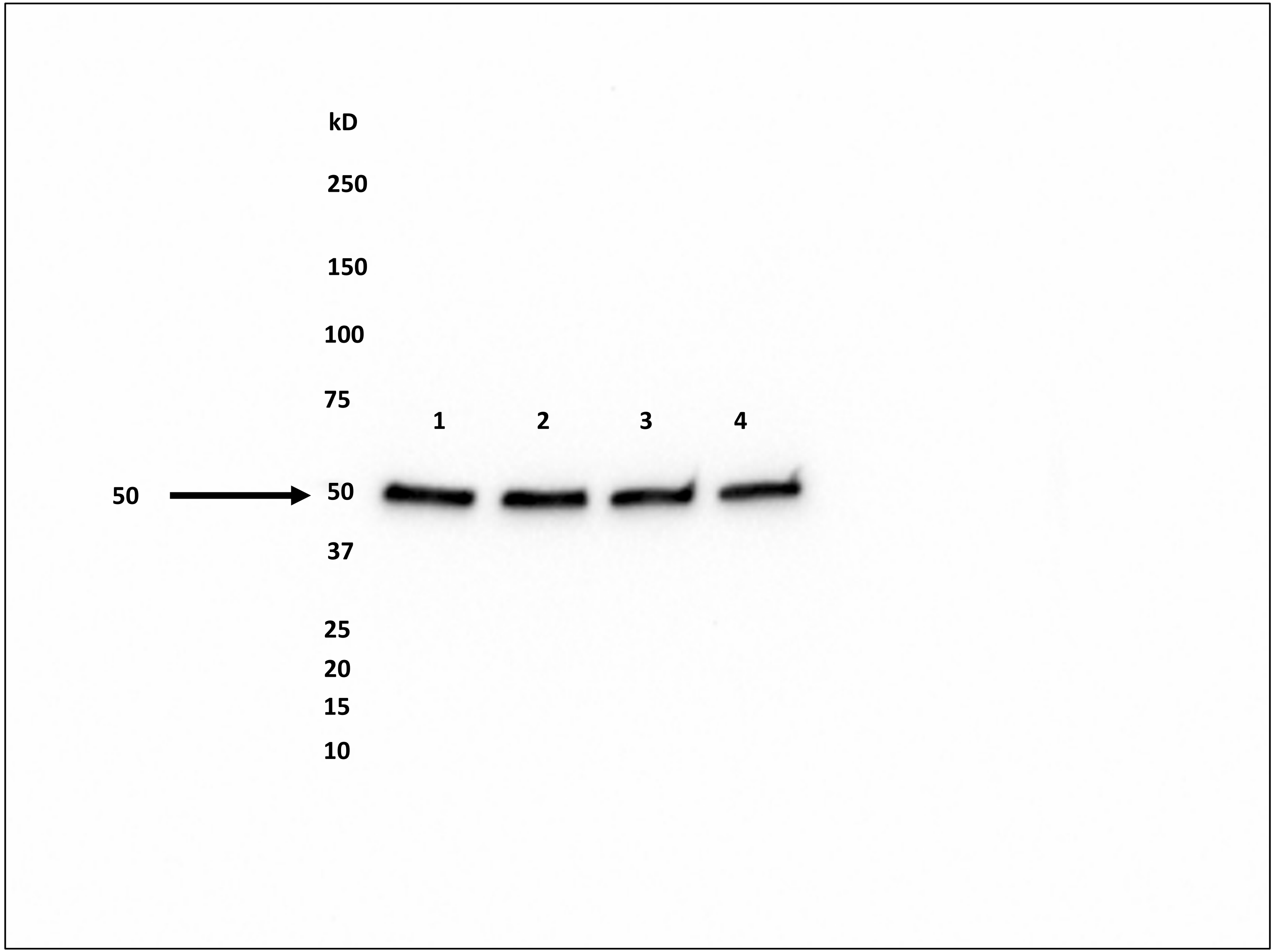

Figure 5G
Probing: p-AMPKα (2535)
Cell Signaling Technologies

Loading order:
Lane 1: Control at 96 h
Lane 2: 24h 7mM DHA
Lane 3: 48h 7mM DHA
Lane 4: 72 h 7mM DHA
Lane 5: 96 h 7 mM DHA

Imaged with Bio-Rad ChemiDoc XRS
Imaging system
Chemi Hi Sensitivity setting

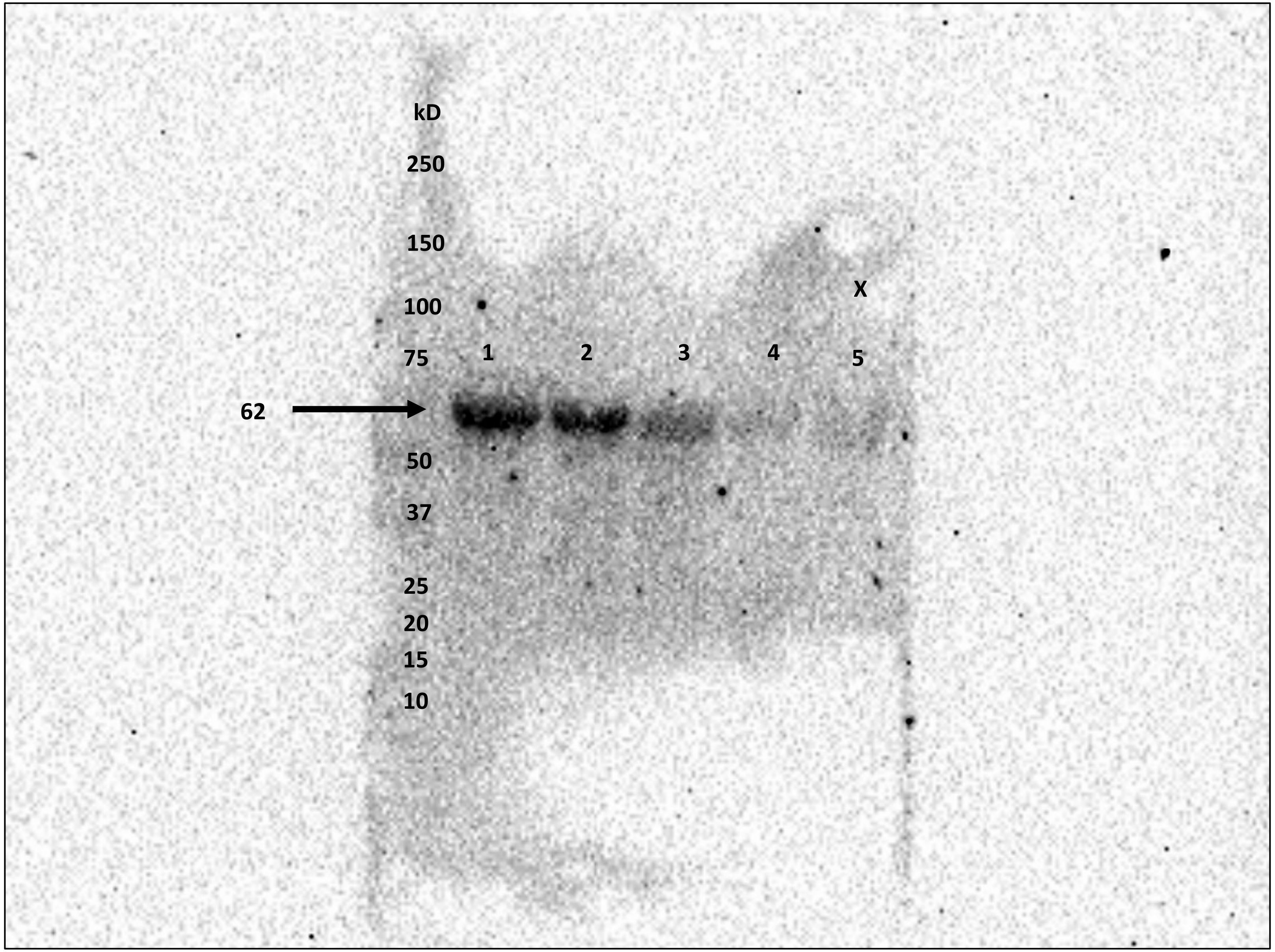

Figure 5G
Probing: AMPKα (2532)
Cell Signaling Technologies

Loading order:
Lane 1: Control at 96 h
Lane 2: 24h 7mM DHA
Lane 3: 48h 7mM DHA
Lane 4: 72 h 7mM DHA
Lane 5: 96 h 7 mM DHA

Imaged with Bio-Rad ChemiDoc XRS
Imaging system
Chemi Hi Sensitivity setting

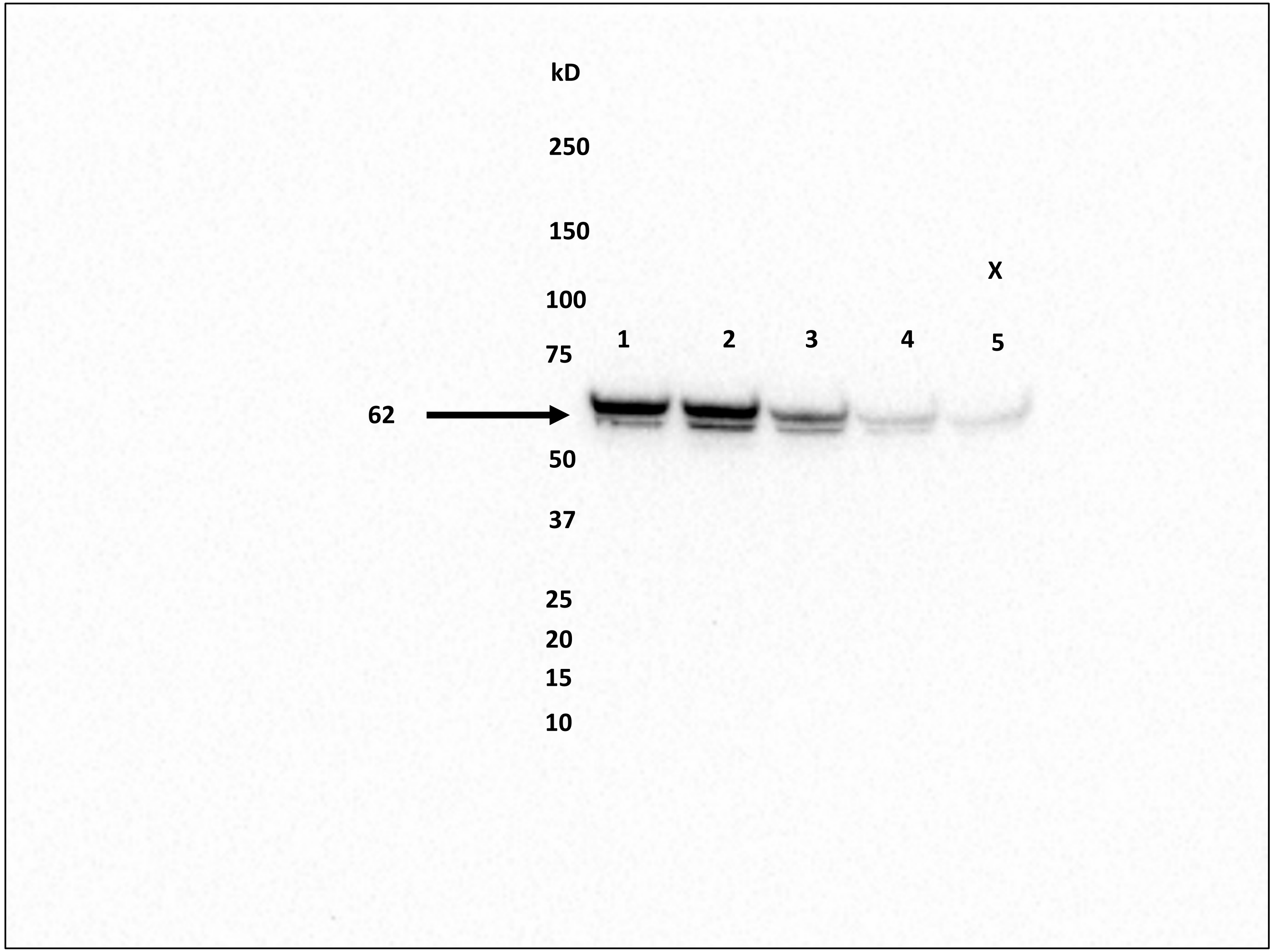

Figure 5G
Probing: α-Tubulin (T9026)
Millipore Sigma

Loading order:
Lane 1: Control at 96 h
Lane 2: 24h 7mM DHA
Lane 3: 48h 7mM DHA
Lane 4: 72 h 7mM DHA
Lane 5: 96 h 7 mM DHA

Imaged with Bio-Rad ChemiDoc XRS
Imaging system
Chemi Hi Sensitivity setting

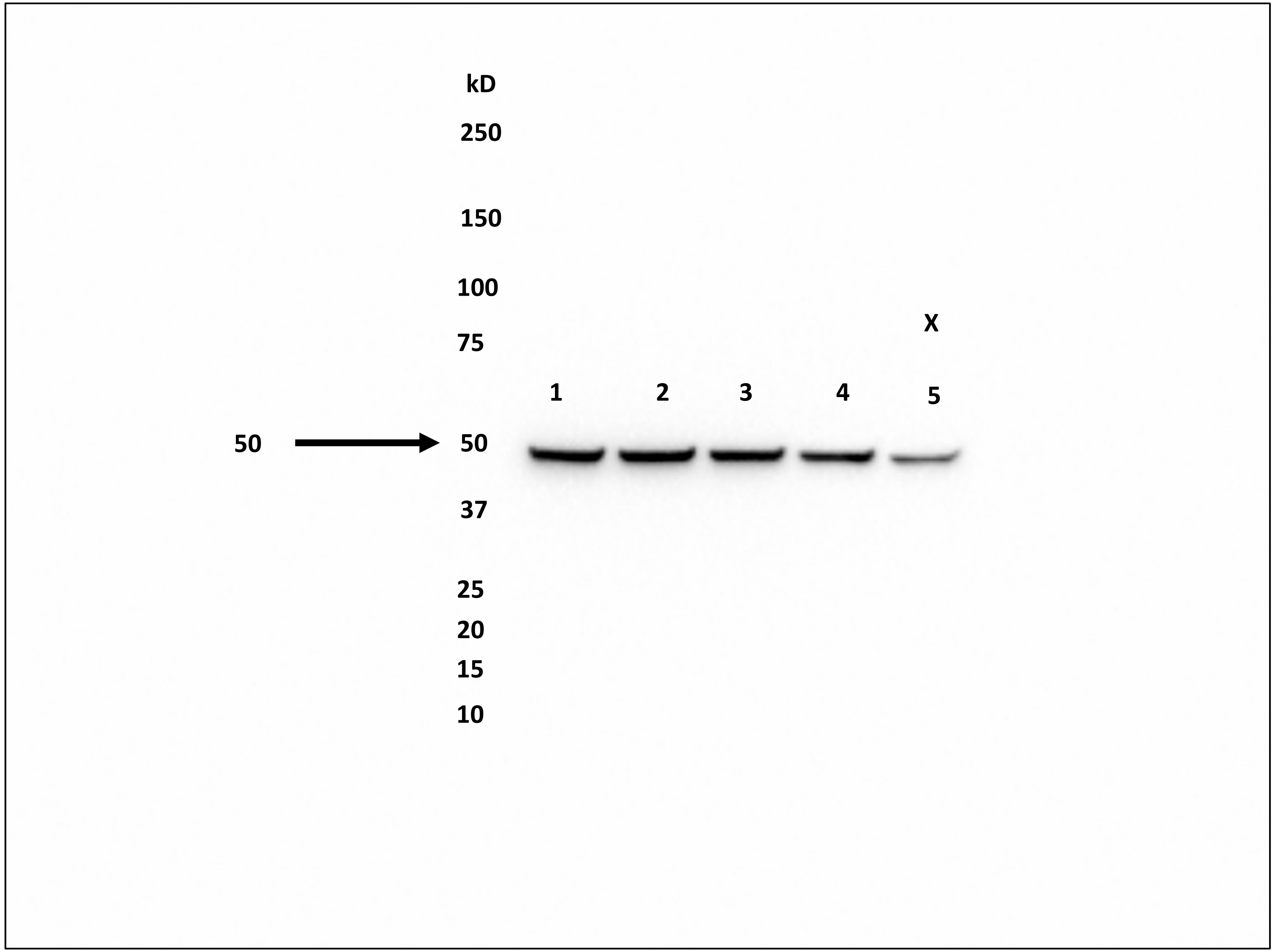

Figure 5H
Probing: p-AMPKα (2535)
Cell Signaling Technologies

Loading order:
Lane 1: Control at 24 h
Lane 2: 1h 7mM DHA
Lane 3: 4h 7mM DHA
Lane 4: 24 h 7mM DHA
Lane 5: Control at 24 h

Imaged with Bio-Rad ChemiDoc XRS
Imaging system
Chemi Hi Sensitivity setting

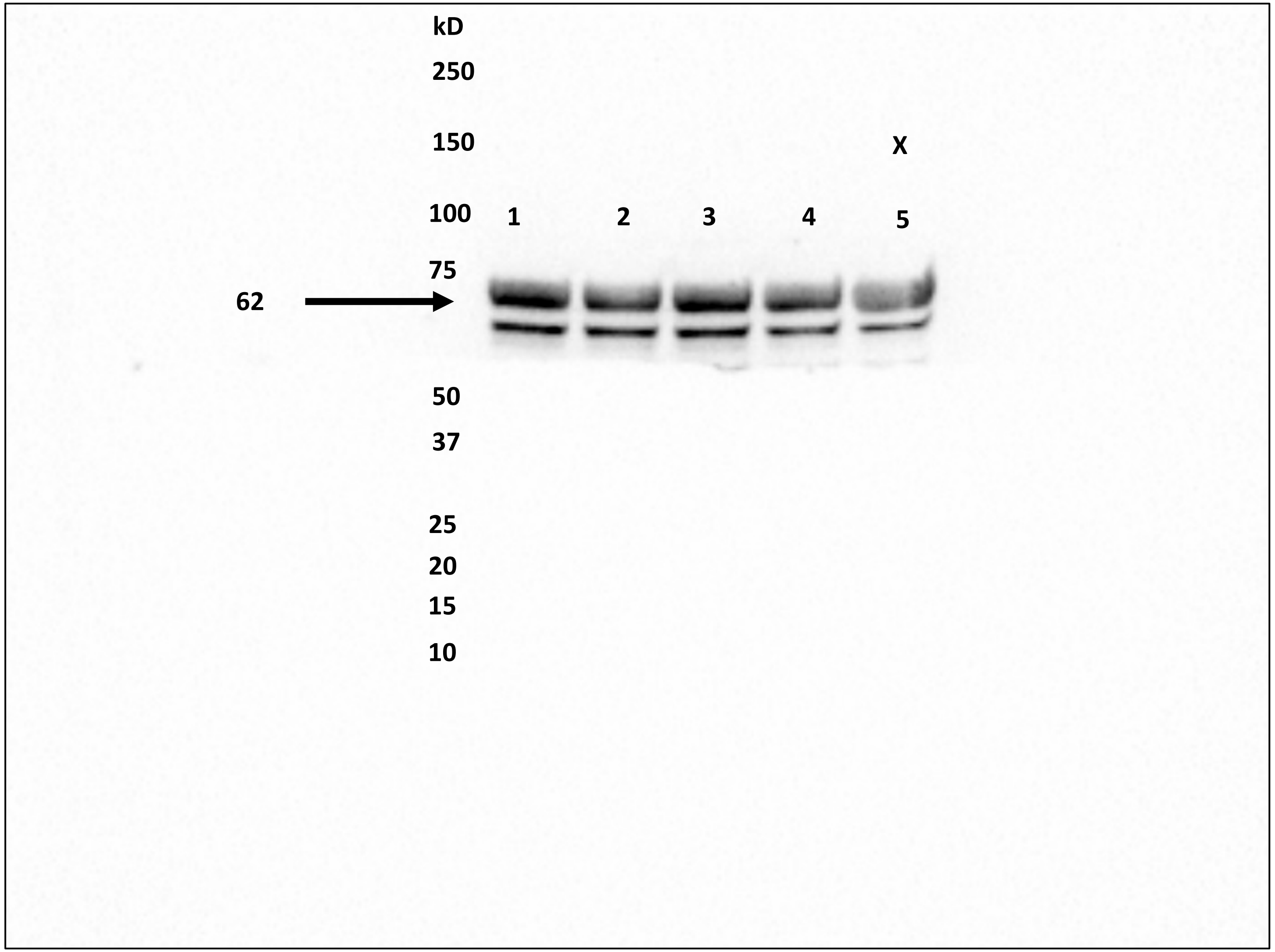

Figure 5H
Probing: p-AMPKα (2535)
Cell Signaling Technologies

Loading order:
Lane 1: Control at 24 h
Lane 2: 1h 7mM DHA
Lane 3: 4h 7mM DHA
Lane 4: 24 h 7mM DHA
Lane 5: Control at 24 h

Imaged with Bio-Rad ChemiDoc XRS
Imaging system
Chemi Hi Sensitivity setting

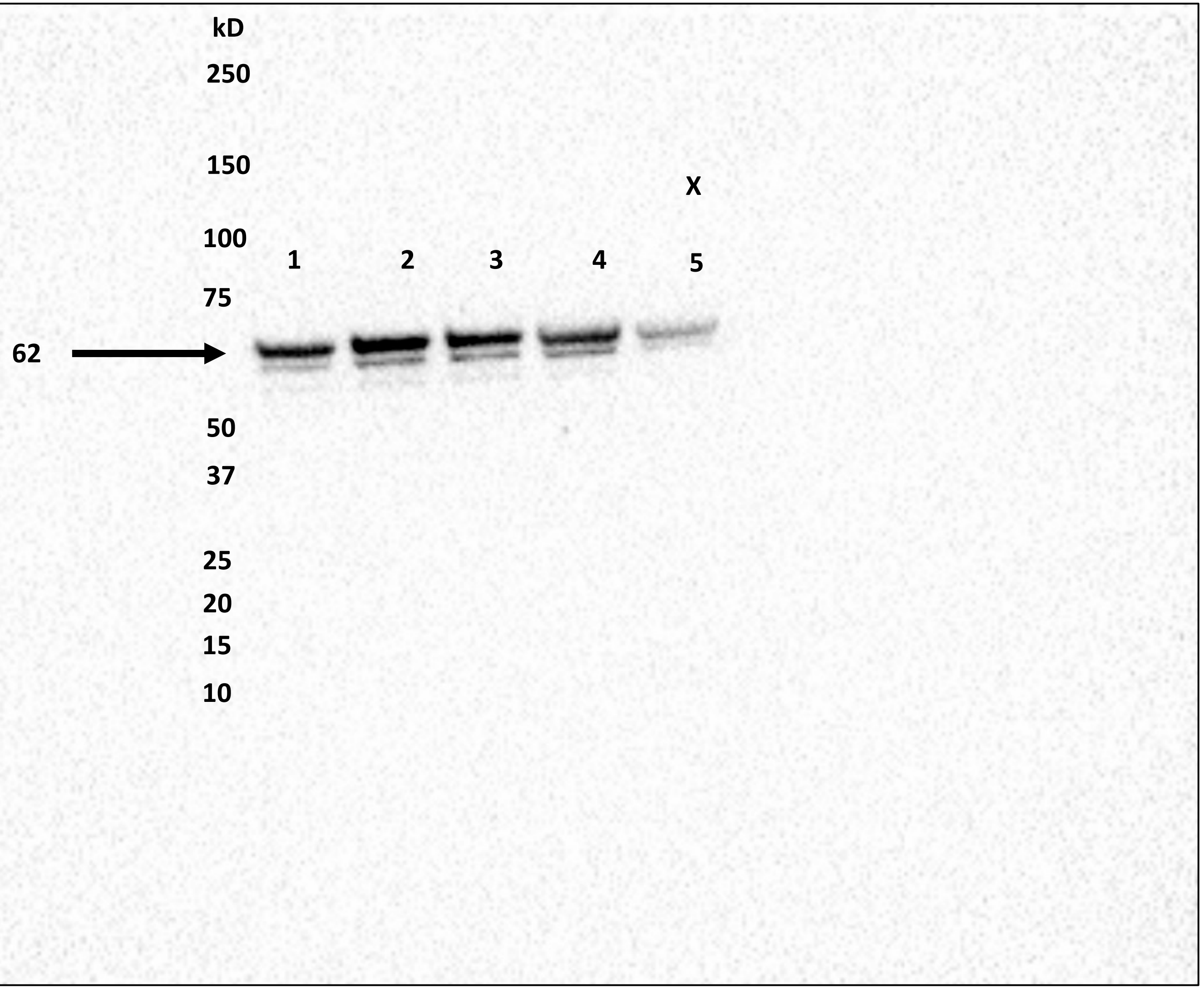

Figure 5H
Probing: α-Tubulin (T9026)
Millipore Sigma

Loading order:
Lane 1: Control at 24 h
Lane 2: 1h 7mM DHA
Lane 3: 4h 7mM DHA
Lane 4: 24 h 7mM DHA
Lane 5: Control at 24 h

Imaged with Bio-Rad ChemiDoc XRS
Imaging system
Chemi Hi Sensitivity setting

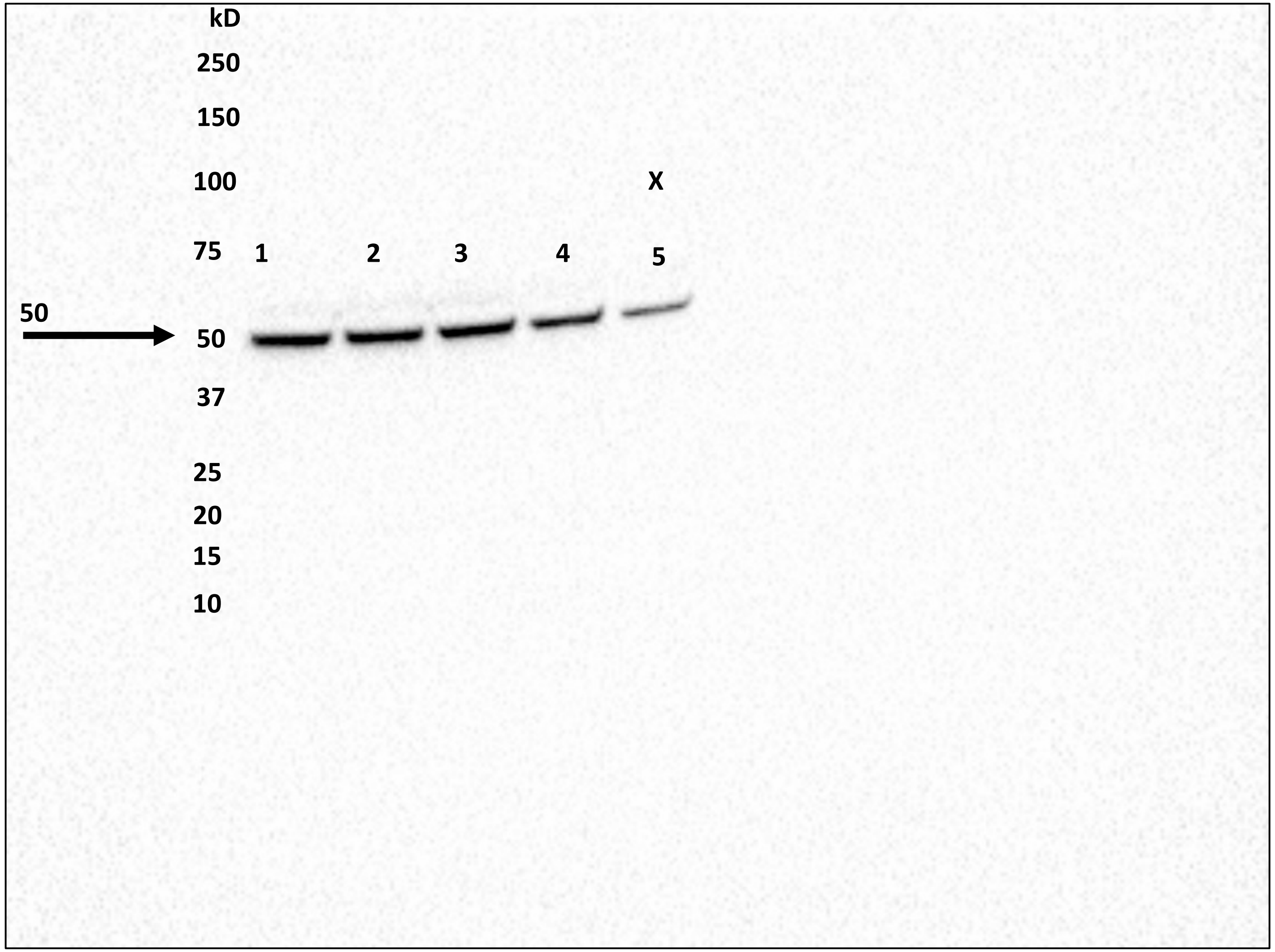

S3A Fig
Probing: Caspase-1 (D7F10) (3866)
Cell Signaling Technologies

Loading order:
Lane 1: Control at 96 h
Lane 2: 48h 7mM DHA
Lane 3: 72 h 7mM DHA
Lane 4: 96 h 7 mM DHA

Imaged with Bio-Rad ChemiDoc XRS
Imaging system
Chemi Hi Sensitivity setting

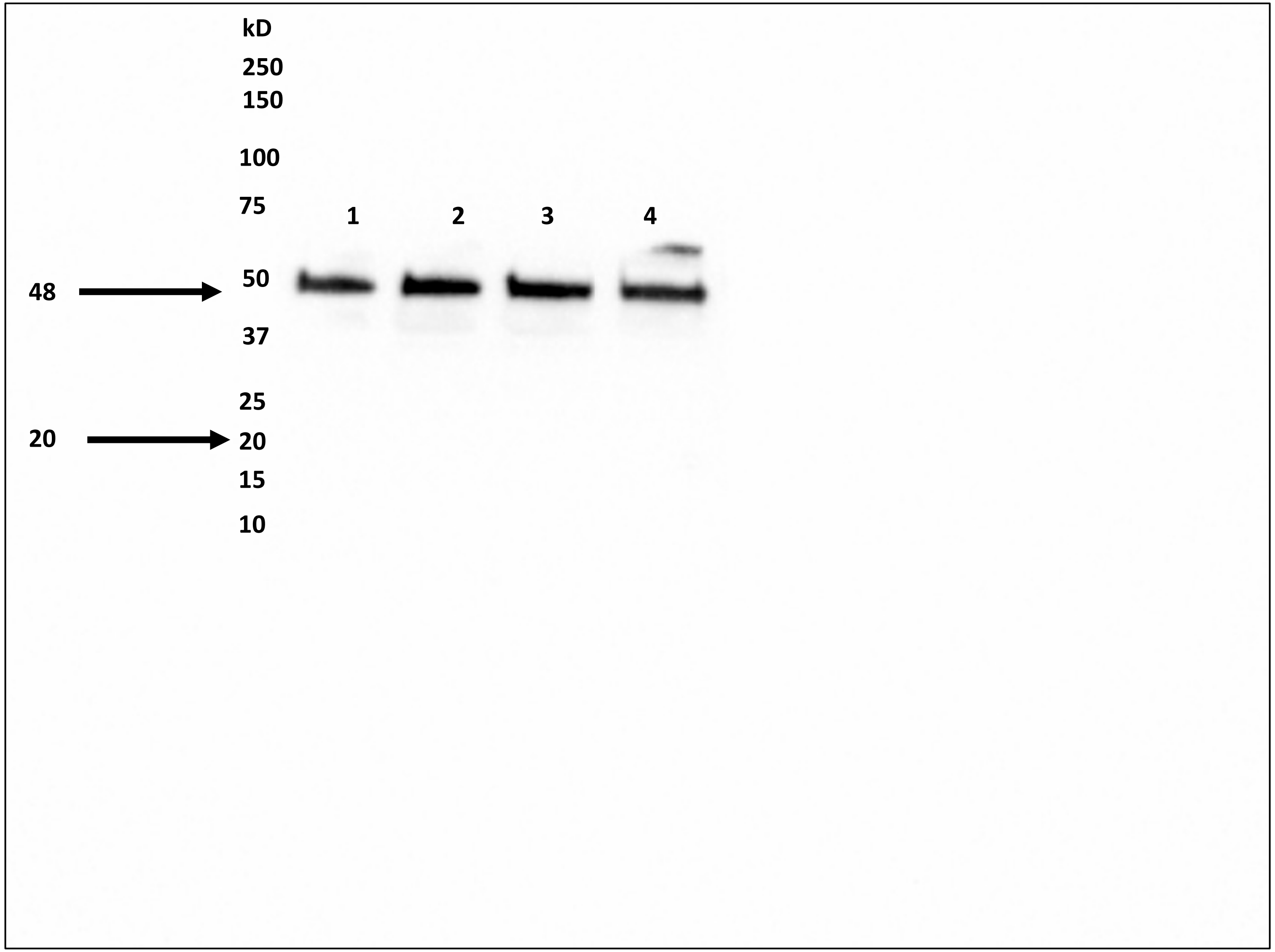

S3A Fig
Probing: α-Tubulin (T9026)
Millipore Sigma

Loading order:
Lane 1: Control at 96 h
Lane 2: 48h 7mM DHA
Lane 3: 72 h 7mM DHA
Lane 4: 96 h 7 mM DHA

Imaged with Bio-Rad ChemiDoc XRS
Imaging system
Chemi Hi Sensitivity setting

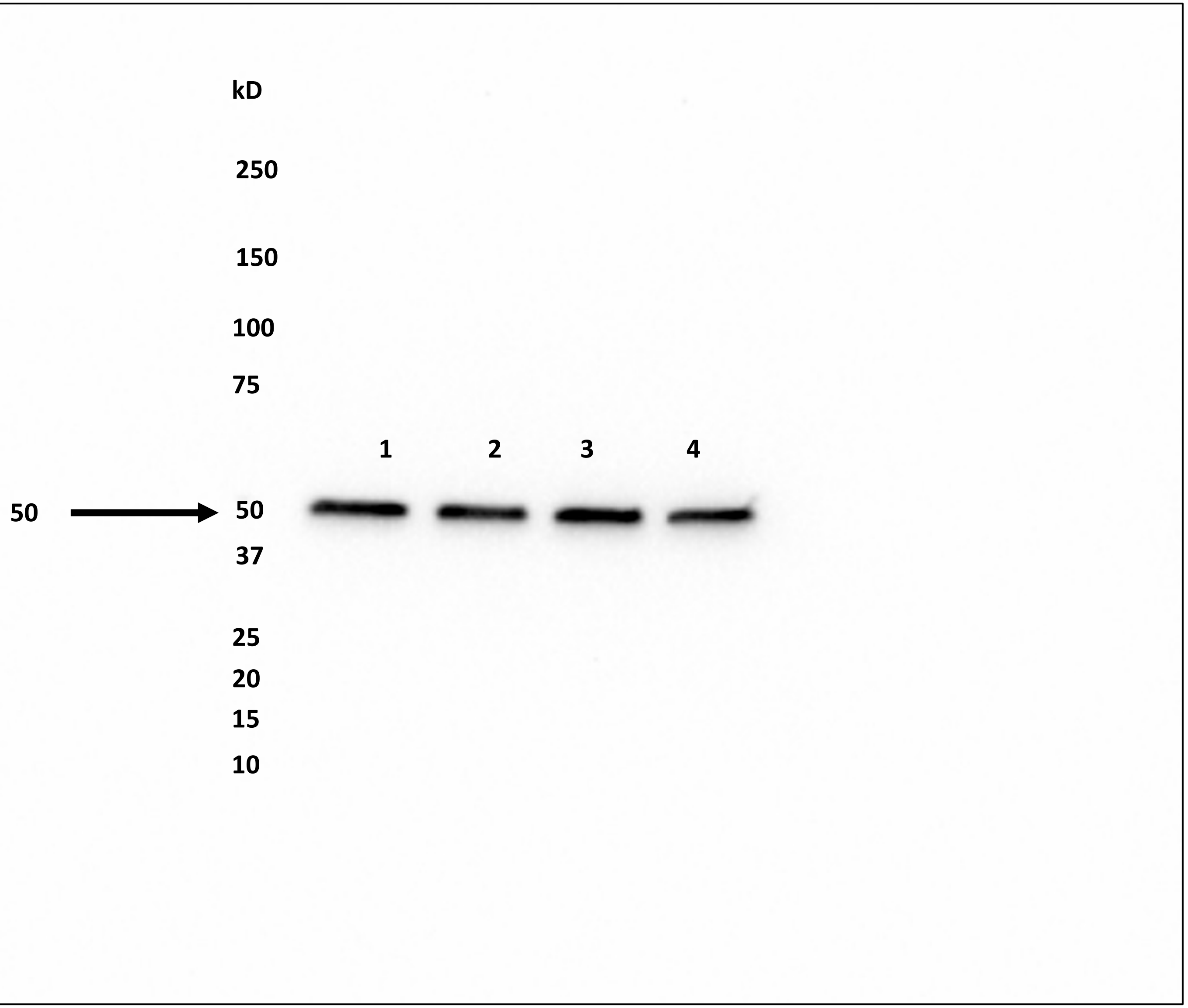

S3A Fig
Probing: Caspase-2 (C2) (2224)
Cell Signaling Technologies

Loading order:
Lane 1: Control at 96 h
Lane 2: 48h 7mM DHA
Lane 3: 72 h 7mM DHA
Lane 4: 96 h 7 mM DHA

Imaged with Bio-Rad ChemiDoc XRS
Imaging system
Chemi Hi Sensitivity setting

Note: Non-specific bands from previous antibody probing

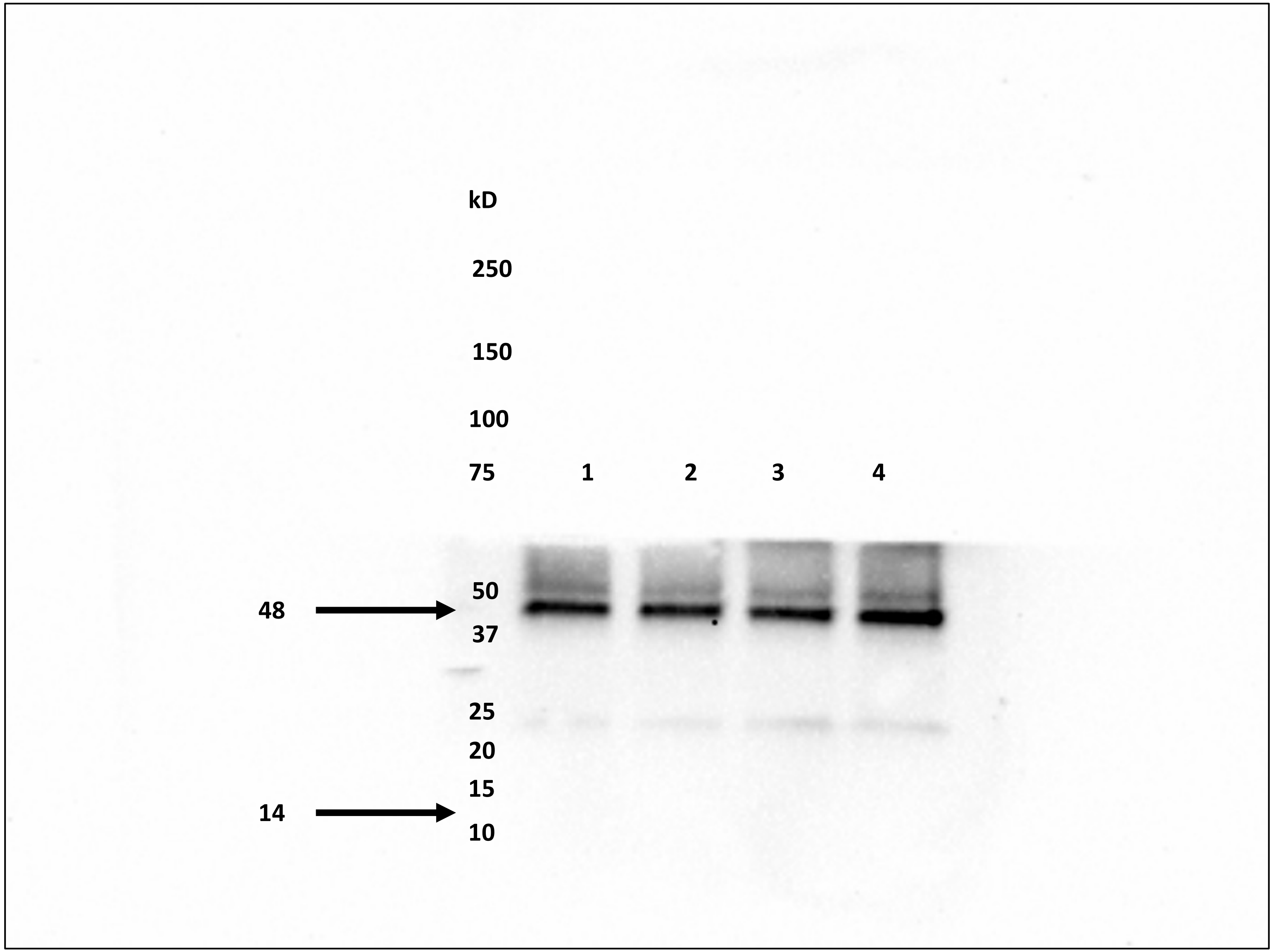

S3A Fig
Probing: α-Tubulin (T9026)
Millipore Sigma

Loading order:
Lane 1: Control at 96 h
Lane 2: 48h 7mM DHA
Lane 3: 72 h 7mM DHA
Lane 4: 96 h 7 mM DHA

Imaged with Bio-Rad ChemiDoc XRS
Imaging system
Chemi Hi Sensitivity setting

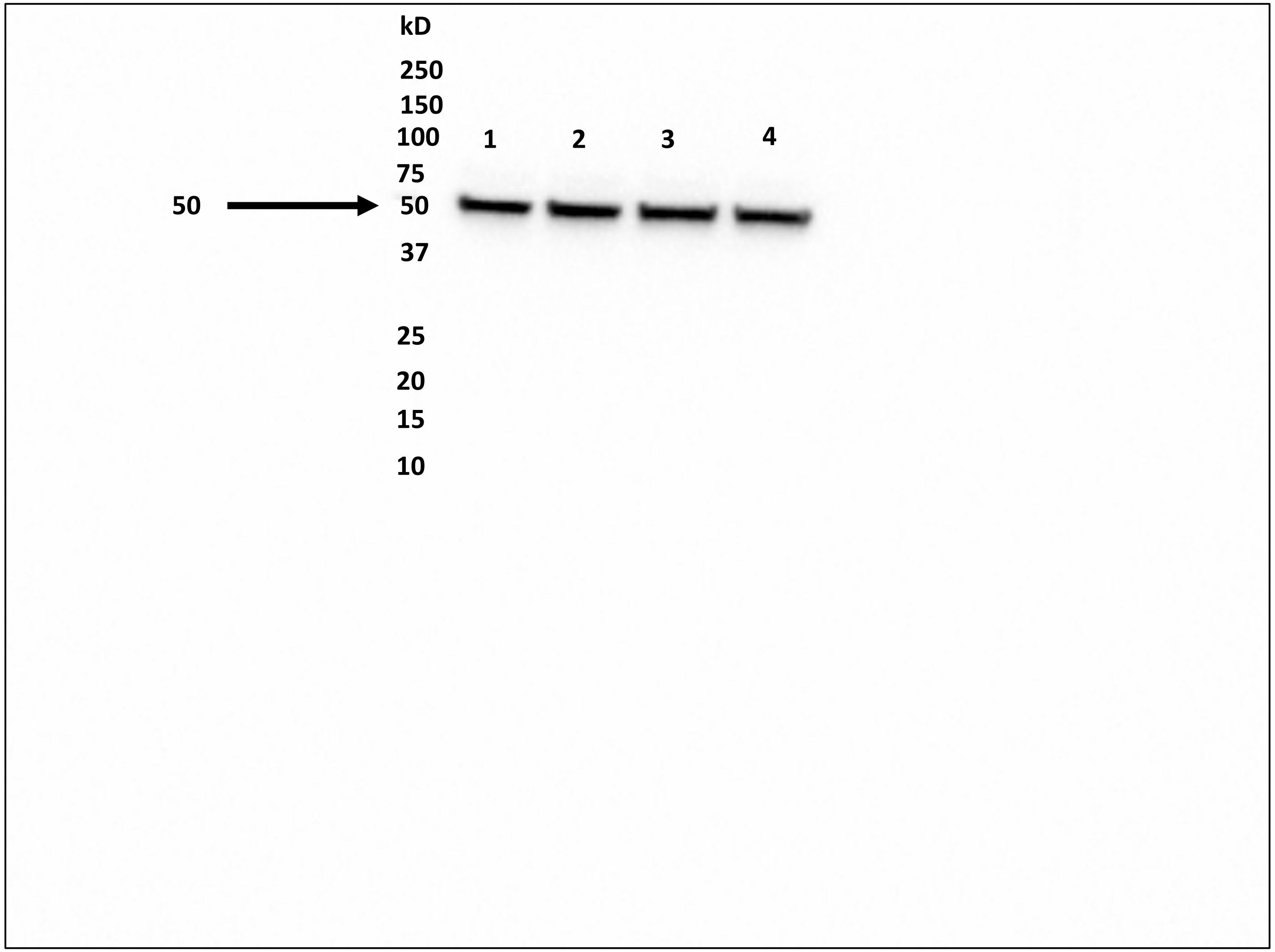

S3B Fig
Probing: Caspase-3 (GTX13585)
GeneTex

Loading order:
Lane 1: Control at 96 h
Lane 2: 24h 7mM DHA
Lane 3: 48h 7mM DHA
Lane 4: 72 h 7mM DHA
Lane 5: 96 h 7 mM DHA

Imaged with Bio-Rad ChemiDoc XRS
Imaging system
Chemi Hi Sensitivity setting

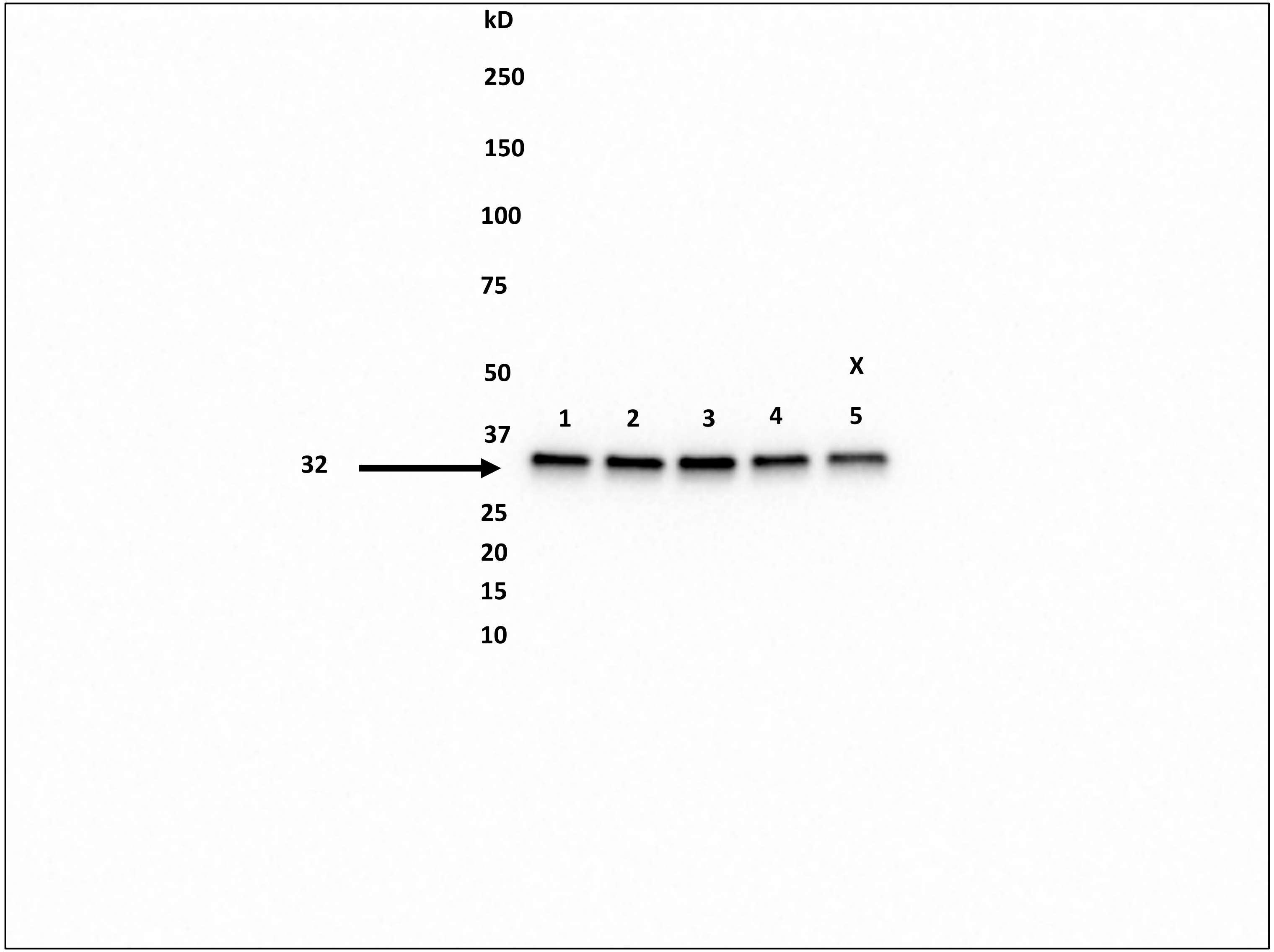

S3B Fig
Probing: α-Tubulin (T9026)
Millipore Sigma

Loading order:
Lane 1: Control at 96 h
Lane 2: 24h 7mM DHA
Lane 3: 48h 7mM DHA
Lane 4: 72 h 7mM DHA
Lane 5: 96 h 7 mM DHA

Imaged with Bio-Rad ChemiDoc XRS
Imaging system
Chemi Hi Sensitivity setting

Note: Non-specific bands from previous
antibody probing

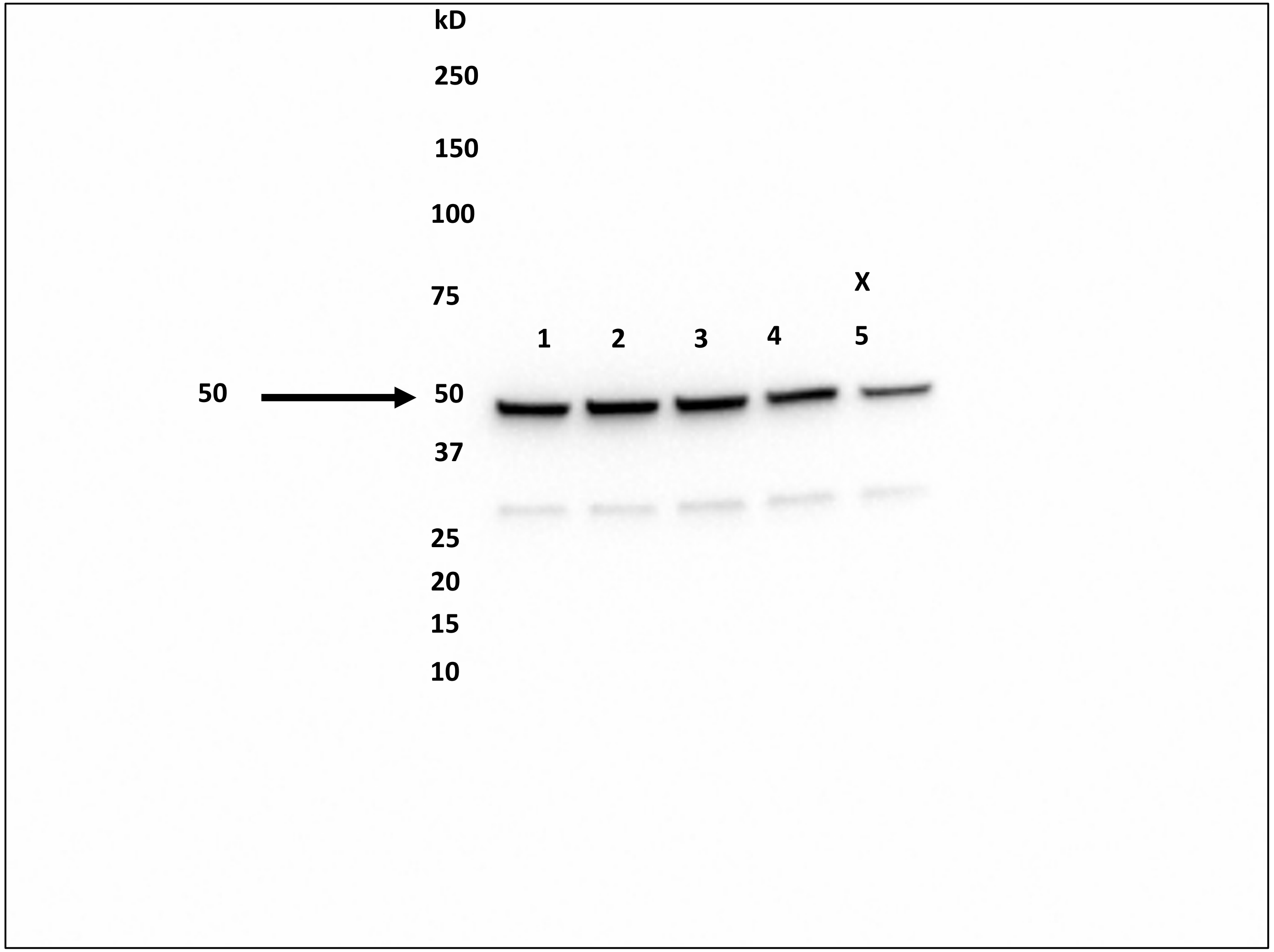

S3B Fig
Probing: Cathepsin L (71298)
Cell Signaling Technologies

Loading order:
Lane 1: Control at 72 h
Lane 2: 24h 7mM DHA
Lane 3: 48h 7mM DHA
Lane 4: 72 h 7mM DHA

Imaged with Bio-Rad ChemiDoc XRS
Imaging system
Chemi Hi Sensitivity setting

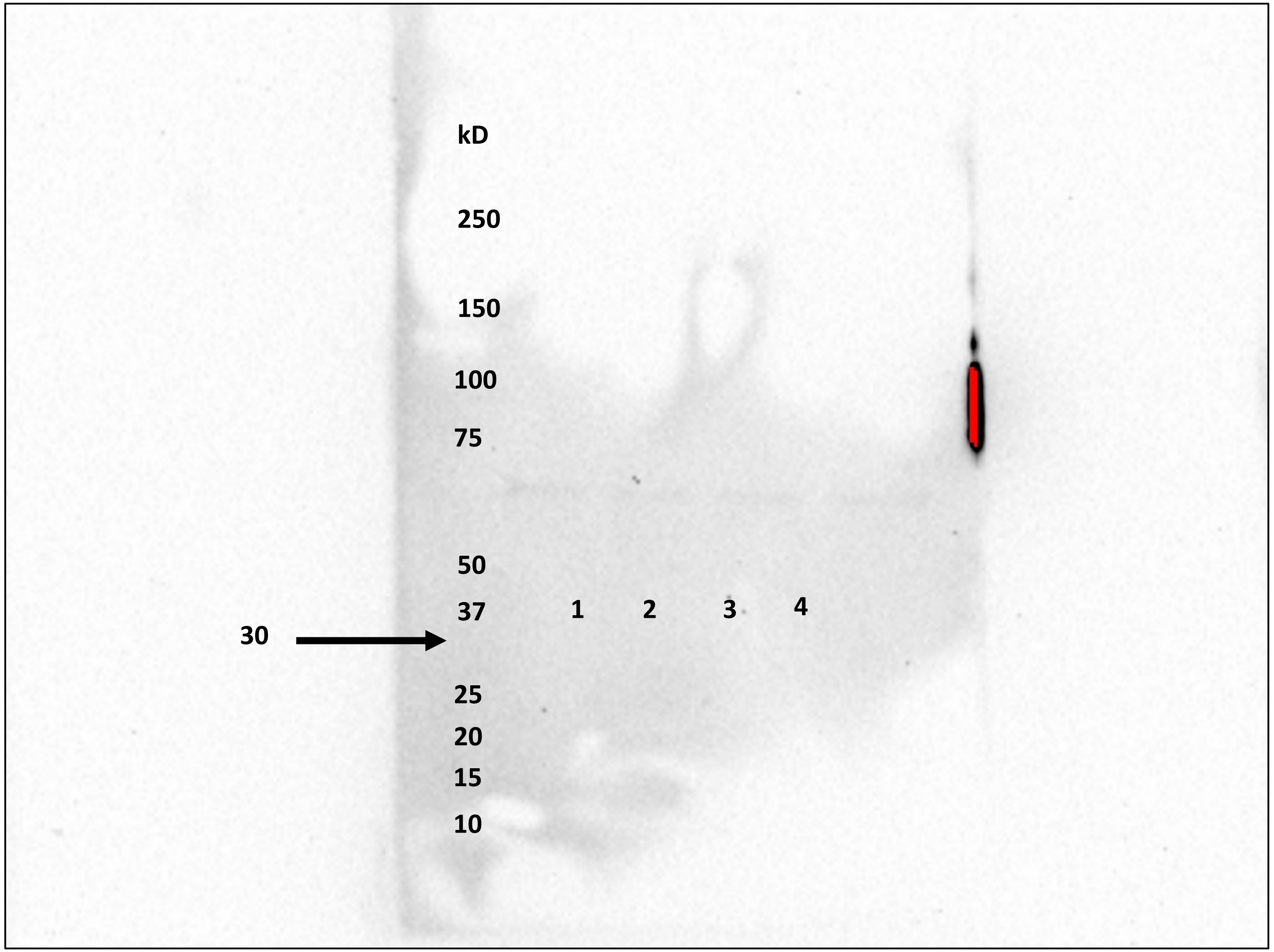

S3B Fig
Probing: α-Tubulin (T9026)
Millipore Sigma

Loading order:
Lane 1: Control at 72 h
Lane 2: 24h 7mM DHA
Lane 3: 48h 7mM DHA
Lane 4: 72 h 7mM DHA

Imaged with Bio-Rad ChemiDoc XRS
Imaging system
Chemi Hi Sensitivity setting

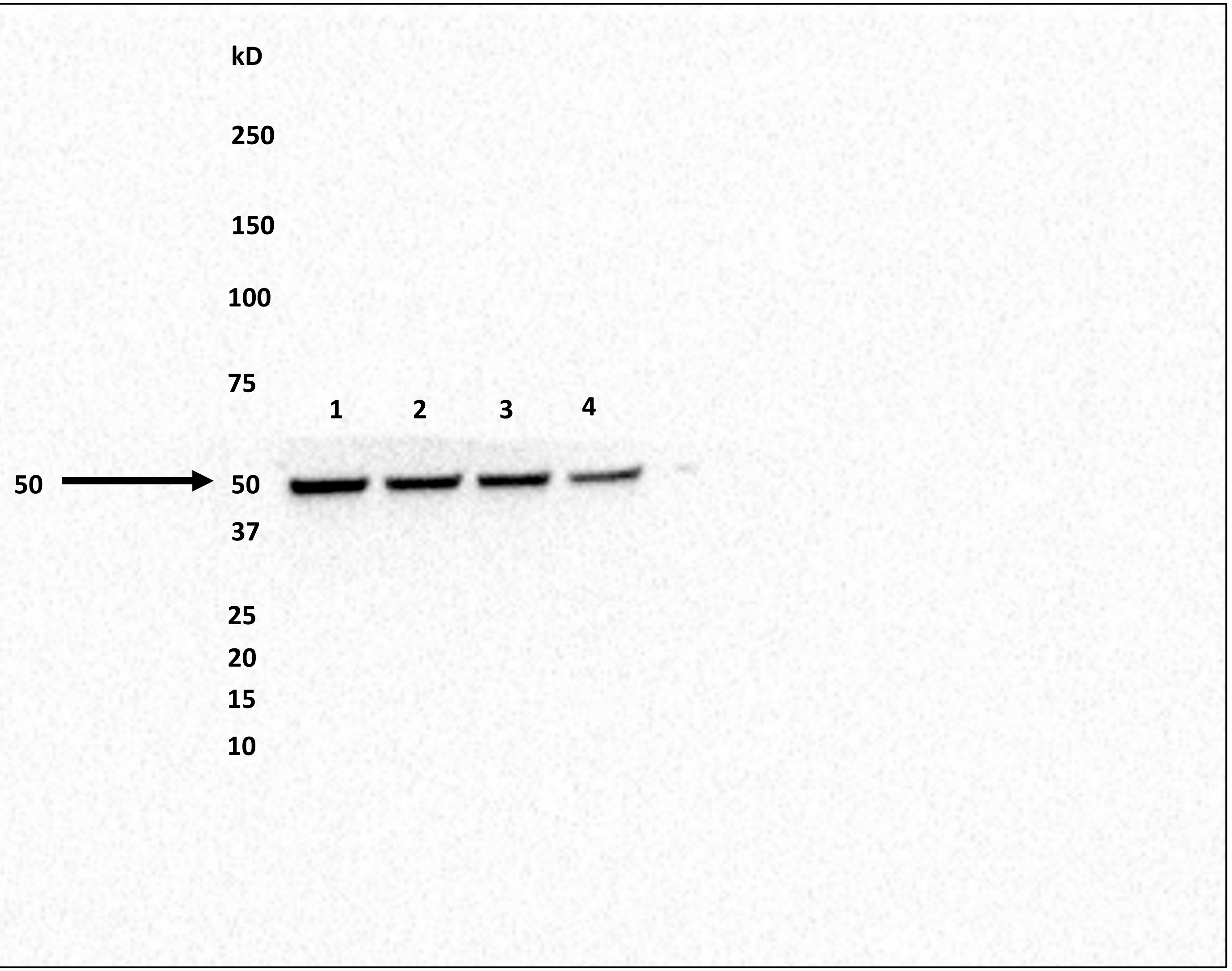

S5B Fig
Probing: p-mTOR (Ser2448) (2972)
Cell Signaling Technologies

Loading order:
Lane 1: Control at 72 h
Lane 2: 24h 7mM DHA
Lane 3: 48h 7mM DHA
Lane 4: 72 h 7mM DHA
Lane 5: 24h 10 nM Rapamycin
Lane 6: 48h 10 nM Rapamycin
Lane 7: 72h 10 nM Rapamycin
Lane 8: 24h 7 mM DHA and 10 nM Rapamycin
Lane 9: 48h 7 mM DHA and 10 nM Rapamycin
Lane 10: 72h 7 mM DHA and 10 nM Rapamycin

Imaged with Bio-Rad ChemiDoc XRS Imaging system
Chemi Hi Sensitivity setting

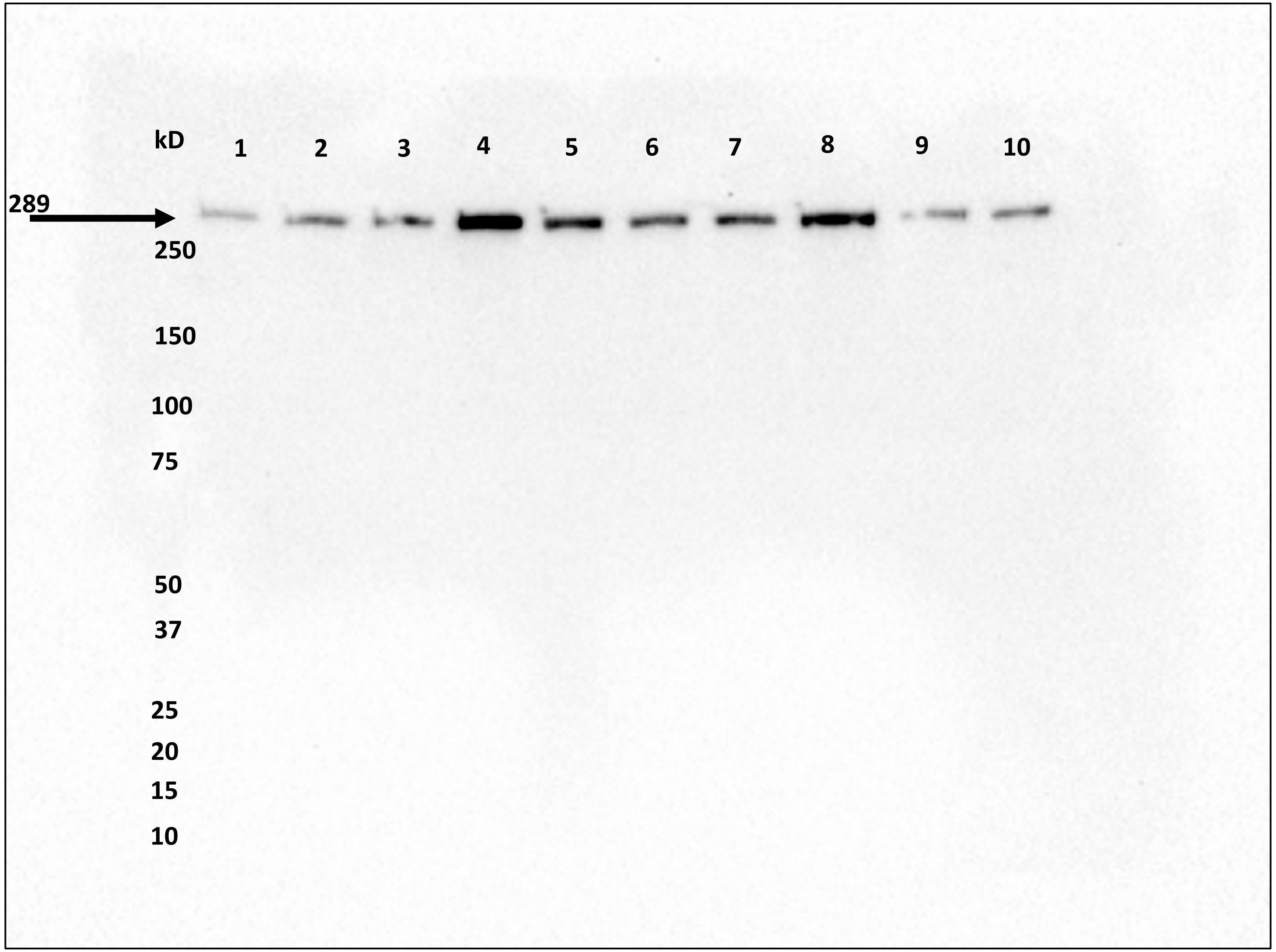

S5B Fig
Probing: mTOR (2972)
Cell Signaling Technologies

Loading order:
Lane 1: Control at 72 h
Lane 2: 24h 7mM DHA
Lane 3: 48h 7mM DHA
Lane 4: 72 h 7mM DHA
Lane 5: 24h 10 nM Rapamycin
Lane 6: 48h 10 nM Rapamycin
Lane 7: 72h 10 nM Rapamycin
Lane 8: 24h 7 mM DHA and 10 nM Rapamycin
Lane 9: 48h 7 mM DHA and 10 nM Rapamycin
Lane 10: 72h 7 mM DHA and 10 nM Rapamycin

Imaged with Bio-Rad ChemiDoc XRS Imaging system
Chemi Hi Sensitivity setting

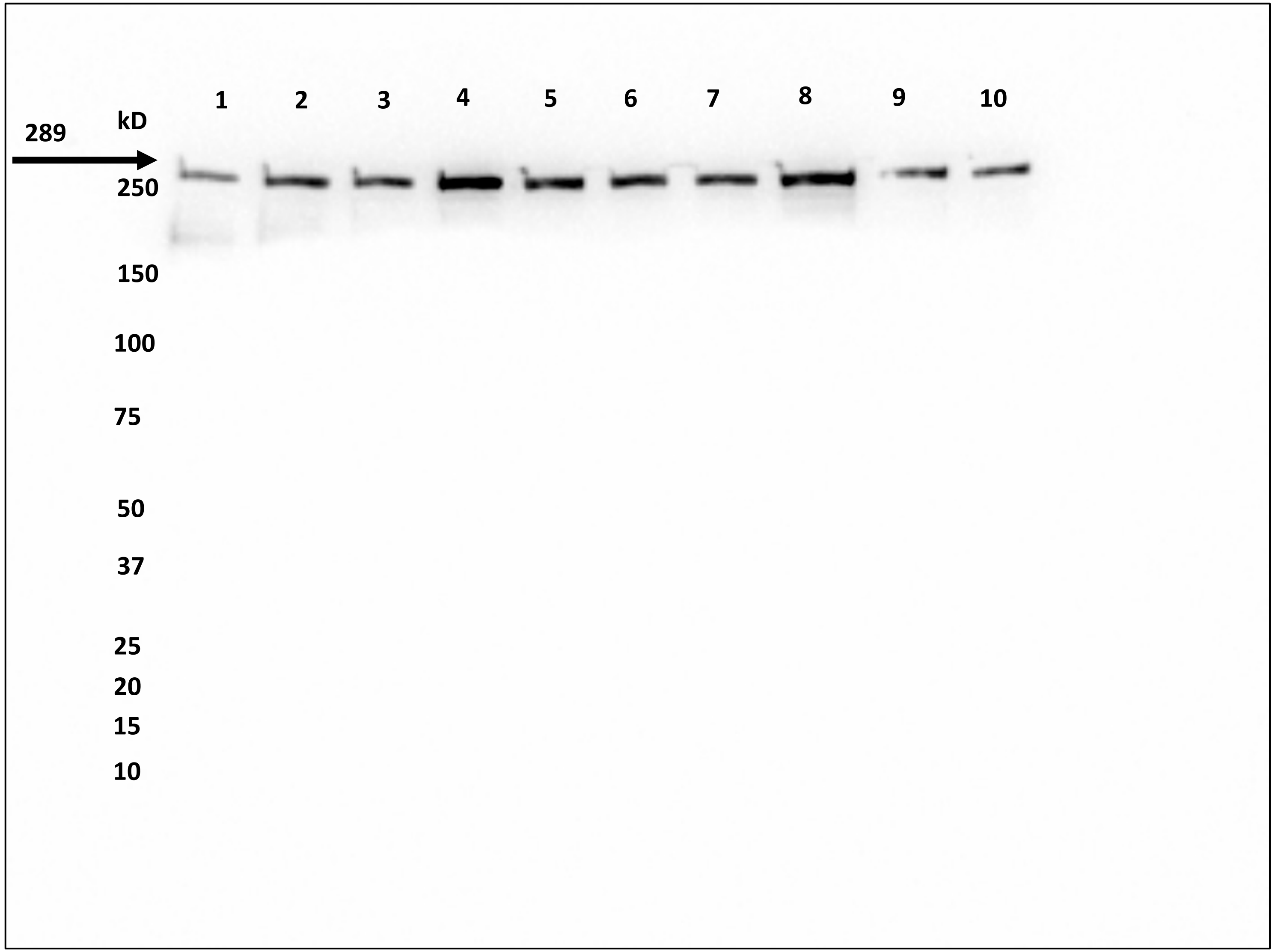

S5B Fig
Probing: α-Tubulin (T9026)
Millipore Sigma

Loading order:
Lane 1: Control at 72 h
Lane 2: 24h 7mM DHA
Lane 3: 48h 7mM DHA
Lane 4: 72 h 7mM DHA
Lane 5: 24h 10 nM Rapamycin
Lane 6: 48h 10 nM Rapamycin
Lane 7: 72h 10 nM Rapamycin
Lane 8: 24h 7 mM DHA and 10 nM Rapamycin
Lane 9: 48h 7 mM DHA and 10 nM Rapamycin
Lane 10: 72h 7 mM DHA and 10 nM Rapamycin

Imaged with Bio-Rad ChemiDoc XRS Imaging system
Chemi Hi Sensitivity setting

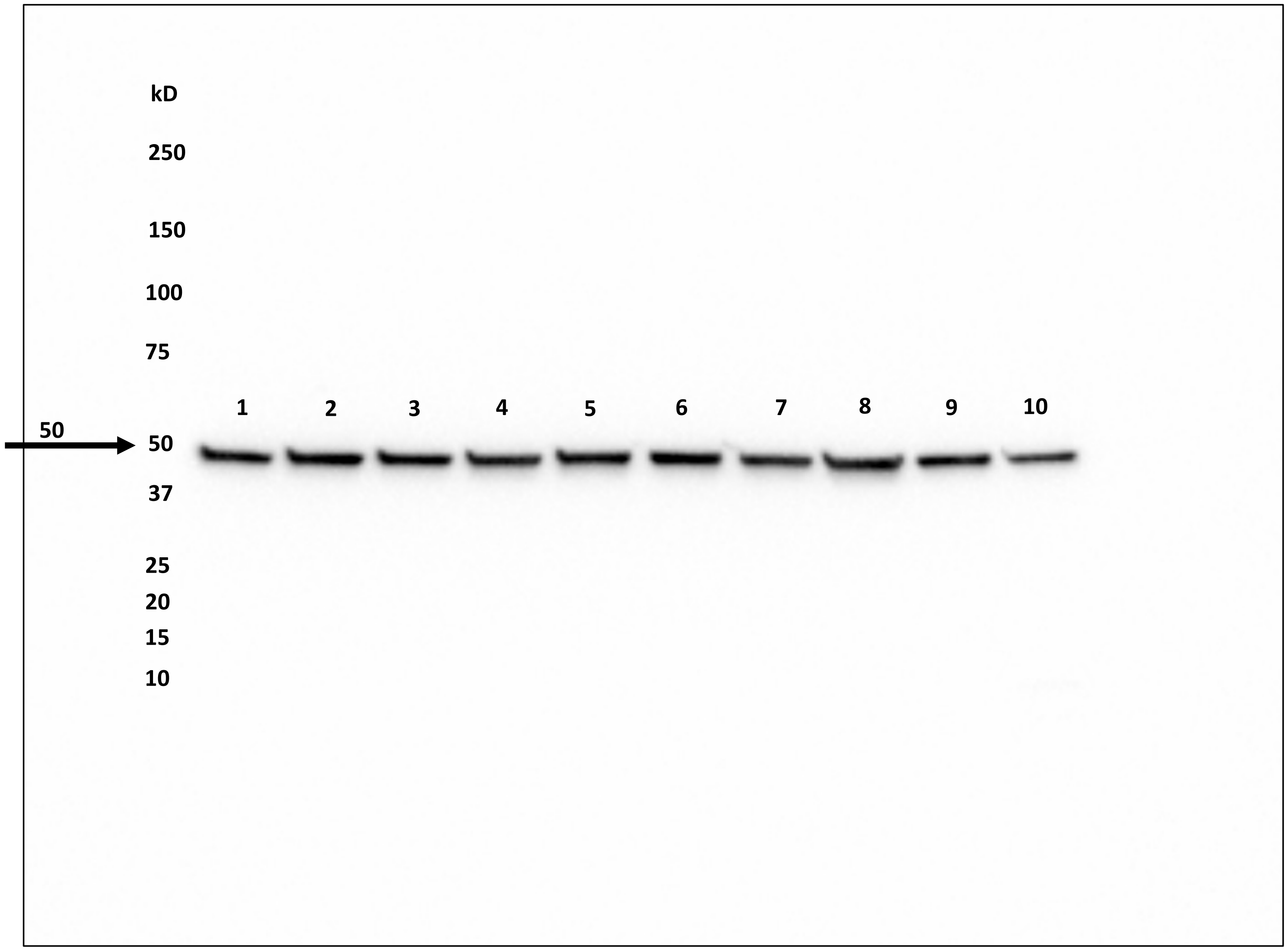

S5B Fig
Probing: LC3B (PA1-46286)
Life Technologies

Loading order:
Lane 1: Control at 72 h
Lane 2: 24h 7mM DHA
Lane 3: 48h 7mM DHA
Lane 4: 72 h 7mM DHA
Lane 5: 24h 10 nM Rapamycin
Lane 6: 48h 10 nM Rapamycin
Lane 7: 72h 10 nM Rapamycin
Lane 8: 24h 7 mM DHA and 10 nM Rapamycin
Lane 9: 48h 7 mM DHA and 10 nM Rapamycin
Lane 10: 72h 7 mM DHA and 10 nM Rapamycin

Imaged with Bio-Rad ChemiDoc XRS Imaging system
Chemi Hi Sensitivity setting

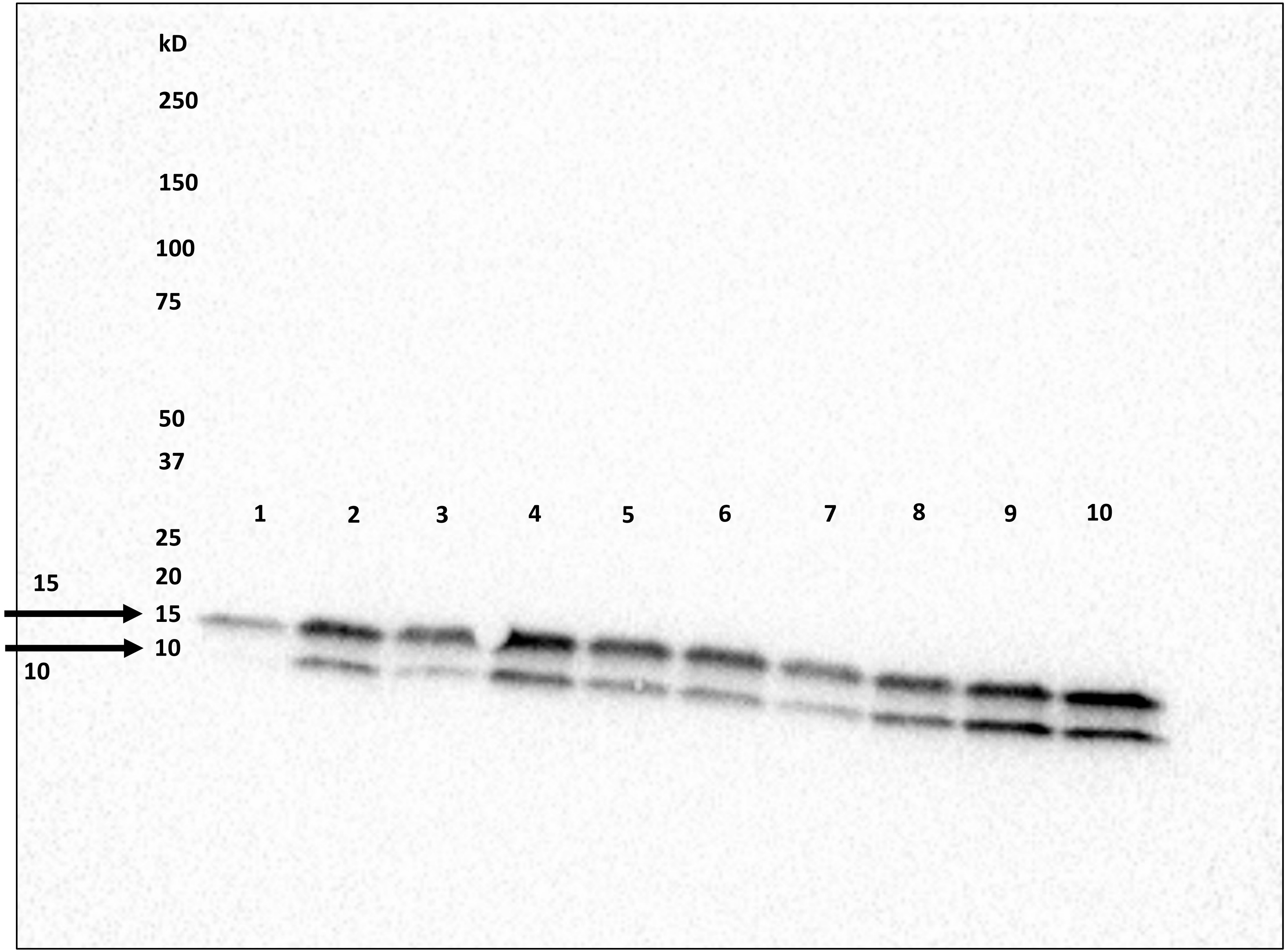

S5B Fig
Probing: α-Tubulin (T9026)
Millipore Sigma

Loading order:
Lane 1: Control at 72 h
Lane 2: 24h 7mM DHA
Lane 3: 48h 7mM DHA
Lane 4: 72 h 7mM DHA
Lane 5: 24h 10 nM Rapamycin
Lane 6: 48h 10 nM Rapamycin
Lane 7: 72h 10 nM Rapamycin
Lane 8: 24h 7 mM DHA and 10 nM Rapamycin
Lane 9: 48h 7 mM DHA and 10 nM Rapamycin
Lane 10: 72h 7 mM DHA and 10 nM Rapamycin

Imaged with Bio-Rad ChemiDoc XRS Imaging system
Chemi Hi Sensitivity setting

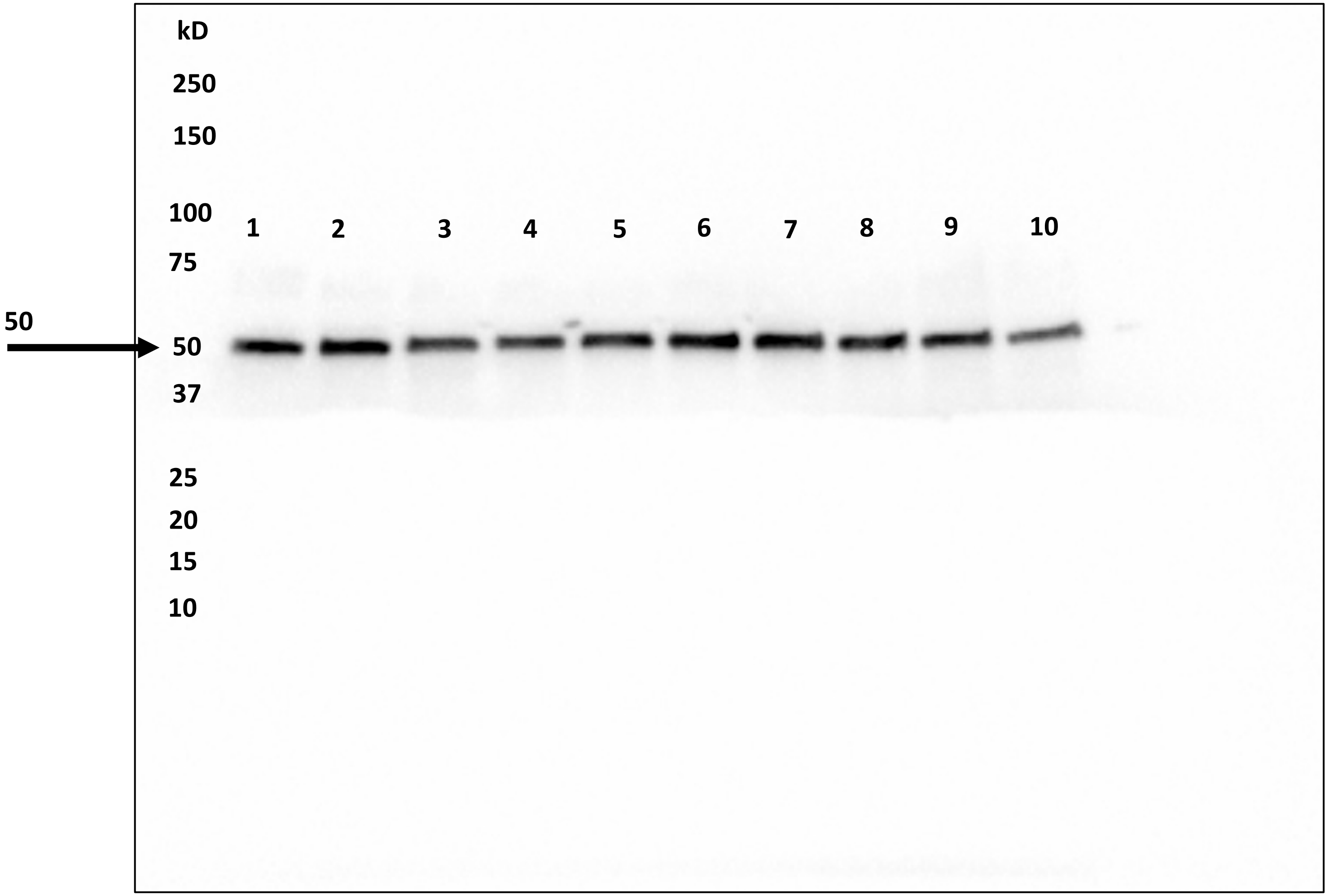

S5C Fig
Probing: Caspase-3 (GTX13585)
GeneTex

Loading order:
Lane 1: Control at 72 h
Lane 2: 24h 7mM DHA
Lane 3: 48h 7mM DHA
Lane 4: 72 h 7mM DHA
Lane 5: 24h 10 nM Rapamycin
Lane 6: 48h 10 nM Rapamycin
Lane 7: 72h 10 nM Rapamycin
Lane 8: 24h 7 mM DHA and 10 nM Rapamycin
Lane 9: 48h 7 mM DHA and 10 nM Rapamycin
Lane 10: 72h 7 mM DHA and 10 nM Rapamycin

Imaged with Bio-Rad ChemiDoc XRS Imaging system
Chemi Hi Sensitivity setting

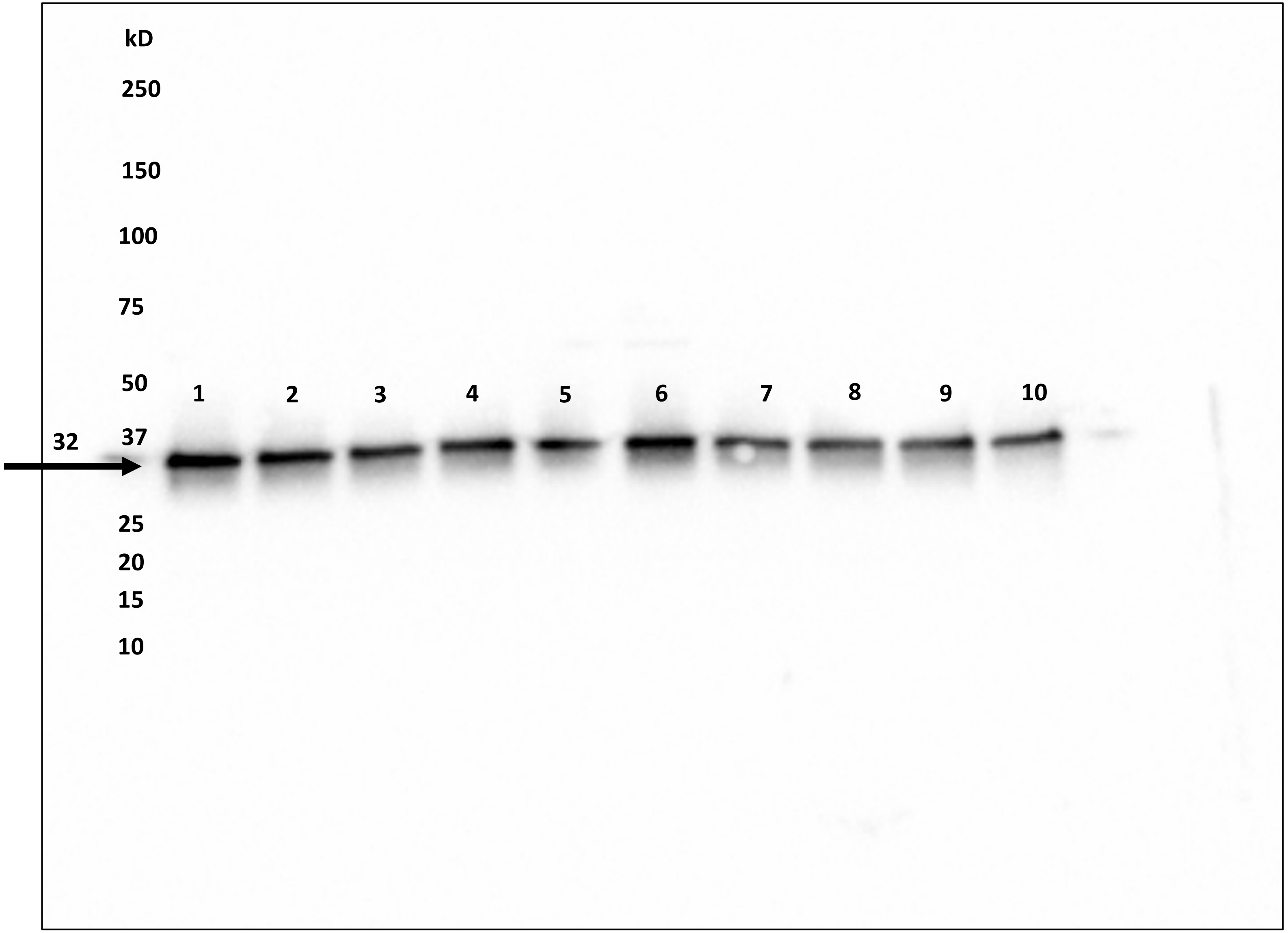

S5C Fig
Probing: α-Tubulin (T9026)
Millipore Sigma

Loading order:
Lane 1: Control at 72 h
Lane 2: 24h 7mM DHA
Lane 3: 48h 7mM DHA
Lane 4: 72 h 7mM DHA
Lane 5: 24h 10 nM Rapamycin
Lane 6: 48h 10 nM Rapamycin
Lane 7: 72h 10 nM Rapamycin
Lane 8: 24h 7 mM DHA and 10 nM Rapamycin
Lane 9: 48h 7 mM DHA and 10 nM Rapamycin
Lane 10: 72h 7 mM DHA and 10 nM Rapamycin

Imaged with Bio-Rad ChemiDoc XRS Imaging system
Chemi Hi Sensitivity setting

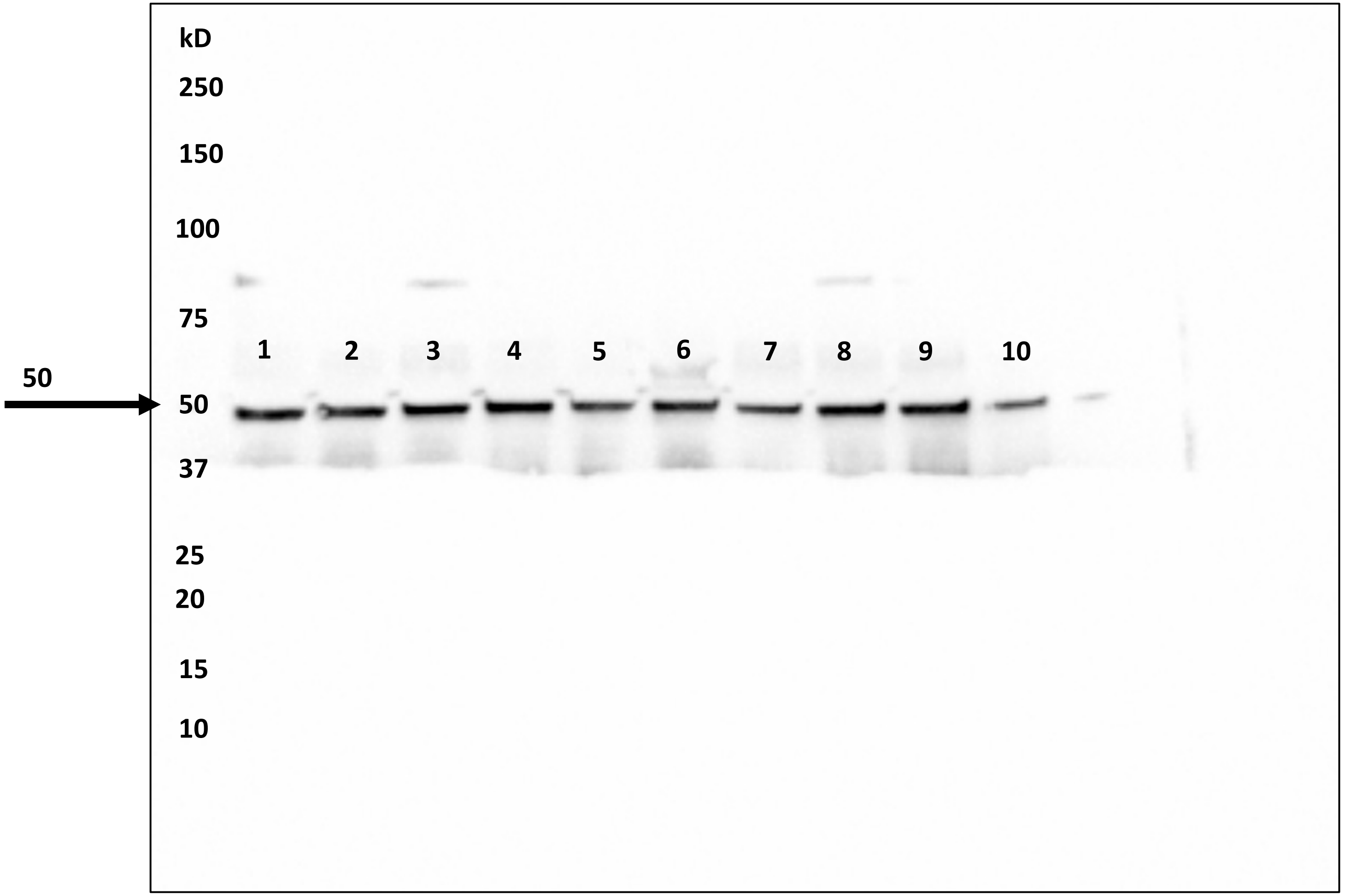

S5C Fig
Probing: PARP-1 (556494)
BD Biosciences

Loading order:
Lane 1: Control at 72 h
Lane 2: 24h 7mM DHA
Lane 3: 48h 7mM DHA
Lane 4: 72 h 7mM DHA
Lane 5: 24h 10 nM Rapamycin
Lane 6: 48h 10 nM Rapamycin
Lane 7: 72h 10 nM Rapamycin
Lane 8: 24h 7 mM DHA and 10 nM Rapamycin
Lane 9: 48h 7 mM DHA and 10 nM Rapamycin
Lane 10: 72h 7 mM DHA and 10 nM Rapamycin

Imaged with Bio-Rad ChemiDoc XRS Imaging system
Chemi Hi Sensitivity setting

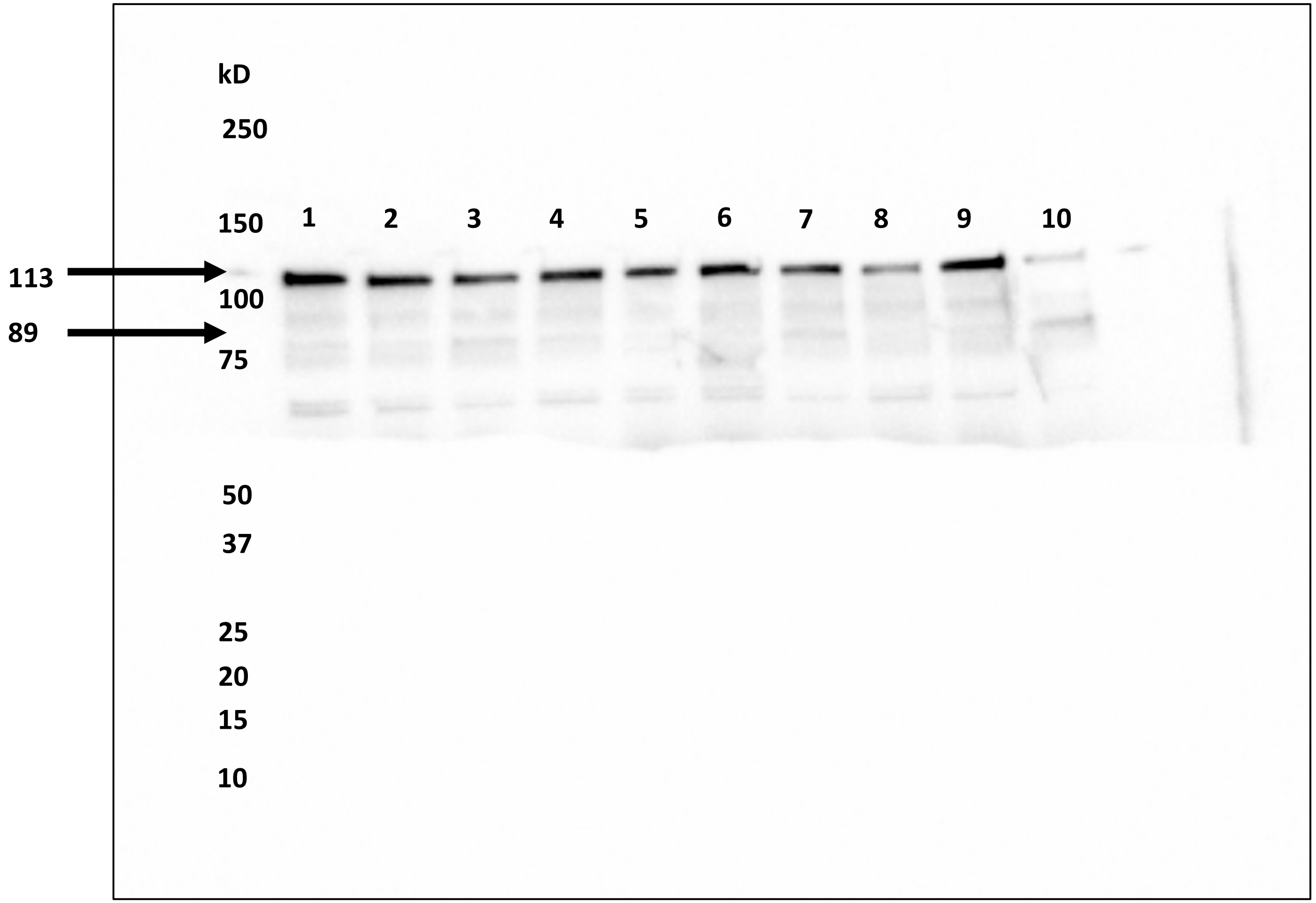

S5C Fig
Probing: α-Tubulin (T9026)
Millipore Sigma

Loading order:
Lane 1: Control at 72 h
Lane 2: 24h 7mM DHA
Lane 3: 48h 7mM DHA
Lane 4: 72 h 7mM DHA
Lane 5: 24h 10 nM Rapamycin
Lane 6: 48h 10 nM Rapamycin
Lane 7: 72h 10 nM Rapamycin
Lane 8: 24h 7 mM DHA and 10 nM Rapamycin
Lane 9: 48h 7 mM DHA and 10 nM Rapamycin
Lane 10: 72h 7 mM DHA and 10 nM Rapamycin

Imaged with Bio-Rad ChemiDoc XRS Imaging system
Chemi Hi Sensitivity setting

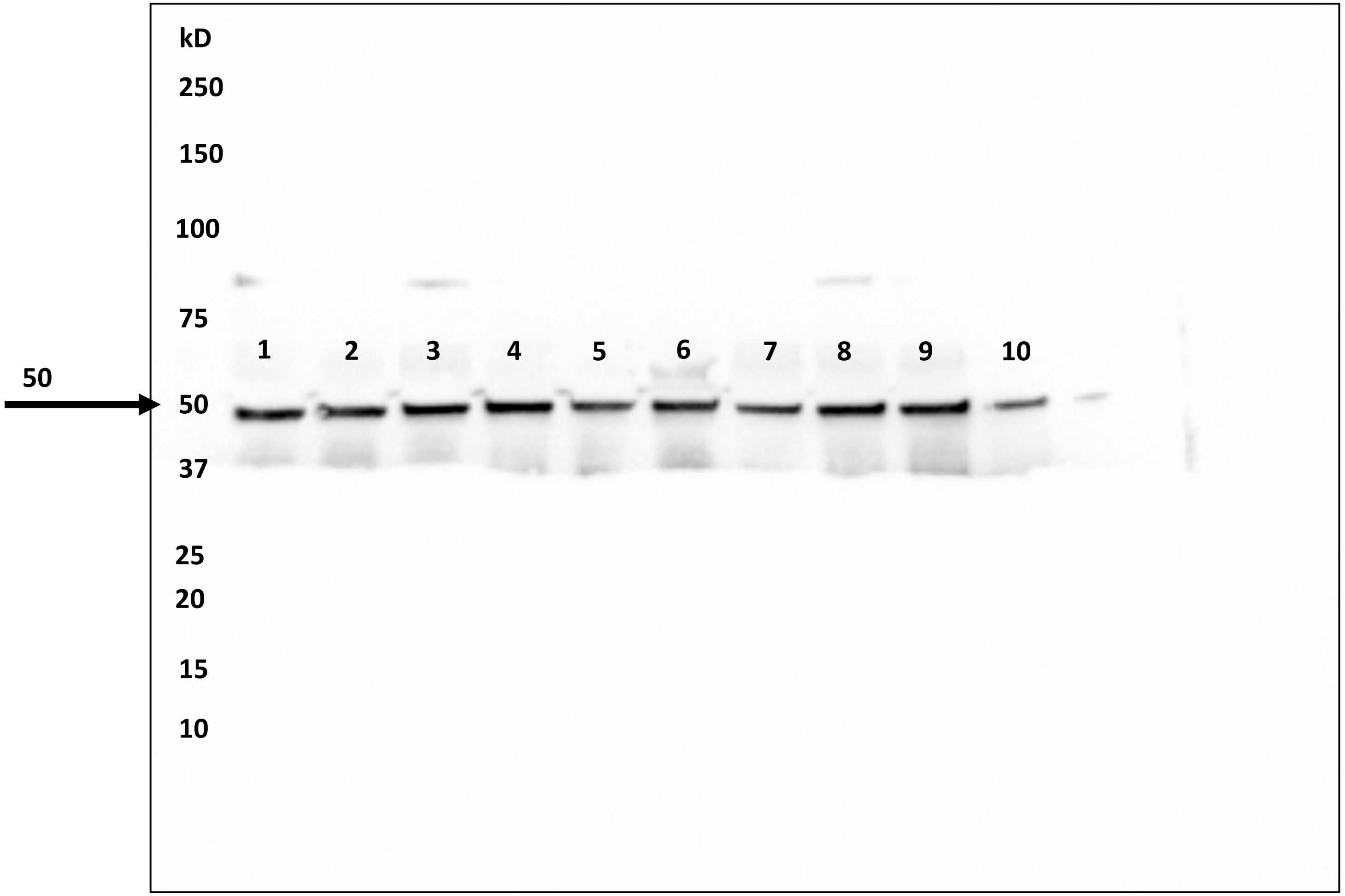

S6B Fig
Probing: LC3B (PA1-46286)
Life Technologies

Loading order:
Lane1: Control at 72 h
Lane 2: 24h 7mM DHA
Lane 3: 48h 7mM DHA
Lane 4: 72 h 7mM DHA
Lane 5: 24h 2.5 μM Chloroquine
Lane 6: 48h 2.5 μM Chloroquine
Lane 7: 72h 2.5 μM Chloroquine
Lane 8: 24h 7 mM DHA and 2.5 μM Chloroquine
Lane 9: 48h 7 mM DHA and 2.5 μM Chloroquine
Lane 10: 72h 7 mM DHA and 2.5 μM Chloroquine

Imaged with Bio-Rad ChemiDoc XRS Imaging system
Chemi Hi Sensitivity setting

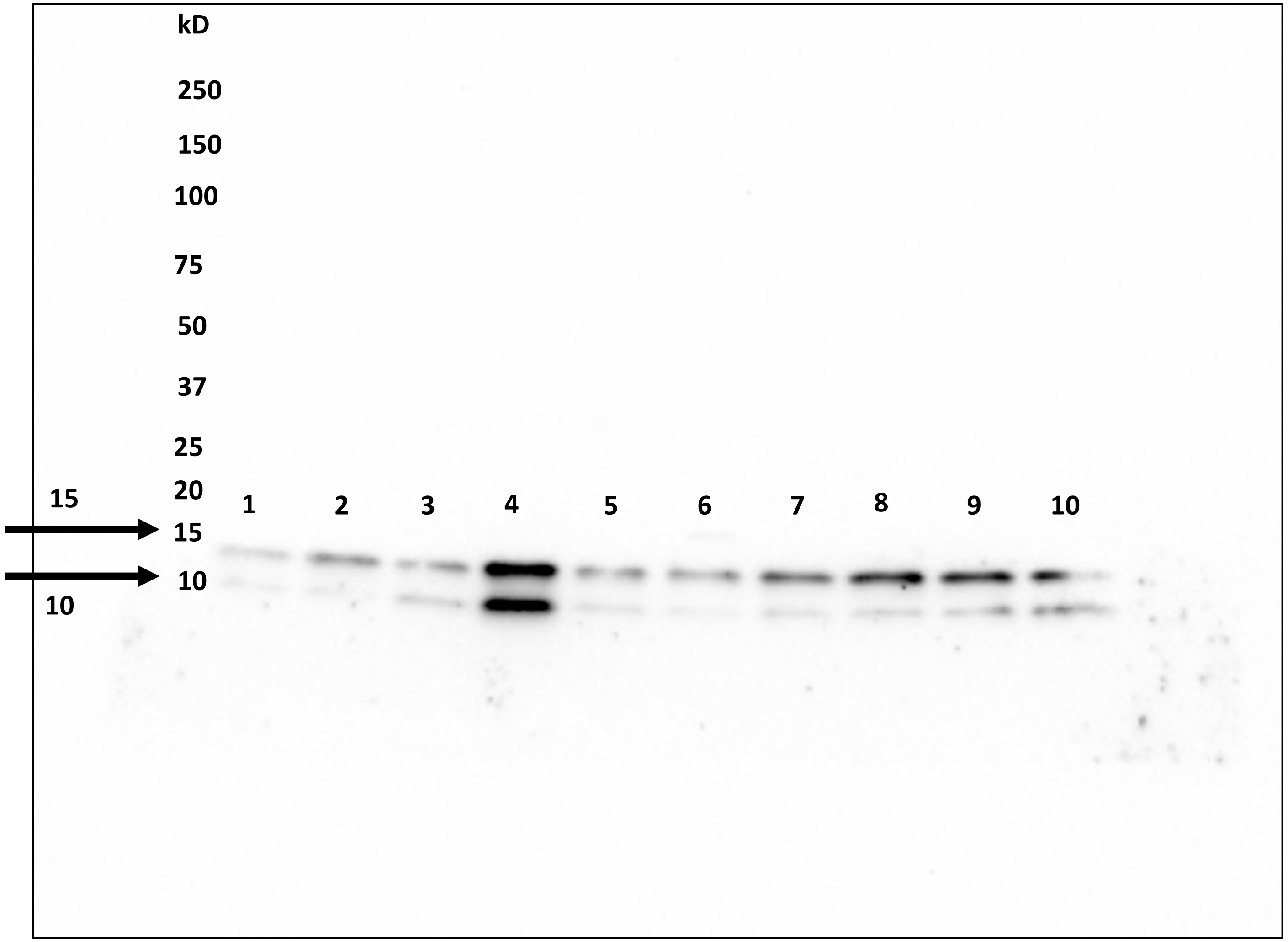

S6B Fig
Probing: α-Tubulin (T9026)
Millipore Sigma

Loading order:
Lane1: Control at 72 h
Lane 2: 24h 7mM DHA
Lane 3: 48h 7mM DHA
Lane 4: 72 h 7mM DHA
Lane 5: 24h 2.5 μM Chloroquine
Lane 6: 48h 2.5 μM Chloroquine
Lane 7: 72h 2.5 μM Chloroquine
Lane 8: 24h 7 mM DHA and 2.5 μM Chloroquine
Lane 9: 48h 7 mM DHA and 2.5 μM Chloroquine
Lane 10: 72h 7 mM DHA and 2.5 μM Chloroquine

Imaged with Bio-Rad ChemiDoc XRS Imaging system
Chemi Hi Sensitivity setting

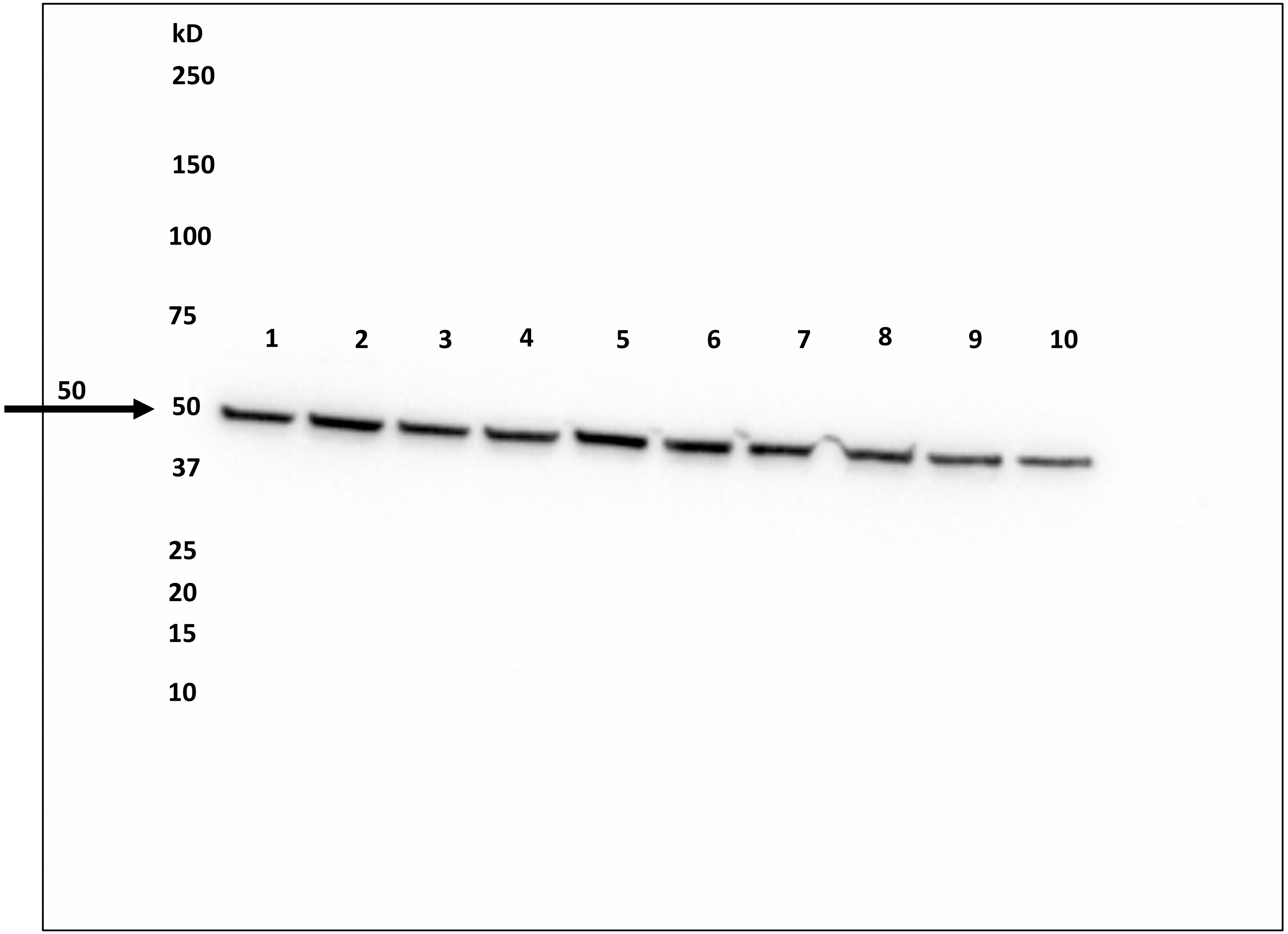

S6C Fig
Probing: Caspase-3 (GTX13585)
GeneTex

Loading order:
Lane1: Control at 72 h
Lane 2: 24h 7mM DHA
Lane 3: 48h 7mM DHA
Lane 4: 72 h 7mM DHA
Lane 5: 24h 2.5 μM Chloroquine
Lane 6: 48h 2.5 μM Chloroquine
Lane 7: 72h 2.5 μM Chloroquine
Lane 8: 24h 7 mM DHA and 2.5 μM Chloroquine
Lane 9: 48h 7 mM DHA and 2.5 μM Chloroquine
Lane 10: 72h 7 mM DHA and 2.5 μM Chloroquine

Imaged with Bio-Rad ChemiDoc XRS Imaging system
Chemi Hi Sensitivity setting

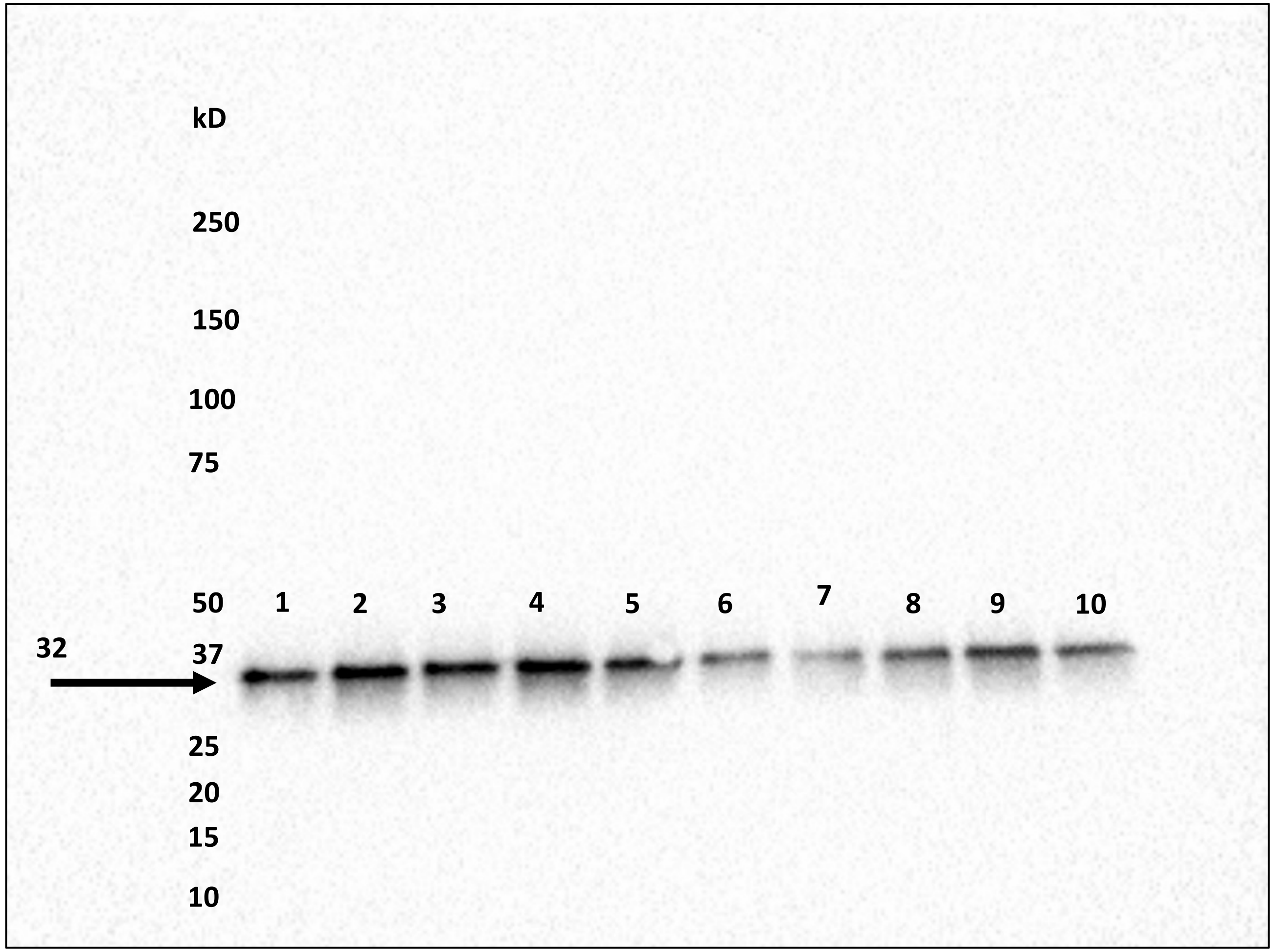

S6C Fig
Probing: α-Tubulin (T9026)
Millipore Sigma

Loading order:
Lane1: Control at 72 h
Lane 2: 24h 7mM DHA
Lane 3: 48h 7mM DHA
Lane 4: 72 h 7mM DHA
Lane 5: 24h 2.5 µM Chloroquine
Lane 6: 48h 2.5 µM Chloroquine
Lane 7: 72h 2.5 µM Chloroquine
Lane 8: 24h 7 mM DHA and 2.5 µM Chloroquine
Lane 9: 48h 7 mM DHA and 2.5 µM Chloroquine
Lane 10: 72h 7 mM DHA and 2.5 µM Chloroquine

Imaged with Bio-Rad ChemiDoc XRS Imaging system
Chemi Hi Sensitivity setting

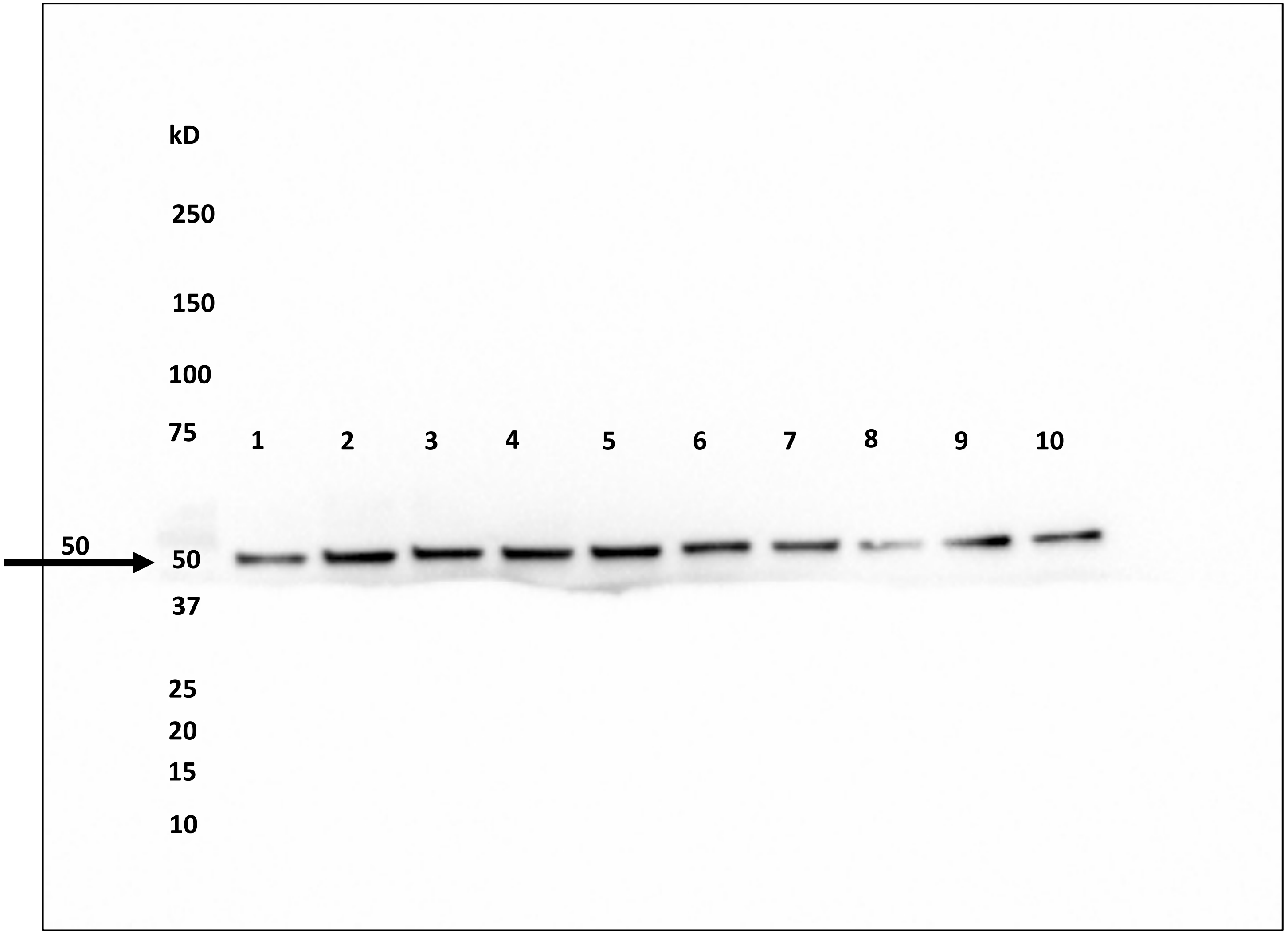

S6C Fig
Probing: PARP-1 (556494)
BD Biosciences

Loading order:
Lane1: Control at 72 h
Lane 2: 24h 7mM DHA
Lane 3: 48h 7mM DHA
Lane 4: 72 h 7mM DHA
Lane 5: 24h 2.5 μM Chloroquine
Lane 6: 48h 2.5 μM Chloroquine
Lane 7: 72h 2.5 μM Chloroquine
Lane 8: 24h 7 mM DHA and 2.5 μM Chloroquine
Lane 9: 48h 7 mM DHA and 2.5 μM Chloroquine
Lane 10: 72h 7 mM DHA and 2.5 μM Chloroquine

Imaged with Bio-Rad ChemiDoc XRS Imaging system
Chemi Hi Sensitivity setting

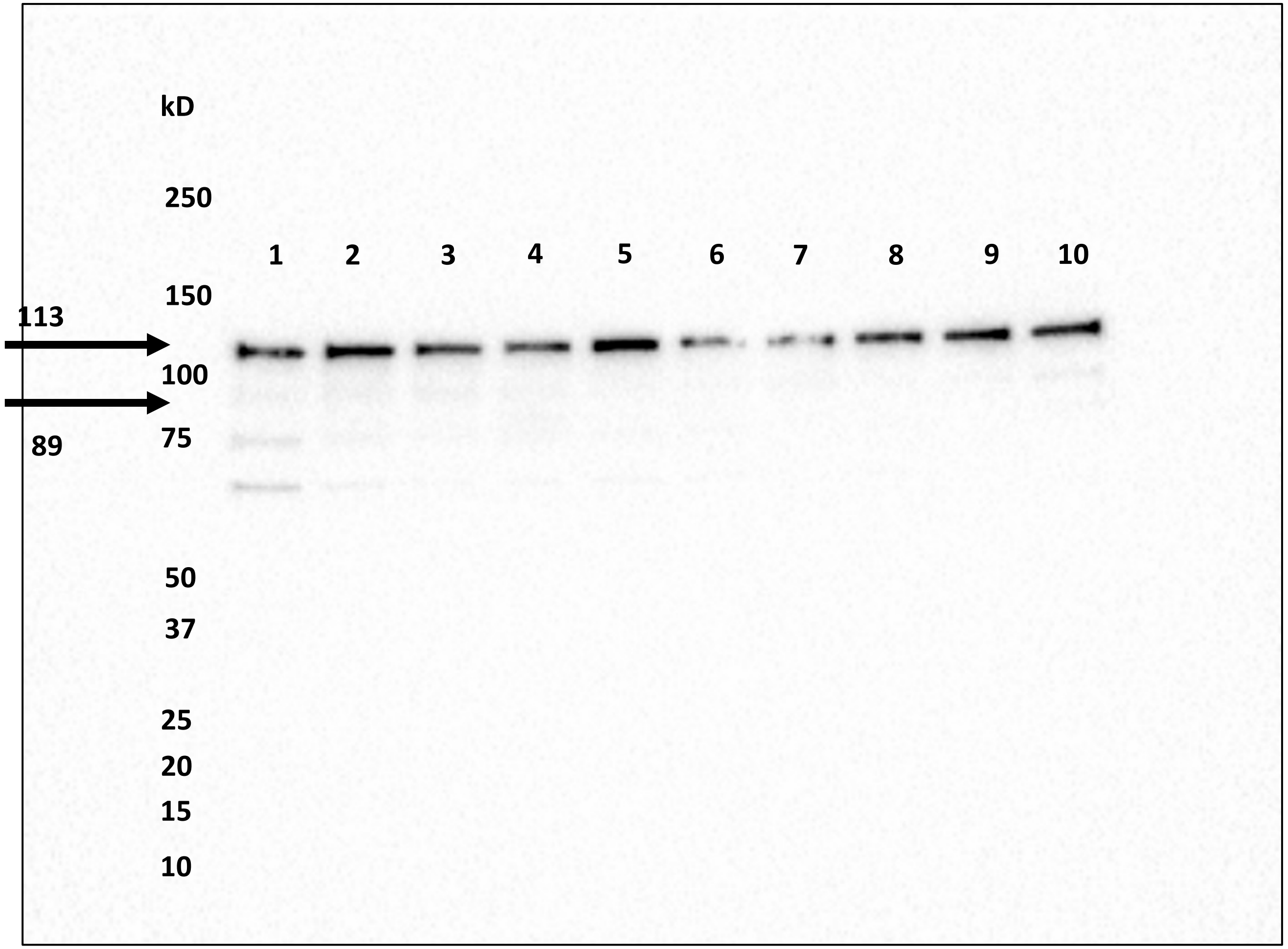

S6C Fig
Probing: α-Tubulin (T9026)
Millipore Sigma

Loading order:
Lane1: Control at 72 h
Lane 2: 24h 7mM DHA
Lane 3: 48h 7mM DHA
Lane 4: 72 h 7mM DHA
Lane 5: 24h 2.5 μM Chloroquine
Lane 6: 48h 2.5 μM Chloroquine
Lane 7: 72h 2.5 μM Chloroquine
Lane 8: 24h 7 mM DHA and 2.5 μM Chloroquine
Lane 9: 48h 7 mM DHA and 2.5 μM Chloroquine
Lane 10: 72h 7 mM DHA and 2.5 μM Chloroquine

Imaged with Bio-Rad ChemiDoc XRS Imaging system
Chemi Hi Sensitivity setting

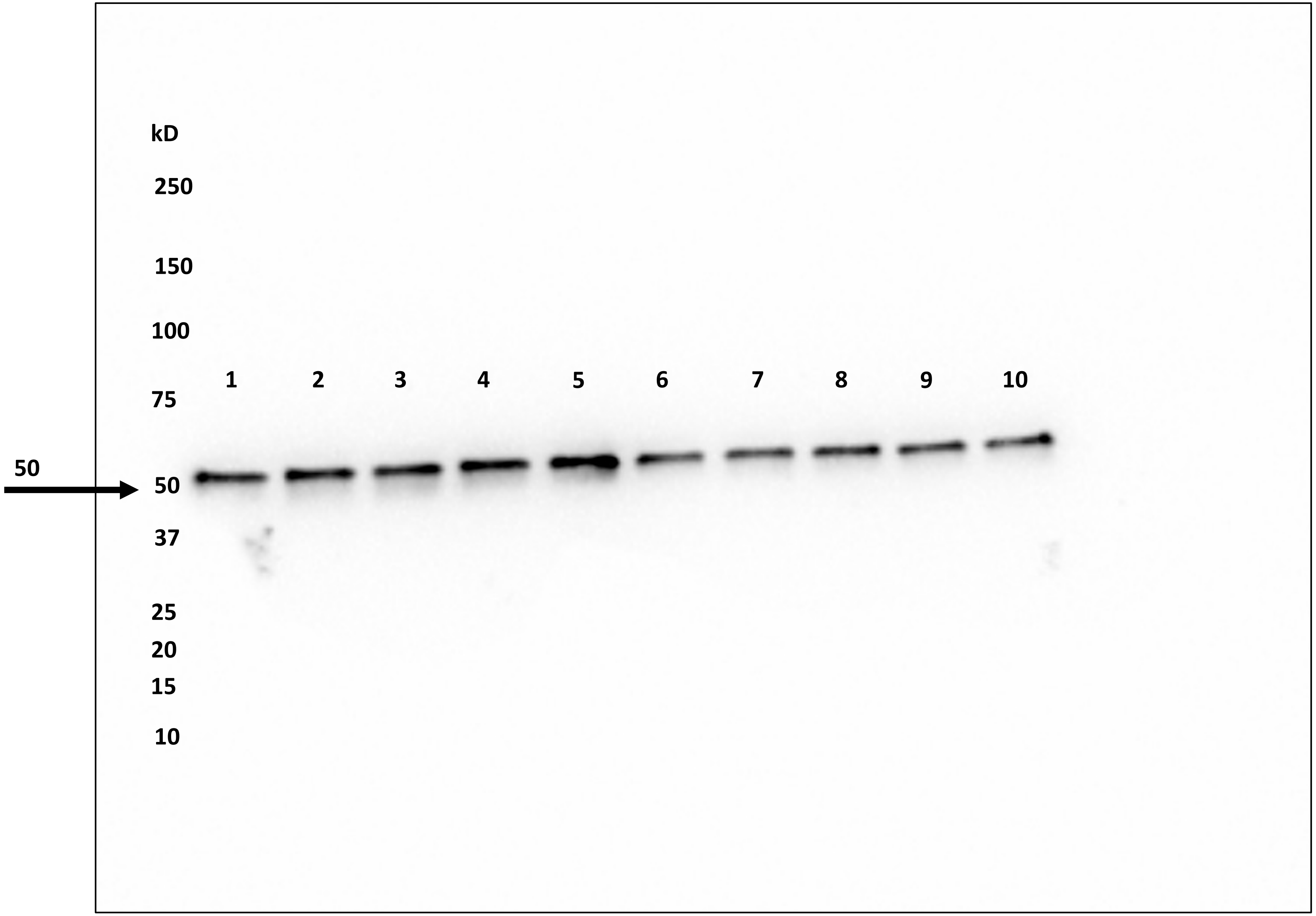

Figure S8 Fig
Probing: p-ULK1 Ser 555 (D1H4) (5869)
Cell Signaling Technologies

Loading order:
Lane 1: Control at 96 h
Lane 2: 24h 7mM DHA
Lane 3: 48h 7mM DHA
Lane 4: 72 h 7mM DHA
Lane 5: 96 h 7mM DHA
Lane 6: 24 h 1 μM μCPT
Lane 7: 48 h 1 μM μCPT

Imaged with Bio-Rad ChemiDoc XRS Imaging system
Chemi Hi Sensitivity setting

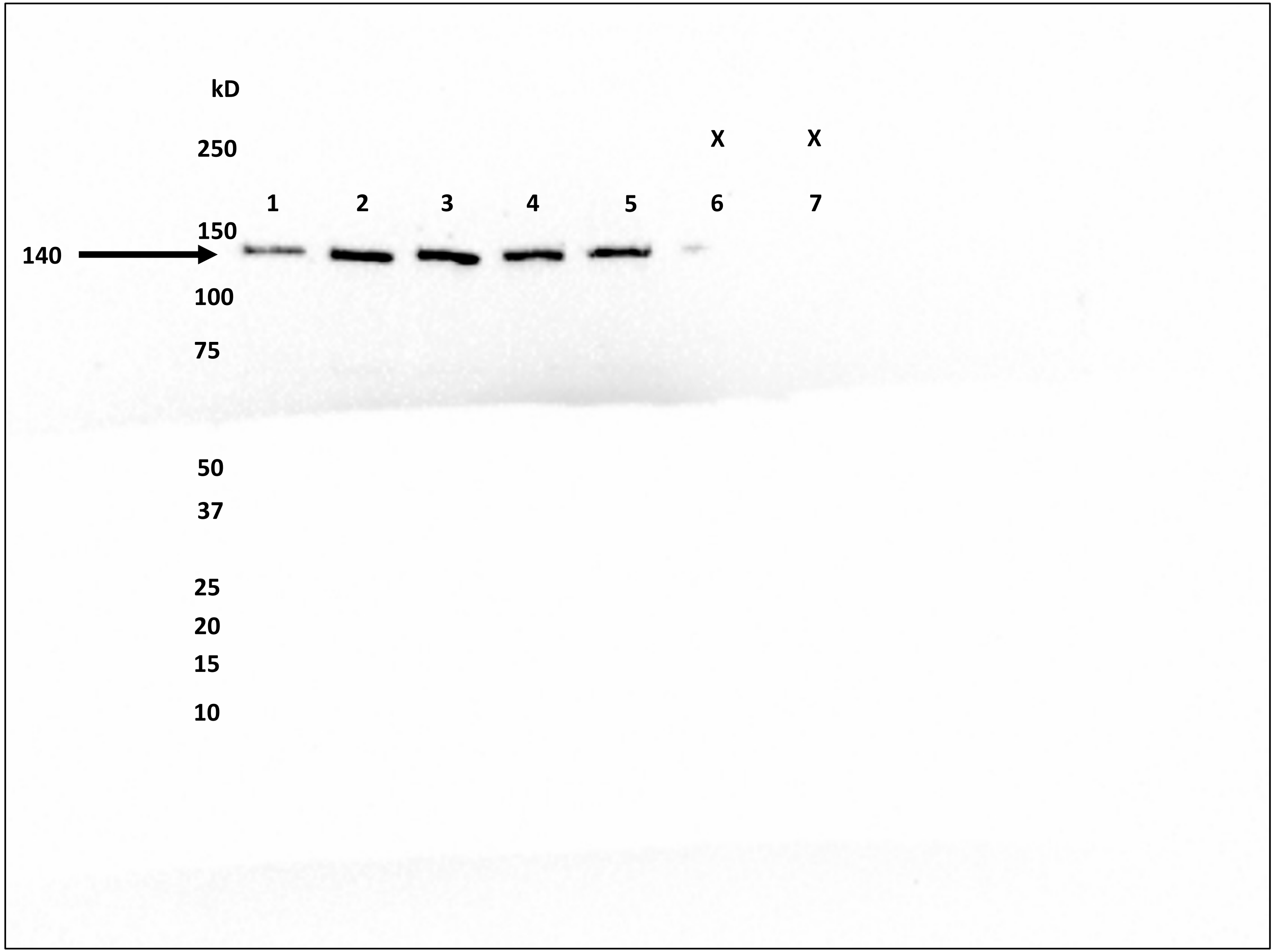

Figure S8 Fig
Probing: ULK1 (D8H5) (8054)
Cell Signaling Technologies

Loading order:
Lane 1: Control at 96 h
Lane 2: 24h 7mM DHA
Lane 3: 48h 7mM DHA
Lane 4: 72 h 7mM DHA
Lane 5: 96 h 7mM DHA
Lane 6: 24 h 1 μM μCPT
Lane 7: 48 h 1 μM μCPT

Imaged with Bio-Rad ChemiDoc XRS
Imaging system
Chemi Hi Sensitivity setting

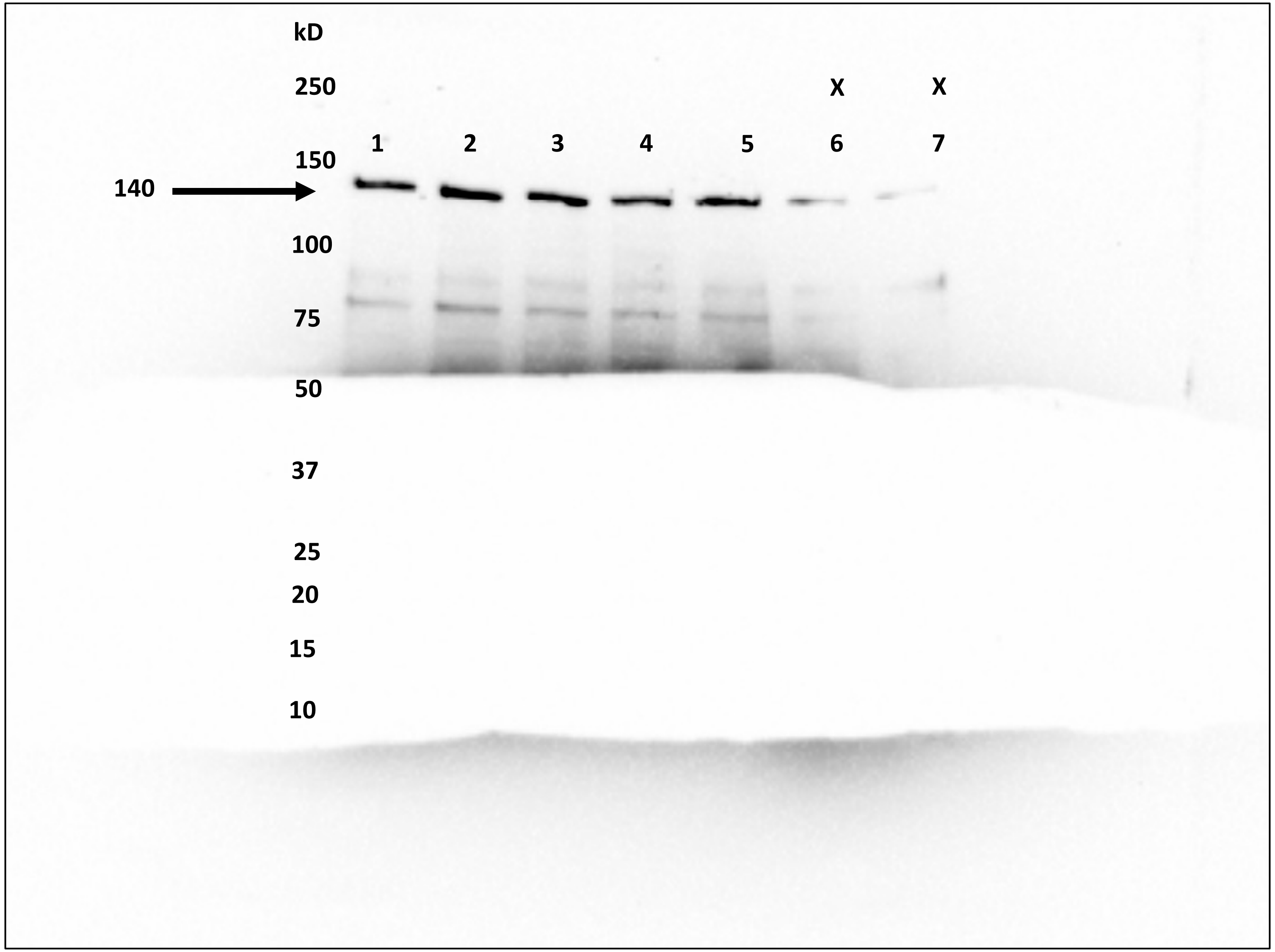

Figure S8 Fig
Probing: α-Tubulin (T9026)
Millipore Sigma

Loading order:
Lane 1: Control at 96 h
Lane 2: 24h 7mM DHA
Lane 3: 48h 7mM DHA
Lane 4: 72 h 7mM DHA
Lane 5: 96 h 7mM DHA
Lane 6: 24 h 1 μM μCPT
Lane 7: 48 h 1 μM μCPT

Imaged with Bio-Rad ChemiDoc XRS
Imaging system
Chemi Hi Sensitivity setting

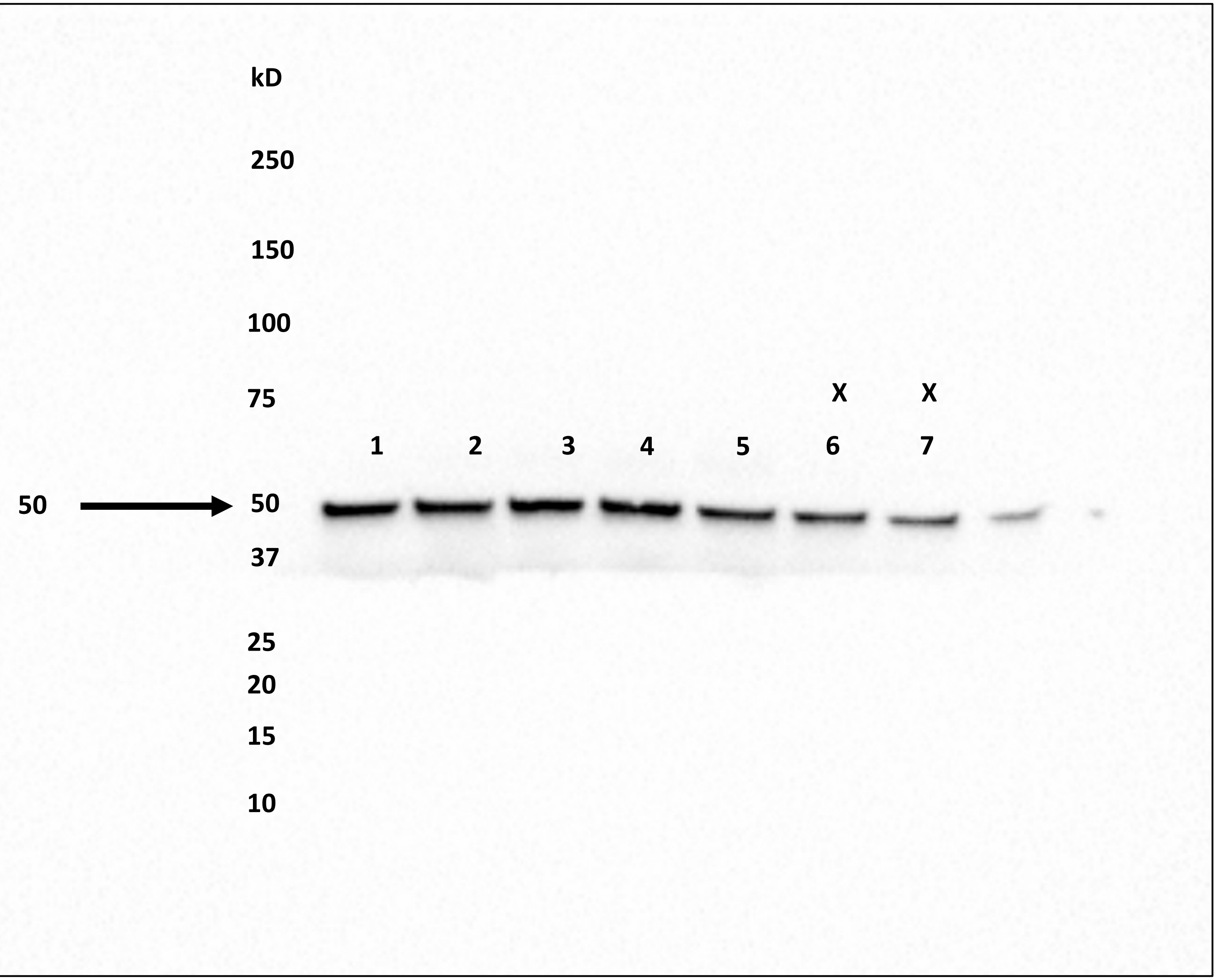

Figure S9 Fig
Probing: p-4E-BP1 (Thr 37/46)(236B4) (2855)
Cell Signaling

Loading order:
Lane 1: Control at 24 h
Lane 2: 1h 7mM DHA
Lane 3: 4h 7mM DHA
Lane 4: 24 h 7mM DHA

Imaged with Bio-Rad ChemiDoc XRS Imaging system
Chemi Hi Sensitivity setting

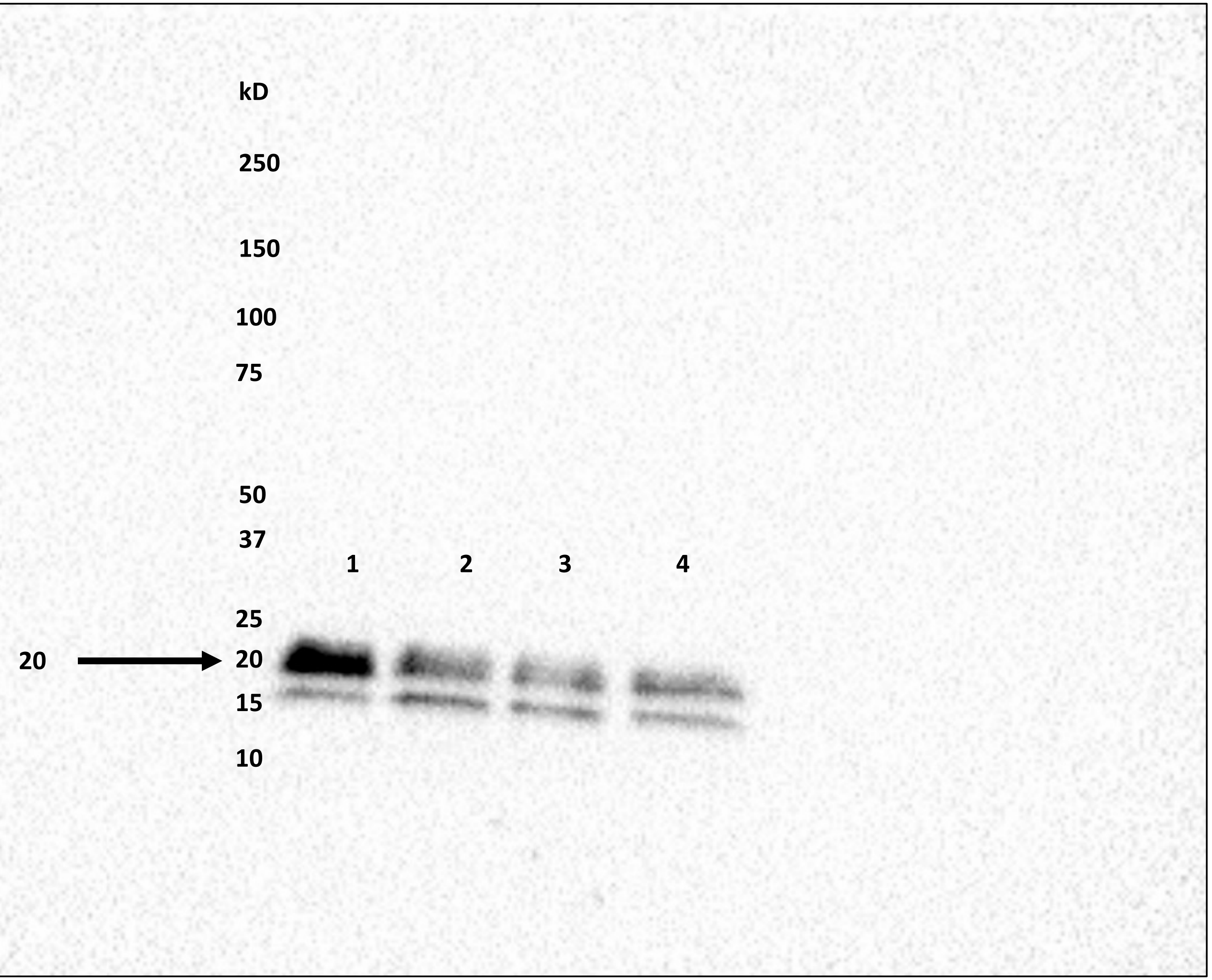

Figure S9 Fig
Probing: 4E-BP1 (53H11) (9644)
Cell Signaling

Loading order:
Lane 1: Control at 24 h
Lane 2: 1h 7mM DHA
Lane 3: 4h 7mM DHA
Lane 4: 24 h 7mM DHA

Imaged with Bio-Rad ChemiDoc XRS
Imaging system
Chemi Hi Sensitivity setting

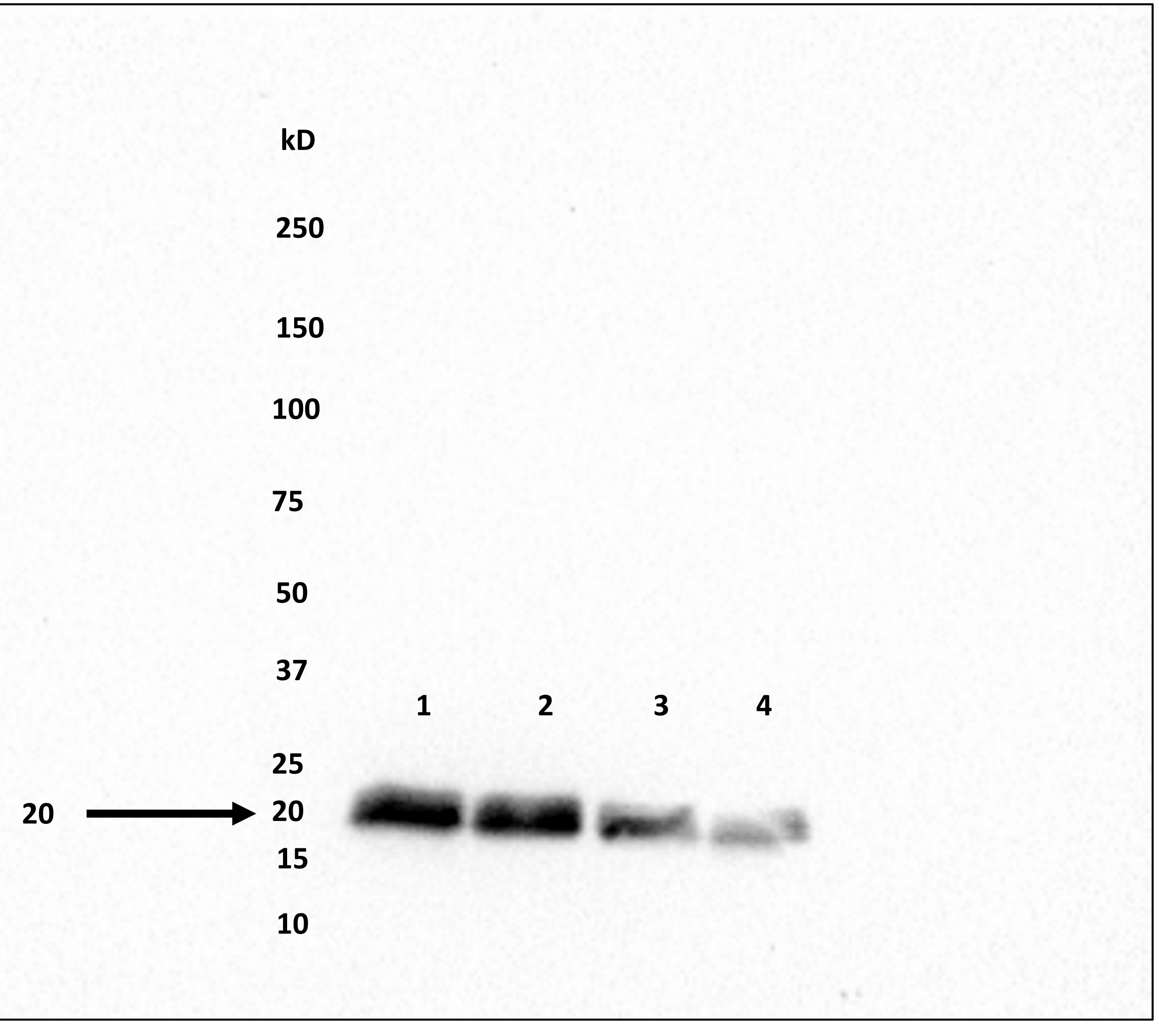

Figure S9 Fig
Probing: α-Tubulin (T9026)
Millipore Sigma

Loading order:
Lane 1: Control at 24 h
Lane 2: 1h 7mM DHA
Lane 3: 4h 7mM DHA
Lane 4: 24 h 7mM DHA

Imaged with Bio-Rad ChemiDoc XRS
Imaging system
Chemi Hi Sensitivity setting

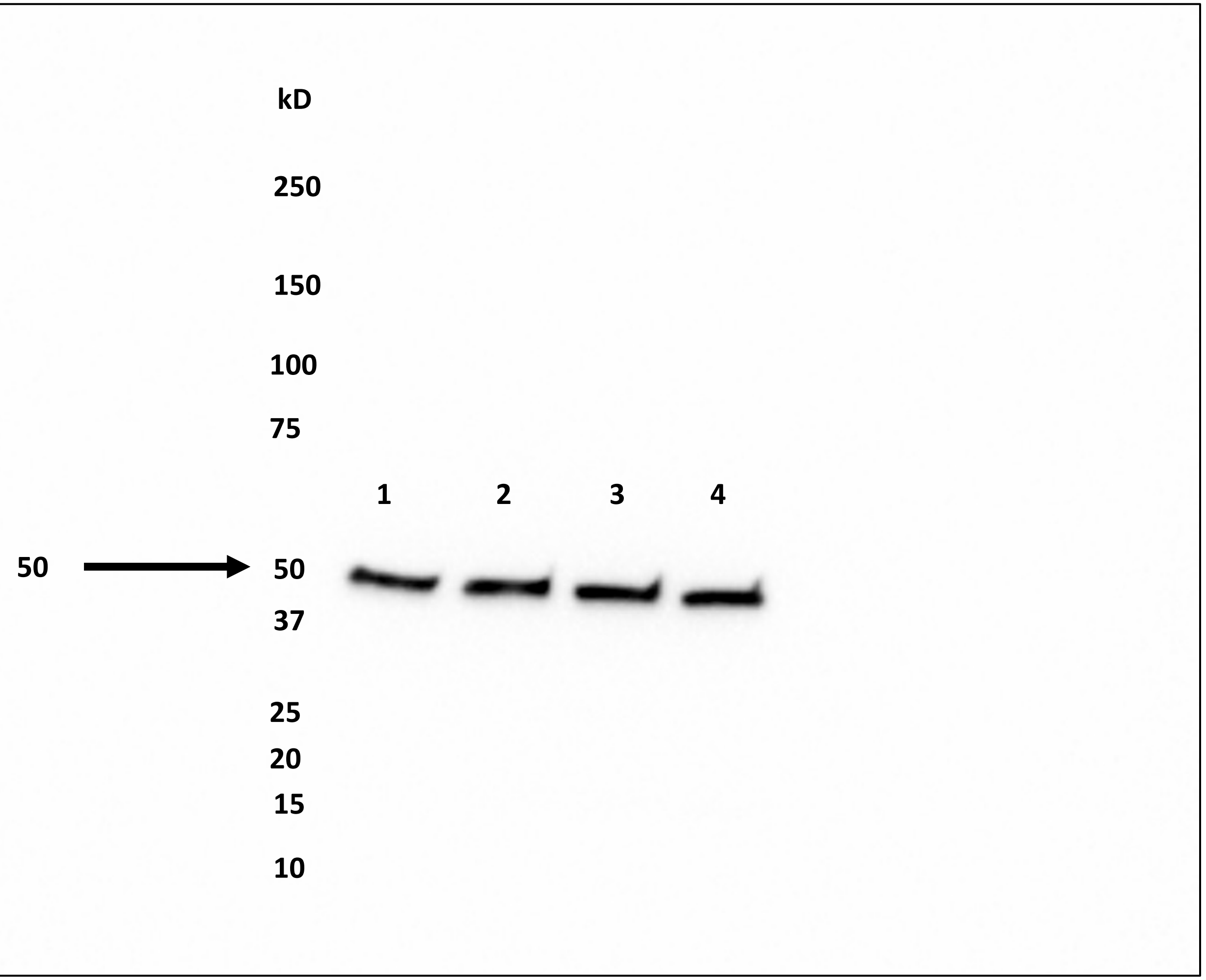

Supplement: S1 File — (PDF) [file pone.0278516.s011.pdf]
